# Dibenzo[*b*,*f*]azocin-6(5*H*)-ones and Benzo[*b*]pyrido[2,3-*f*]azocin-5(6*H*)-ones: Useful Scaffolds for Development of Protein–Protein Interaction Inhibitors †

**DOI:** 10.3390/ijms27187998

**Published:** 2026-09-08

**Authors:** Michał Nowacki, Filipe Menezes, Başak Dağdeviren, Federico Ballabio, Valeria Napolitano, Chethan K. Krishna, Vishal C. Kalel, Ralf Erdmann, Michael Sattler, Grzegorz M. Popowicz, Maciej Dawidowski

**Affiliations:** 1 Department of Drug Technology and Pharmaceutical Biotechnology, Medical University of Warsaw, Banacha 1, 02-097 Warszawa, Poland; 2 Institute of Structural Biology, Molecular Targets and Therapeutics Center, Helmholtz Zentrum München, Ingolstädter Landstraße 1, 85764 Neuherberg, Germany; 3 Institute of Biochemistry and Pathobiochemistry, Department of Systems Biochemistry, Faculty of Medicine, Ruhr-University Bochum, 44780 Bochum, Germany; chethan.krishna@ruhr-uni-bochum.de (C.K.K.);; 4 Bavarian NMR Center, Department of Bioscience, TUM School of Natural Sciences, Technical University of Munich, Lichtenbergstrasse 4, 85747 Garching, Germany

**Keywords:** dibenzo[*b*,*f*]azocin-6(5*H*)-one, benzo[*b*]pyrido[2,3-*f*]azocin-5(6*H*)-one, protein–protein interaction inhibitors, antiparasitic agents, structure-based drug design, quantum mechanical energy decomposition and deconvolution analysis

## Abstract

Modulation of protein–protein interactions (PPIs) by small molecules is a vital strategy in drug discovery. Nevertheless, due to the inherent nature of PPI interfaces, practical implementation of this approach remains a high-hanging fruit in medicinal chemistry. In this report, we develop a new line of PEX14–PEX5 PPI inhibitors by homologation of a previously developed dibenzo[*b*,*e*]azepin-6(6*H*)-one scaffold. The challenging chemistry of 8-membered ring formation led to the development of novel PEX14 inhibitors featuring a 6+8+6 tricyclic system. The obtained analogs were tested for their capability of disrupting the PEX5-TbPEX14 PPI, as well as for in vitro activity against the *T. brucei* protist. Overall, the developed scaffold not only represents an interesting alternative for prospective trypanocidal TbPEX14 ligands but may also be useful in the design of inhibitors of other PPIs mediated by a similar topology of hydrophobic binding pockets.

## 1. Introduction

Protein–protein interactions (PPIs) are ubiquitous in living organisms. They mediate crucial biochemical and signaling pathways, including apoptosis, cell differentiation, growth, metabolism, and intracellular transport. Consequently, the selective modulation of proteins’ ability to form complexes is an attractive strategy for combating human disease and creating tools for studying cellular processes [1,2,3]. Despite many efforts and developments that have been made in the field, PPI modulation remains a ‘high-hanging fruit’ in medicinal chemistry. This is due to the nature of the interfaces through which proteins form complexes, which are composed mostly of large, flat, solvent-exposed surface areas. Contrary to ‘classical’ drug targets, such as enzymes and receptors, which have well-developed, deeply buried binding sites for small molecules, the binding pockets that mediate PPIs are typically shallow, solvent-exposed, and distributed over a wide surface area. Together, these characteristics render compound design and the proper addressing of ADMET profiles extremely challenging. Therefore, new strategies are needed to develop PPI modulators [4,5].

**Figure 1:**
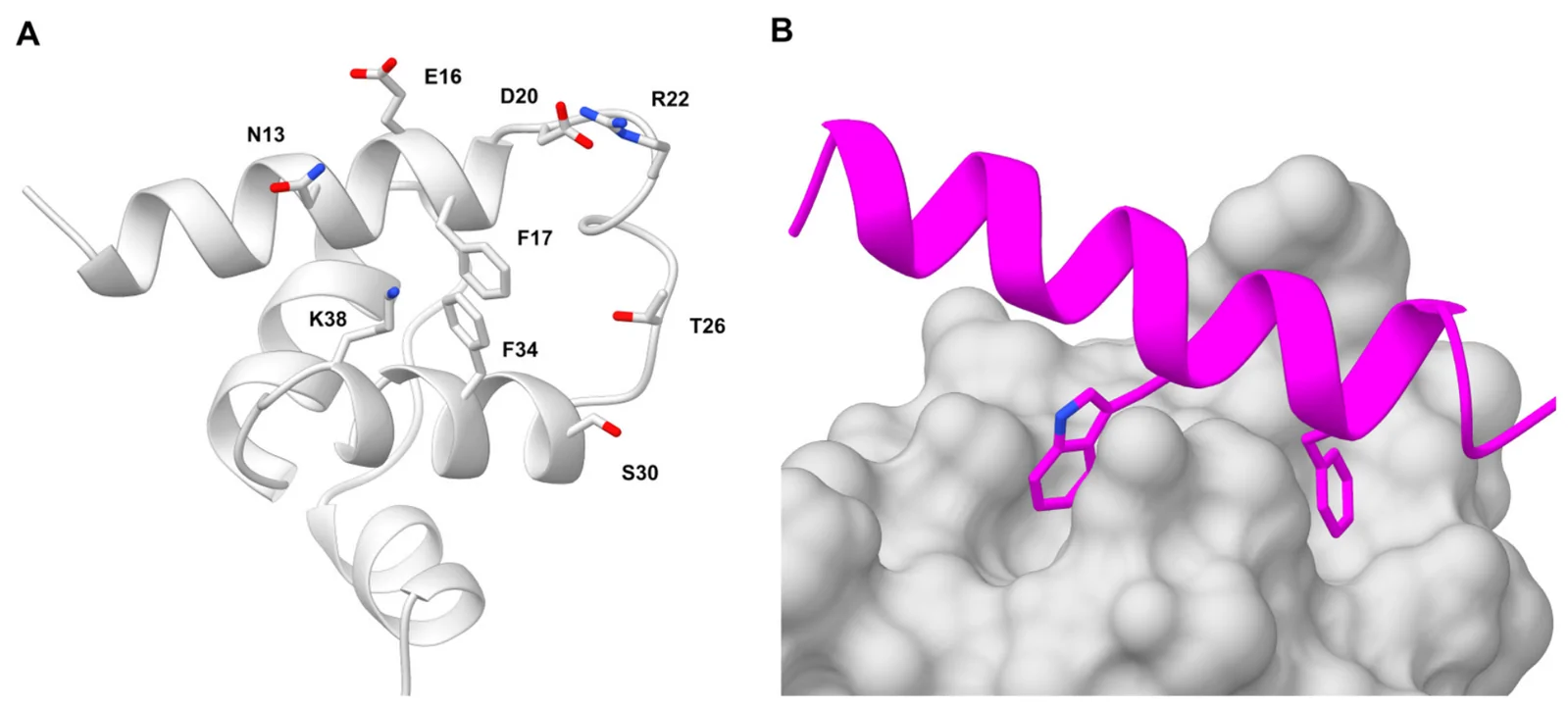
(**A**) Structure of TbPEX14 NTD (derived from PDB accession code: 5L87). The most important amino acid residues on the TbPEX14 NTD surface are shown. (**B**) NMR model of complex formation between the PEX5 helix (magenta) and PEX14 surface (gray) complex formation mediated by the WxxxF repeats (derived from the PDB accession code: 2W84).

In our previous reports, we have identified the first inhibitors of the PEX14-PEX5 PPI that impair glycosomal biogenesis in *Trypanosoma* protists, which are pathogens of lethal human diseases, such as the Human African Trypanosomiasis (HAT) and Chagas Disease [6,7,8,9,10,11]. Inhibition of this PPI poses challenges typical of this class of molecular targets. One specific structural feature that enables inhibitor design of this PPI is binding the Trp and Phe hydrophobic pockets on the PEX14 surface by PEX5 with the repeated WxxxF motifs (where ‘x’ denotes ‘any’ amino acid residue) of the α-helix (Figure 1). A unique characteristic of this binding site, which further aids ligand design, is a diaromatic bridge separating the two pockets formed by the stacked benzene rings of the F16-F33 amino acid residues [12].

**Figure 2:**
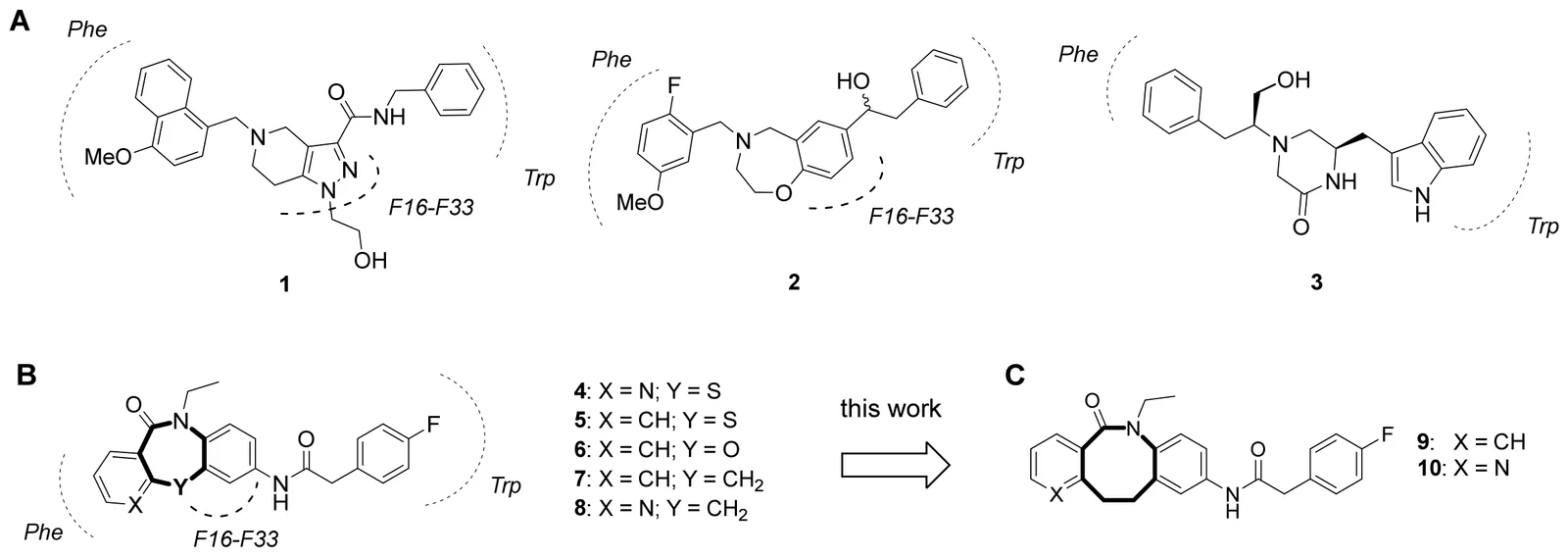
Previously developed chemical classes of PEX5-PEX14 PPI inhibitors. The common binding modes are illustrated: the aromatic residues mimic the native binding of PEX5 WxxxF fragments to PEX14 Trp and Phe pockets, and the central core interacts with the solvent-exposed F16 and F33 residues. (**A**) Examples of pyrazolo[4,3-*c*]pyridine **1**, 2,3,4,5-tetrahydrobenzo[*f*][1,4]oxazepine **2** and 2-oxopiperazine **3** derivatives. (**B**) Examples of tricyclic benzo[*b*]pyrido[3,2-*f*][1,4]thiazepin-5(6*H*)-one **4**, dibenzo[*b*,*f*][1,4]thiazepin-11(10*H*)-one **5** and dibenzo[*b*, *f*][1,4]oxazepin-11(10*H*)-one **6** [9], and dibenzo[*b*,*e*]azepin-6(6*H*)-one **7** and benzo[*b*]pyrido[2,3-*e*]azepin-5(5*H*)-one **8** derivatives [10]. (**C**) Dibenzo[*b*,*f*]azocin-6(5*H*)-ones **9** and benzo[*b*]pyrido[2,3-*f*]azocin-5(6*H*)-ones **10** designed in this study.

**Figure 3:**
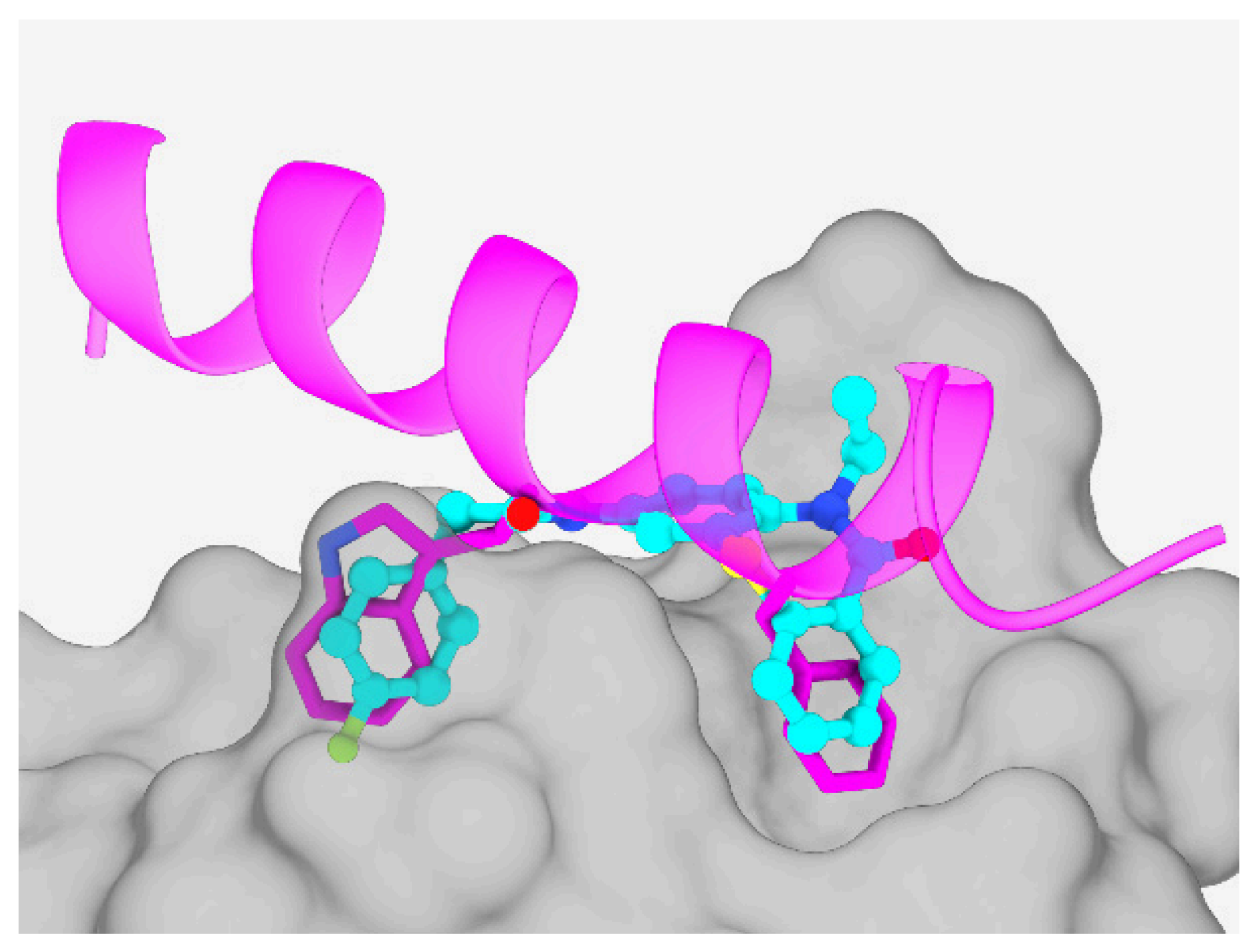
Comparison of the binding pose of compound **4** (blue) at the binding site of TcPEX14 (grey, PDB accession code: 7QRC) and the PEX5 WxxxF motif (magenta) with HsPEX14 NTD (PDB accession code: 2W84). The inhibitor mimics the spatial orientation of the aromatic residues in native WxxxF motifs.

By employing various structure- and ligand-based strategies, 3D pharmacophore screening [7], chemically advanced template search (CATS) algorithms [8], and peptidomimetic design [11], we developed inhibitors **1**–**3**, the first class for the PEX14-PEX5 PPI, impairing glycosomal biogenesis and killing *Trypanosoma* protists (Figure 2A). A very interesting class of tricyclic, small-molecule type D peptidomimetics (according to the classification proposed by Pelay-Gimeno and co-workers) emerged initially from a high-throughput screening (HTS) campaign (compounds **4**–**8**) using AlphaScreen and biomolecular NMR (Figure 2B) [9,10]. According to X-ray crystallography and docking, these compounds mimicked the binding mode of PEX5 WxxxF sequences to the Trp and Phe binding pockets on the surface of the TcPEX14 N-terminal domain (NTD; Figure 3). The key features of the binding mode of these compounds to the TcPEX14 NTD were the terminal aromatic ring of the 6+7+6 tricyclic scaffold filling the Phe pocket, the benzene moiety *t*-stacked with the diaromatic F16-F33 bridge of TcPEX14, and the central 7-membered ring adopting the bent conformation required for the desired spatial orientation of the binding residues. Additionally, the aniline nitrogen attached to the benzene ring of the heterocyclic core served as a suitable exit vector projecting the aromatic residues toward the hydrophobic Trp pocket of TcPEX14.

We have developed five chemical classes of tricyclic derivatives **4**–**8** [9,10] with 6+7+6 heterocyclic systems to target the PEX14-PEX5 PPI (Figure 2B). In this study, we pose the question whether homologation of the 7-membered ring in **7** and **8** leading to the respective dibenzo[*b*,*f*]azocin-6(5*H*)-one **9** and benzo[*b*]pyrido[2,3-*f*]azocin-5(6*H*)-one **10** scaffolds with a slightly more flexible 8-membered central ring can deliver another class of molecules to target the PEX14-PEX5 PPI interface (Figure 2C). To accomplish this goal, we explore the challenging chemistry of 8-membered ring formation, followed by in vitro binding evaluation and in silico analysis. Finally, we evaluate whether the transition from 6+7+6 to 6+8+6 ring systems affects the compounds’ cellular activity.

## 2. Results and Discussion

### 2.1. Compound Design

**Figure 4:**
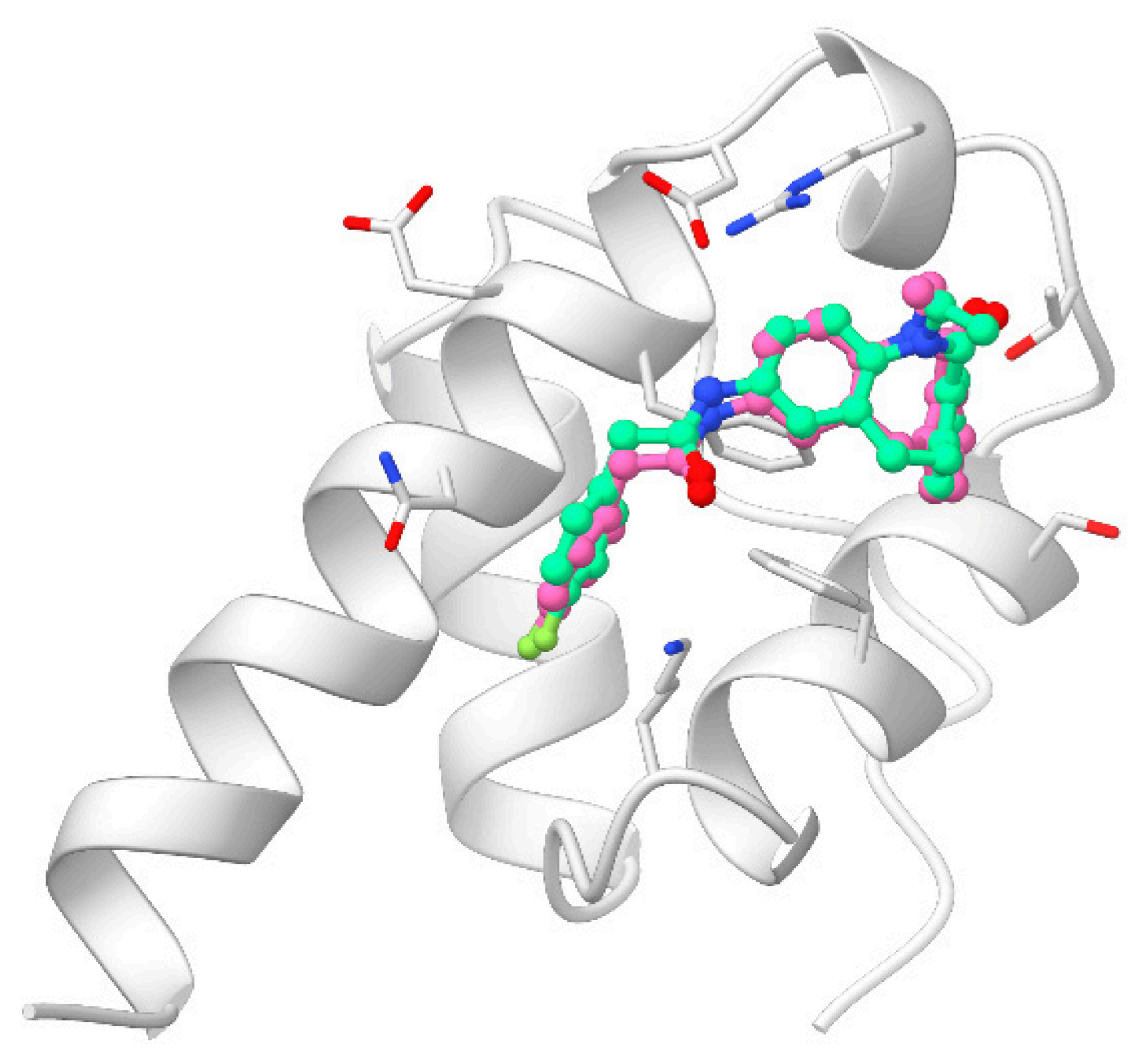
Comparison of the docking poses of compounds **7** (pink) and **9** (green) to the *Tb*PEX14 surface (silver). A high degree of similarity is observed between these two binding poses.

First, we verified whether the transition from the 6+7+6 tricyclic ring system to the 6+8+6 scaffold can yield compounds that adopt a plausible conformation to bind TbPEX14 NTD. We docked compound **9** to TbPEX14 and compared its binding pose with that of compound **7**. As shown in Figure 4, the two binding poses matched each other with high accuracy, and they agreed with the crystallography-derived binding mode of compound **4**, a sulfur analog of **7**, to TcPEX14 (PDB accession code: 7QRC, Figure 3). This finding supported the rationale for undertaking the synthesis of dibenzo[*b*,*f*]azocin-6(5*H*)-ones and benzo[*b*]pyrido[2,3-*f*]azocin-5(6*H*)-one for experimental validation of docking results in the binding assays.

### 2.2. Synthesis

**Scheme 1:**
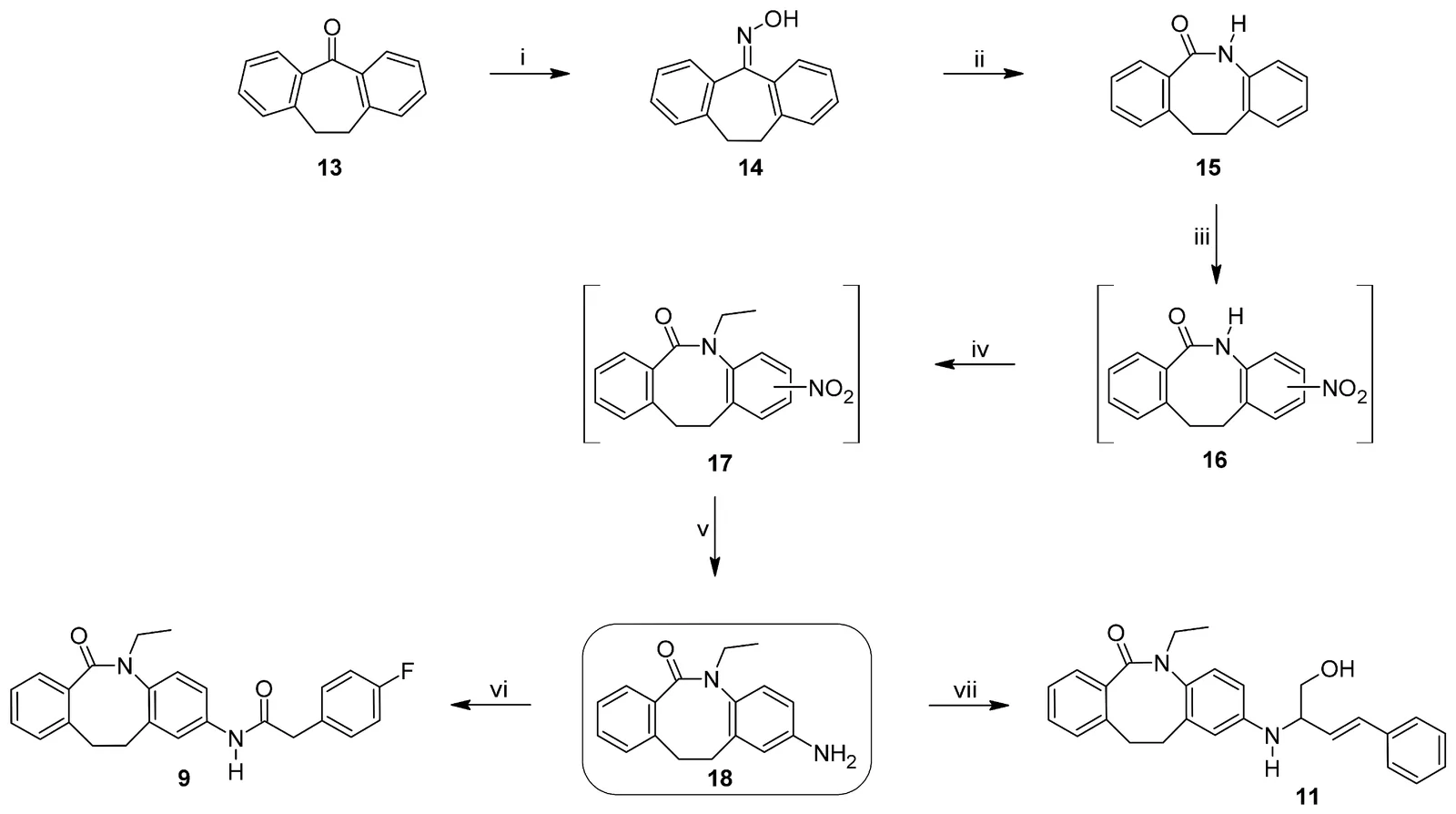
Synthesis of dibenzo[*b*,*f*]azocin-6(5*H*)-ones **9** and **11**. Reagents and conditions: i. NH_2_OH·HCl, Py, EtOH; 48%; ii. Eaton’s reagent; 97–100%; iii. HNO_3_; iv. EtBr, K_2_CO_3_, DMF; v. SnCl_2_, EtOH; 16% (overall iii–v); vi. *p*-FC_6_H_4_CH_2_CO_2_H, EDC·HCl, DMAP, DCM; 88%; vii. PhCH=CHB(OH)_2_ (*E*), glycolaldehyde dimer, MeOH; ~100%.

**Scheme 2:**
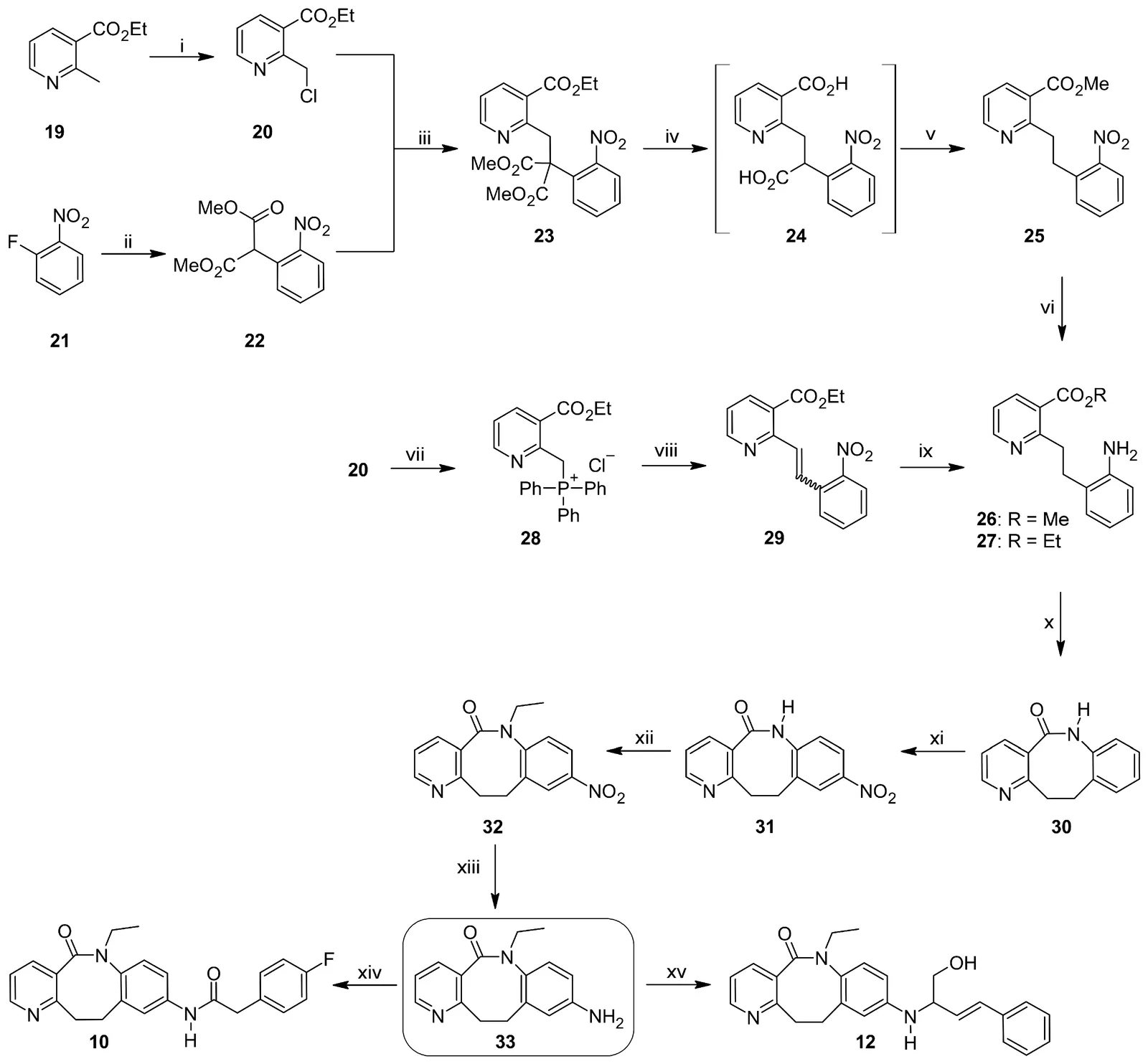
Synthesis of benzo[*b*]pyrido[2,3-*f*]azocin-5(6*H*)-ones **10** and **12**. Reagents and conditions: i. 1,3,5-Trichloroisocyanuric acid, DCM; 99%; ii. CH_2_(CO_2_Me)_2_, K_2_CO_3_, DMF; 97%; iii. K_2_CO_3_, *n*-Bu_4_N^+^Br^−^, KI, DMF; 55%; iv. 1. 2M NaOH_(aq)_, 1,4-dioxane, 2. HCl 1.4-dioxane; v. (1) K_2_CO_3_, DMF, (2) MeI; 77% (over iv–vi), 2. vi. SnCl_2_, MeOH; 98%; vii. PPh_3_, MeCN; 94%; viii. 2-Nitrobenzaldehyde, K_2_CO_3_, 1,4-dioxane; 74%; ix. HCO_2_NH_4_, Pd/C, EtOH; 82%, or 1,4-cyclohexadiene, Pd/C, EtOH, 80%; x. 1,4-dioxane, Δ, 0% (from **26** or **27**) or TBD, 1,4-dioxane; 16–25% (from **26**) or 69% (91%, from **27**), or 1. NaOH_aq_, 1,4-dioxane, 2. HCl·1.4-dioxane; 3. EDC·HCl, DMAP, DCM; 40% (from **26**), or EDC·HCl, DMAP, Et_3_N, DCM; 17% (from **26**); xi. HNO_3_, H_2_SO_4;_ 62%; xii. EtBr, K_2_CO_3_, DMF; 73%; xiii. SnCl_2_, EtOH; 100%; xiv. *p*-FC_6_H_4_CH_2_CO_2_H, EDC·HCl, Et_3_N, DMAP, DCM; 85%; xv. PhCH=CHB(OH)_2_ (*E*), glycolaldehyde dimer, MeOH; 72%.

Target compounds **9**–**12** were synthesized as shown in Scheme 1 and Scheme 2.

The 11,12-dihydrodibenzo[*b*,*f*]azocin-6(5*H*)-one tricyclic system of PEX14-PEX5 PPI inhibitors **9** and **11** was obtained by adopting literature methods [13,14,15], utilizing the Beckmann rearrangement as a key strategy to construct the 8-membered central ring of a 6+8+6 tricyclic system (Scheme 1). Thus, condensation of dibenzosuberone **13** with hydroxylamine gave oxime **14** [13], which next underwent Beckmann rearrangement [14] upon treatment with Eaton’s reagent [15]. In order to install a suitable exit vector for projecting lipophilic residues toward the Trp pocket of PEX14, lactam **15** was nitrated with nitric acid, which resulted in the formation of an inseparable mixture of regioisomeric nitroarenes **16.** The subsequent lactam deprotonation with K_2_CO_3_ and alkylation with EtBr gave a mixture of isomers of **17**, which was then reduced with SnCl_2_. The desired C-2 regioisomer of aniline **18** was isolated by column chromatography and trituration. The final products **9** and **11** were obtained from compound **18** by EDC-mediated amidation and Petasis borono–Mannich reaction [16], respectively.

**Scheme 3:**
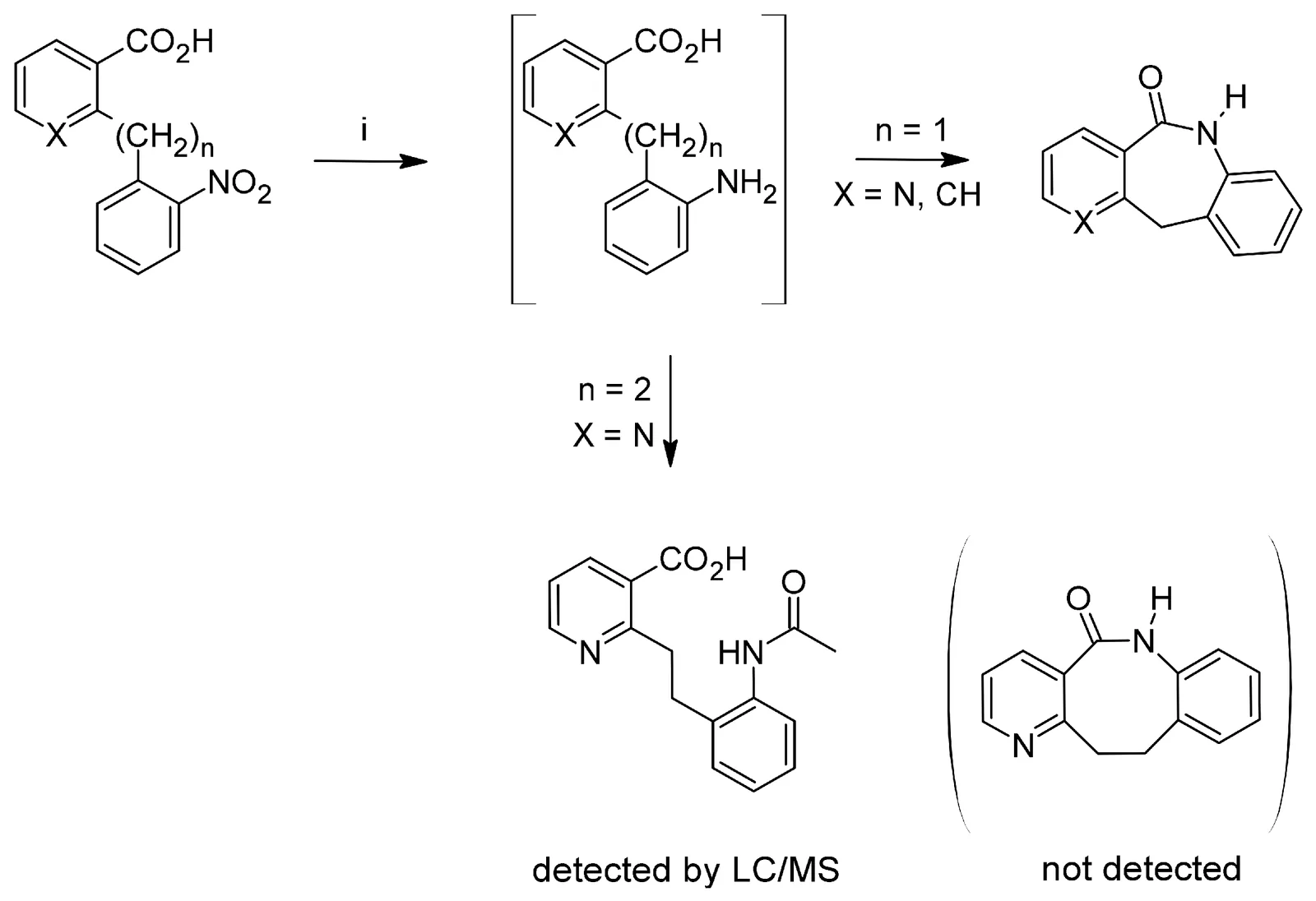
Attempted one-pot anilino ester reduction/lactamization procedure adopted from ref. [10]. Reagents and conditions: i. Fe, AcOH.

The synthesis of benzo[*b*]pyrido[2,3-*f*]azocin-5(6*H*)-one derivatives **10** and **12** proved far more challenging. We disregarded the Beckmann rearrangement-based pathway that led to the tricyclic scaffold of analogs **9** and **11** mainly for two reasons. First, using an oxime of an unsymmetrical ketone as an input implies a risk of the formation of a mixture of regioisomeric products, as described in the synthesis of a very similar, tricyclic system [17]. Second, a plausible ketone precursor for the oxime formation and the subsequent Beckmann rearrangement leading to benzo[b]pyrido[2,3-f]azocin-5(6H)-one is not readily accessible and requires 5 synthetic steps [18]. Therefore, we decided to investigate alternative strategies (Scheme 2). One of them was based on our previous experience that similar 6+7+6 heterocyclic systems can be obtained by the one-pot nitro reduction/lactamization of suitable nitro esters, triggered by treatment with Fe in AcOH [10]. We assumed that the analogous pathway can be applied for the 8-membered ring closure. First, the selective monochlorination of the heterobenzylic group in ethyl 2-methylnicotinate **19** with trichloroisocyanuric acid gave the chloride derivative **20**. In parallel, *orto*-fluoronitrobenzene **21** was subjected to nucleophilic aromatic substitution with a carbanion generated from dimethyl malonate in the presence of K_2_CO_3_, to give the C-H acid **22**. Next, C-alkylation of the thus-obtained intermediate **22** with the chloride **20** was performed. The reaction was facilitated by the addition of catalytic amounts of KI to convert the chloride **20** into a more reactive iodide, and by simultaneous application of a phase-transfer catalyst (PTC; TBAB) for the more facile deprotonation of the C-H acid **22** and achieving the higher nucleophilicity of the generated carbanion by forming a very loose pair with the counterion *n*-Bu_4_N^+^. The resulting triester **23** was hydrolyzed and mono-decarboxylated. In order to remove the second carboxylic group from the aliphatic carbon, the intermediate diacid **24** was transformed into its potassium carboxylate and decarboxylated in DMF at 50 °C, which was facilitated by the presence of the nitro group stabilizing the formation of a benzylic-type carbanion [10,19]. Finally, we attempted the one-pot reduction/lactamization of the obtained anilino ester **25** using Fe in AcOH (Scheme 3) [10]. However, no intramolecular cyclization occurred as judged by the LC/MS analysis of the crude reaction mixture, which reflects the unfavorable formation of an eight-membered ring compared to a seven-membered ring. Instead, upon prolongation of reaction time, the in situ formed NH_2_ group gradually became acetylated in the intermolecular reaction with AcOH.

**Scheme 4:**
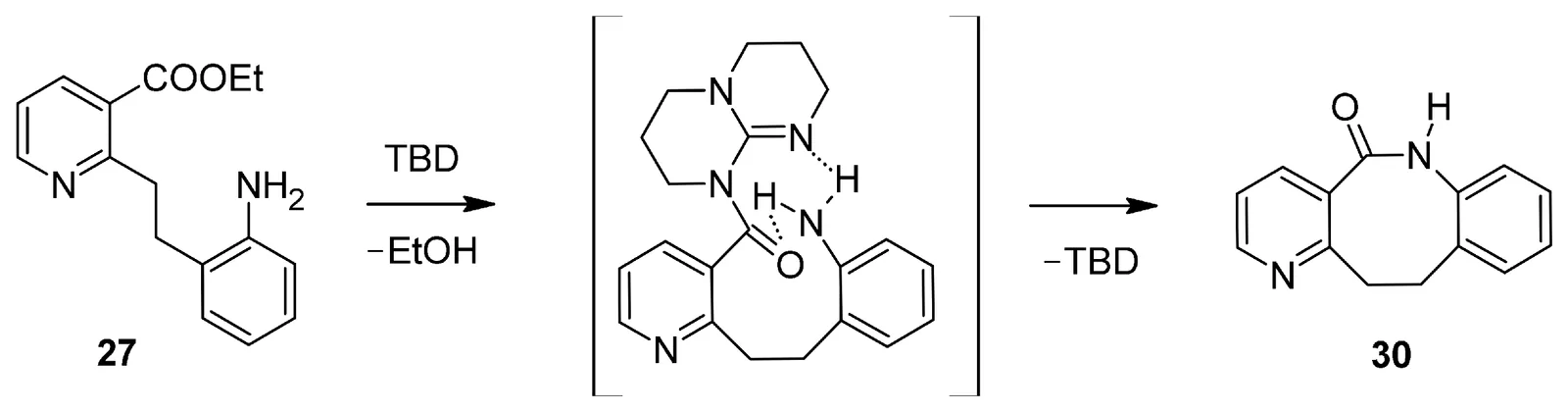
TBD-triggered intramolecular amidation of **27**.

Subsequently, we pursued a cyclization strategy based on lactamization of the suitable anilino esters and anilino acids. The nitro group reduction in compound **24** with SnCl_2_ led to the formation of anilino ester **26**. Alternatively, we obtained its ester analog **27** from chloride **20**, in a three-step synthetic sequence similar to the one described for the synthesis of 11,12-dihydrodibenz[*b*,*f*]azocin-6-one liver receptor X (LXR) modulators [20]. Thus, compound **20** was converted to the triphenylphosphonium chloride **28**, which served as a ylide precursor for the subsequent Wittig reaction with *o*-nitrobenzaldehyde. The resulting mixture of geometric isomers of nitroalkene **29** was reduced under transfer hydrogenation conditions with either ammonium formate or cyclohexadiene in the presence of a Pd/C catalyst to give the anilino ester **27**. Thermal activation of anilino esters **26** or **27** also did not result in their lactamization to the tricyclic scaffold **30**. We next attempted to adopt the literature strategy of carbodiimide-mediated activation of the carboxylate group in the intramolecular amidation [18]. Basic hydrolysis of **26** followed by EDC/DMAP coupling, both in the presence or absence of triethylamine, provided a low yield of the tricyclic 11,12-dihydrobenzo[*b*]pyrido[2,3-*f*]azocin-5(6*H*)-one **30**. Despite the high dilution of the reagents, the substrate reacted largely intermolecularly, forming oligomers, as indicated by LC/MS. Finally, we explored whether the 8-membered ring closure can be achieved through the intramolecular aminolysis triggered by 1,5,7-triazabicyclo[4.4.0]dec-5-ene (TBD) [21]. Upon addition of catalytic (30 mol%) of this base, we observed conversion of **26** to the desired lactam **30**. Because the reaction yield was unacceptably low and considerable amounts of by-products were formed, we increased the TBD load to 1 equivalent, which only slightly improved the results. Interestingly, the intramolecular cyclization of the homologous ethyl ester **27** under the same conditions provided significantly better outcomes, without the formation of open-chain oligomers. The reaction proceeded with an acceptable yield of 61% and, although the conversion of **27** was still incomplete, the unreacted substrate could be recovered by chromatography. We reasoned that TBD facilitated the generally disfavored formation of an 8-membered ring via an intermediate complex resulting from amidation of its nitrogen atom by the ester, followed by an intramolecular hydrogen bond between its second nitrogen atom and the hydrogen of the aniline moiety (Scheme 4). Similar assumptions concerning preorientation of substrates in intramolecular variants of amidation reactions catalyzed by TBD were presented in the original publication of Sabot et al. [21], reporting intermolecular ester aminolysis catalyzed by TBD under solvent-free conditions.

To create a suitable exit vector for projecting lipophilic residues toward the Trp pocket of PEX14, the tricyclic compound **30** was nitrated to give compound **31**, which was subsequently alkylated with EtBr in the presence of K_2_CO_3_ (Scheme 2). Finally, the nitro-group reduction of compound **32** using SnCl_2_ yielded aniline **33**, which was then amidated to give the target PEX14-PEX5 PPI inhibitor **10**. Compound **12** was obtained from intermediate **33** by the Petasis borono–Mannich reaction [16].

### 2.3. Biological Evaluation

Having synthesized the target tricyclic compounds **9**–**12,** we next evaluated their capability to disrupt the PEX5-TbPEX14 PPI in the AlphaScreen assay and compared the results with those obtained previously for the respective homologs **7**–**8**. The results are summarized in Table 1.

Overall, a switch from 6+7+6 to 6+8+6 ring systems did not result in marked changes in compound activity, validating our design approach. The 11,12-dihydrodibenzo[*b*,*f*]azocin-6(5*H*)-one inhibitor **9** and its homolog **7** were equipotent in the AlphaScreen assay. The 11,12-dihydrobenzo[*b*]pyrido[2,3-*f*]azocin-5(6*H*)-one derivative **10** did not disrupt the PEX5-TbPEX14 PPI at concentrations below 1.5 mM, which was in agreement with our previous observation that the compounds with the pyridine ring instead of benzene in the terminal part of the tricyclic scaffold are markedly weaker (e.g., **7** vs. **8** or 9 vs. 10). Finally, compounds **11** and **12** have a distinct (*E*)-3-(1-hydroxy-4-phenylbut-3-en-2-yl) moiety addressing the *Trp* pocket of TbPEX14. The former compound proved to be a slightly more potent PEX5-TbPEX14 PPI inhibitor than benzamide **9**. The latter derivative proved inactive, underscoring the inability of aza analogs to bind TbPEX14, which is independent of the type of moiety projecting the benzene ring toward the Trp pocket.

In our previous reports [6,7,8,9,10,11], we showed that PEX14-PEX5 PPI inhibitors display trypanocidal activity; therefore, they may constitute a new class of compounds for treating *Trypanosoma*-related diseases. In this study, we investigated whether homologation of the 6+7+6 tricyclic scaffold influences cellular activity. We tested the synthesized compounds against the *T. brucei* bloodstream form as model organisms for which glycosomal import of cytosolic enzymes is crucial for survival. In addition, we screened the TbPEX14 ligands for their cytotoxicity in HepG2 cells. We observed that compounds **9**–**11** had cellular effects similar to those of the parent 6+7+6 tricyclic homologs. Importantly, the cellular activity of compounds **9**–**12** was proportional to their potency in PEX5-TbPEX14 PPI inhibition. In line with the previous observations, EC_50_ values corresponding to the trypanocidal activities of the compounds were significantly lower than those for PEX14 inhibition. This unusual feature results from interference with glycosomal import, which causes mislocalization of enzymes that lack feedback regulation. Consequently, this leads, e.g., to uncontrolled glucose phosphorylation and ATP depletion [22]. Compounds **10** and **11** showed trypanocidal activity with single-digit IC_50_ values, whereas only the latter showed marginal cytotoxicity in HepG2 cells.

### 2.4. Computational Studies

To better understand the distinct binding properties of the two compound series, all compounds were docked into PEX14 using our previously established protocol [10]. Quantum mechanical (QM) calculations were subsequently performed on the generated docking poses, and the pose exhibiting the lowest QM binding energy was selected for each ligand. Figure S1 shows the correlation between the experimental EC_50_ values and the QM interaction energies of the selected poses. The resulting coefficient of determination indicates a reasonable agreement between the calculated energetics and the experimentally observed activities, supporting the relevance of the selected binding modes.

To further elucidate the structure–activity relationships (SARs) within this new compound family, energy decomposition and deconvolution analysis (EDDA) calculations were performed on the selected docking poses [23,24]. We first compared compounds **9** and **10**, as this pair incorporates a structural modification analogous to one previously investigated in our earlier work [10]. Consistent with those earlier findings, the introduction of a nitrogen atom generates local repulsive protein–ligand interactions that reduce the overall enthalpic stabilization of the complex (Figure S2). Energy decomposition reveals that this effect arises from the cumulative contribution of several energetic terms, including less favorable electrostatic, polarization, and dispersion interactions.

To rationalize the differences between the 6-7-6 and 6-8-6 ring systems, compounds **8** and **10** were subsequently compared (Figure S3). The calculated binding energies reproduce the experimentally observed ranking of AlphaScreen affinities. Decomposition of the interaction energies indicates that the 6-8-6 scaffold benefits from slightly more favorable electrostatic interactions. However, the dominant factors underlying the stronger binding of the 6-7-6 series are enhanced lipophilic interactions and improved shape complementarity, as reflected by the electronic density overlap (REP) term.

This observation is particularly noteworthy because dispersion interactions generally increase with molecular size, which would intuitively favor compound **10**. Instead, accommodation of the larger 8-membered ring induces a slight displacement of the ligand from the protein surface. This reduces both shape complementarity and dispersion stabilization, the latter being highly sensitive to intermolecular distance due to its inverse sixth-power dependence. Consequently, the larger ring system fails to translate its increased size into stronger attractive interactions.

Overall, the calculations indicate that compounds in the 6-7-6 series achieve superior protein–ligand shape complementarity and stronger lipophilic interactions, resulting in enhanced binding affinity. In contrast, compounds from the 6-8-6 series exhibit somewhat stronger electrostatic interactions but suffer from reduced surface complementarity. These findings suggest that, while the 6-8-6 scaffold remains an alternative to the 6-7-6 system, it does not offer significant advantages for targeting PEX14’s pocket. It also seems that other alternatives (e.g., 6-8-5) would not improve overall protein–ligand shape complementarity. The calculations strongly suggest that lipophilic dispersion forces are already dominant. Consequently, further optimization of binding potency would require addressing polar contacts with the protein residues (e.g., with residues E16, D20, R22, and K38) that may simultaneously improve the interaction with the solvent shell around the ligand–protein complex.

## 3. Materials and Methods

### 3.1. Chemistry

#### 3.1.1. General Remarks

Reagents and solvents were purchased from commercial suppliers and used without further purification. Thin-layer chromatography (TLC) was carried out on Merck (Darmstadt, Germany) TLC 60 aluminium sheets (silica gel and RP-18). Normal-phase flash column chromatography (CC) was performed using Merck silica gel 60 (particle size 0.040–0.063 mm, 230–400 mesh ASTM). LC-MS analyses were performed on an Agilent (Santa Clara, CA, USA) 1220 Infinity II Gradient LC System coupled with an Agilent LC/MSD single-quadrupole detector (column: Poroshell 120, EC-C18, 3.0 × 50 mm, 2.7 μm; gradient: water/MeCN containing 0.1% formic acid (*v*/*v*), 5–95% MeCN; UV detection at 220 and 254 nm). The high-resolution mass spectra (HRMS) were obtained using an Thermo Fisher Scientific (Waltham, MA, USA) Orbitrap Focus mass spectrometer. NMR data were recorded on Agilent 400 MHz 400-MR DD2 or Varian (Santa Clara, CA, USA) NMR System (500 MHz) instruments. NMR peaks are reported as follows: chemical shift (*δ*) in parts per million (ppm) relative to residual non-deuterated solvent as the internal standard (CHCl_3_: δ_H_ = 7.26, δ_C_ = 77. 16; DMSO: δ_H_ = 2.50, δ_C_ = 39.52 ppm; DOH: δ_H_ = 4.79), multiplicity (s = singlet, d = doublet, t = triplet, q = quartet, dd = doublet of doublets, ddd = doublet of doublets of doublets, dt = doublet of triplets, and m = multiplet and br = broad signal), coupling constant (in Hz), number of nuclei and assignment. The final compounds were ≥95% pure, as determined by NMR. The copies of the NMR spectra are provided in the Supplementary Materials (Figures S4–S45).

#### 3.1.2. Synthesis Details and Characterization Data

##### Synthesis of 10,11-Dihydro-5H-dibenzo[a,d]cyclohepten-5-one oxime (**14**)

The product was obtained according to a slightly modified literature procedure [13]. The oven-dried, thick-walled pressure glass tube was charged with dibenzosuberone **13** (12.000 g, 57.62 mmol, 1 equiv), hydroxylamine hydrochloride (12.012 g, 172.86 mmol, 3 equiv), anhydrous EtOH (60 mL), and pyridine (11.784 g, 148.98 mmol, 2.6 equiv). The resulting solution was saturated with argon, sealed with a stopper, and stirred at 100 °C for 161.5 h. The volatiles were removed, and the residue was partitioned between water (75 mL) and AcOEt (75 mL). The phases were separated, and the aqueous one was extracted with AcOEt (4 × 25 mL). All organic extracts were combined and washed with brine (50 mL). The brine was re-extracted with AcOEt (25 mL), and all organic extracts were combined, dried over Na_2_SO_4_, filtered, and evaporated. The residue was refluxed with *n*-heptane (100 mL) and cooled to rt, and the solid was filtered and washed with *n*-heptane (3 × 5 mL) to give 6.165 g (48%) of product as beige crystals.

^1^H NMR (400 MHz, CDCl_3_) δ 9.31 (br s, 1H, O*H*), 7.58 (dd, *J* = 7.7, 1.4 Hz, 1H), 7.48 (ddd, *J* = 7.4, 1.5, 0.8 Hz, 1H), 7.34–7.24 (m, 4H) overlapped by residual CHCl_3_, 7.19 (td, *J* = 7.5, 1.4 Hz, 1H), 7.15–7.11 (m, 1H), 3.23–3.08 (m, 4H, 2 × C*H*_2_); ^13^C NMR (101 MHz, CDCl_3_) δ 158.8 (*C*=NOH), 138.9, 138.5, 134.5, 133.8, 130.6, 129.5, 129.4, 128.7, 128.4, 128.4, 126.4, 125.8, 33.7 (*C*H_2_), 32.2 (*C*H_2_); LR-MS (*m*/*z*): 224 [M+H]^+^. ESI HRMS (*m*/*z*): calcd for C_15_H_14_NO^+^ [M+H]^+^: 224.1070, found: 224.1069.

##### Synthesis of 11,12-Dihydrodibenzo[b,f]azocin-6(5H)-one (**15**)

The product was obtained according to a slightly modified literature procedure [25]. The oven-dried round-bottom bulb was charged with 10,11-dihydro-5*H*-dibenzo[*a*,*d*]cyclohepten-5-one oxime **14** (5.623 g, 25.18 mmol) and Eaton’s reagent (34 mL). The suspension was stirred at 100 °C for 0.5 h, cooled down to rt, and poured into ice (160 g). The resulting suspension was warmed to rt, and the solid was filtered, washed with water (3 × 10 mL) and Et_2_O (3 × 10 mL) to give 5.749 g (~100%) of beige solid.

^1^H NMR (400 MHz, DMSO-*d*_6_) δ 9.71 (s, 1H, CON*H*), 7.20.7.16 (m, 1H), 7.15–7.01 (m, 6H), 6.98–6.94 (m, 1H), 3.25 (br s, 1H, C*H*_2_), 2.98 (br s, 1H, C*H*_2_); LR-MS (*m*/*z*): 224 [M+H]^+^, 447 [2M+H]^+^, 469 [2M+Na]^+^. The spectrum is in agreement with that previously reported in the literature [23].

##### Synthesis of 2-Amino-5-ethyl-11,12-dihydrodibenzo[b,f]azocin-6(5H)-one (**18**)

The round-bottom bulb was charged with 11,12-dihydrodibenzo[*b*,*f*]azocin-6(5H)-one **15** (5.673 g, 25.41 mmol, 1 equiv), which was dissolved in ~69% HNO_3_ (26.5 mL, 0.407 mol, 16 equiv). The solution was stirred at rt for 12 days. The precipitated solid was filtered and washed with chilled AcOH (1 × 5 mL) and Et_2_O (3 × 5 mL) to give 4.439 g of a mixture containing mainly 5-ethyl-2-nitro-11,12-dihydrodibenzo[*b*,*f*]azocin-6(5*H*)-one and 5-ethyl-3-nitro-11,12-dihydrodibenzo[*b*,*f*]azocin-6(5*H*)-one **15**. LR-MS (*m*/*z*): 269 [M+H]^+^, 559 [2M+Na]^+^. The oven-dried round-bottom bulb was charged with the above mixture containing mainly 5-ethyl-2-nitro-11,12-dihydrodibenzo[*b*,*f*]azocin-6(5*H*)-one and 5-ethyl-3-nitro-11,12-dihydrodibenzo[*b*,*f*]azocin-6(5*H*)-one **16** (4.318 g, 16.10 mmol), and anhydrous DMF (43 mL) was added. To the resulting solution, calcinated, milled K_2_CO_3_ (4.449 g, 32.2 mmol) and EtBr (4.7 mL, 64.5 mmol) were added, and the suspension was stirred at rt for 50 h. The volatiles were removed under reduced pressure, and the residue was further evaporated with toluene (2 × 20 mL). The residue was subjected to column chromatography (silica; using MeOH/DCM 0–1%) to give 3.360 g of yellowish foam, being mainly a mixture of 5-ethyl-2-nitro-11,12-dihydrodibenzo[*b*,*f*]azocin-6(5*H*)-one and 5-ethyl-3-nitro-11,12-dihydrodibenzo[*b*,*f*]azocin-6(5*H*)-one **17**. LR-MS (*m*/*z*): 297 [M+H]^+^, 615 [2M+Na]^+^. The round-bottom bulb was charged with the above mixture containing mainly 5-ethyl-2-nitro-11,12-dihydrodibenzo[*b*,*f*]azocin-6(5*H*)-one and 5-ethyl-3-nitro-11,12-dihydrodibenzo[*b*,*f*]azocin-6(5*H*)-one **17** (3.335 g, 11.29 mmol, 1 equiv), EtOH (84 mL), and anhydrous SnCl_2_ (10.703 g, 56.44 mmol, 5 equiv), and the solution was stirred at rt for 22.5 h. The volatiles were removed under reduced pressure, and the residue was dissolved in AcOEt (200 mL). To the stirred solution, a saturated aqueous NaHCO_3_ solution (100 mL) was added dropwise. The inorganic solid was filtered, and the phases were separated. The aqueous one was extracted with AcOEt (4 × 50 mL). The inorganic solid was washed with each organic extract after extraction. The solid was additionally washed with fresh AcOEt (5 × 50 mL), which was then concentrated to about 25 mL. All organic extracts were combined, washed with brine (50 mL), and the brine was re-extracted with AcOEt (25 mL). All organic extracts were combined, dried over Na_2_SO_4_, filtered, and evaporated. The crude was subjected to column chromatography (silica; using MeOH/DCM 1–1.5%). The fractions containing the desired product were combined, evaporated, triturated with Et2O (20 mL) overnight, and washed with Et2O (3 × 3 mL) to give 1.015 g of white solid. Overall yield after 3 steps (nitration, alkylation, and reduction) was 16%.

^1^H NMR (400 MHz, CDCl_3_) δ 7.19–7.15 (m, 1H), 7.10–7.02 (m, 2H), 6.91–6.87 (m, 1H), 6.77 (d, *J* = 8.3 Hz, 1H, C^4^*H*), 6.34 (dd, *J* = 8.3, 2.5 Hz, 1H, C^3^*H*), 6.30 (d, *J* = 2.5 Hz, 1H, C^1^*H*), 4.28–4.18 (m, 1H, ½ C*H*_2_CH_3_), 3.56–3.48 (m, 1H, ½ C*H*_2_CH_3_) overlapping 3.54 (br s, 2H, N*H*_2_), 3.40–3.32 (m, 1H, ½ C*H*_2_), 3.24–3.16 (m, 1H, ½ C*H*_2_), 2.91–2.82 (m, 1H, ½ C*H*_2_), 2.76–2.68 (m, 1H, ½ C*H*_2_), 1.20 (t, *J* = 7.2 Hz, 3H, C*H*_3_); ^13^C NMR (100 MHz, CDCl_3_) δ 172.3 (*C*ON), 145.9, 139.2, 138.4, 135.6, 132.8, 129.1, 128.9, 128.1, 126.6, 126.4, 116.1, 113.9, 43.9, 32.9, 31.1, 13.1 (*C*H_3_); LR-MS (m/z): 267 [M+H]^+^, 533 [2M+H]^+^, 555 [2M+Na]^+^. ESI HRMS (*m*/*z*): calcd for C_17_H_19_N_2_O^+^ [M+H]^+^: 267.1492, found: 267.1497.

##### Synthesis of N-(5-Ethyl-6-oxo-5,6,11,12-tetrahydrodibenzo[b,f]azocin-2-yl)-2-(4-fluorophenyl)acetamide (**9**)

The oven-dried round-bottom bulb was charged with 2-amino-5-ethyl-11,12-dihydrodibenzo[*b*,*f*]azocin-6(5*H*)-one **18** (75 mg, 0.28 mmol, 1 equiv), 4-fluorophenylacetic acid (65 mg, 0.42 mmol, 1.5 equiv), and anhydrous DCM (5 mL). The suspension was cooled to −15 °C; DMAP (5 mg, 0.04 mmol, 15 mol%) and EDC·HCl (81 mg, 0.42 mmol, 1.5 equiv) were added in one portion, and Et_3_N (130 μL, 0.93 mmol, 3.3 equiv) was added dropwise. The reaction was allowed to warm slowly to rt by allowing the cooling bath to warm to rt overnight. The reaction mixture was stirred overall for 23 h. The volatiles were evaporated, and the crude was subjected to column chromatography (silica; using MeOH/DCM 1–2%). The product was refluxed with *n*-heptane and washed with *n*-hexane to give the target product 113 mg (~100%) as a colorless oil.

^1^H NMR (400 MHz, CDCl_3_) δ 7.51–7.40 (m, 1H), 7.25–7.19 (m, 3H), 7.15 (dd, *J* = 7.1, 1.9 Hz, 1H), 7.11 (dd, *J* = 8.5, 2.5 Hz, 1H) overlapping 7.09–6.99 (m, 4H), 6.92 (d, *J* = 8.6 Hz, 1H), 6.87 (br d, *J* = 7.5 Hz, 1H), 4.32–4.22 (m, 1H, ½ C*H*_2_CH_3_), 3.58 (s, 2H, NCOC*H*_2_Ar) overlapping 3.58–3.48 (m, 1H, ½ C*H*_2_CH_3_), 3.39–3.30 (m, 1H, ½ C*H*_2_Ar), 3.26–3.17 (m, 1H, ½ C*H*_2_Ar), 2.91–2.75 (m, 2H, C*H*_2_Ar), 1.18 (t, *J* = 7.2 Hz, 3H, C*H*_3_); ^13^C NMR (101 MHz, CDCl_3_) δ 172.1 (*C*ON), 169.0 (*C*ON’), 162.3 (d, ^1^*J*_CF_ = 246.5 Hz), 139.2, 137.9, 137.8, 137.3, 135.4, 131.2, (d, ^3^*J*_CF_ = 8.0 Hz), 130.2 (d, ^4^*J*_CF_ = 3.4 Hz), 129.3, 129.2, 127.6, 126.7, 126.5, 121.0, 118.7, 116.1 (d, ^2^*J*_CF_ = 21.4 Hz), 44.1, 43.7, 32.9, 31.0, 13.1 (*C*H_3_); ^19^F NMR (376 MHz, CDCl_3_) δ −114.7; LR-MS (*m*/*z*): 403 [M+H]^+^, 425 [M+Na]^+^, 405 [2M+H]^+^. ESI HRMS (*m*/*z*): calcd for C_25_H_24_FN_2_O_2_^+^ [M+H]^+^: 403.1816, found: 403.1820.

##### Synthesis of (E)-5-Ethyl-2-((1-hydroxy-4-phenylbut-3-en-2-yl)amino)-11,12-dihydrodibenzo[b,f]azocin-6(5H)-one (**11**)

The round-bottom bulb was charged with 2-amino-5-ethyl-11,12-dihydrodibenzo[*b*,*f*]azocin-6(5*H*)-one **18** (75 mg, 0.28 mmol, 1 equiv), *trans*-2-phenylvinylboronic acid (63 mg, 0.42 mmol, 1.5 equiv), glycolaldehyde dimer (17 mg, 0.14 mmol, 0.5 equiv), and MeOH (2 mL). The mixture was stirred at rt for 48 h. The volatiles were evaporated, and the crude was subjected to column chromatography (silica; MeOH/DCM 0.5–1.5%). The product was precipitated as an oil from a DCM/n-hexane mixture using a rotary evaporator and washed with *n*-hexane (2 times) to give 116 mg (~100%) of a white foam.

^1^H NMR (400 MHz, CDCl_3_) δ 7.33–7.27 (m, 4H), 7.27–7.20 (m, 1H) overlapped by residual CHCl_3_, 7.18–7.13 (m, 1H), 7.11–6.95 (m, 2H), 6.92–6.74 (m, 2H), 6.51 (dd, *J* = 16.0, 9.9 Hz, 1H, C*H*=CH), 6.38–6.32 (m, 1H), 6.30–6.27 (m, 1H), 6.11–6.01 (m, 1H), 4.26–4.12 (m, 2H, ½ C*H*_2_CH_3_, N*H*), 4.04–3.96 (m, 1H, C*H*NH), 3.80–3.72 (m, 1H, ½ C*H*_2_OH), 3.57–3.58 (m, 1H, ½ C*H*_2_OH), 3.58–3.47 (m, 1H, ½ C*H*_2_CH_3_), 3.39–3.28 (m, 1H, ½ C*H*_2_Ar), 3.23–3.14 (m, 1H, ½ C*H*_2_Ar’), 2.91–2.67 (m, 2H, ½ C*H*_2_Ar, ½ C*H*_2_Ar’), 2.38 (br s, 1H, O*H*), 1.20 (t, *J* = 7.2 Hz, 3H, C*H*_3_); ^13^C NMR (101 MHz, CDCl_3_) δ [172.6, 172.5; (*C*ON)], [146.9, 146.8], [139.0, 138.9], [138.3 (x 2)], [136.5 (x 2)], [135.6 (x 2)], [132.3, 132.2], [132.1, 132.0], [129.2, 129.1], [128.9 (x 2)], [128.7 (x 2)], 128.0, 127.9, 127.8, [126.6, 126.5] overlapped by 126.5, [126.4, 126.3], 114.9, 114.5, 112.7, 112.4, [65.2 (x 2)], [57.5, 57.4], [44.0 (x 2)], [33.0, 32.9], [31.3, 31.1], [13.1 (x 2); (*C*H_3_)]; (doubled signals are due to the presence of atropisomers). LR-MS (*m*/*z*): 413 [M+H]^+^, 847 [2M+Na]^+^. ESI HRMS (*m*/*z*): calcd for C_27_H_29_N_2_O_2_^+^ [M+H]^+^: 413.2224, found: 413.2227.

##### Synthesis of Ethyl 2-(chloromethyl)pyridine-3-carboxylate (**20**)

The product was obtained according to a slightly modified literature procedure [26]. The oven-dried round-bottom bulb was charged with methyl nicotinate **19** (11.2 mL, 12.000 g, 72.64 mmol, 1 equiv) and anhydrous DCM (64 mL). Trichloroisocyanuric acid (25.325 g, 108.97 mmol, 1.5 mmol) was added, and the mixture was stirred at room temperature for 21.5 h. The solid was filtered off and washed with DCM (90 mL). The organic phase was washed with saturated aqueous NaHCO_3_ solution (3 × 20 mL) and brine (50 mL). The brine was re-extracted with DCM (20 mL). All organic extracts were combined, dried over Na_2_SO_4_, filtered, and evaporated. The crude was evaporated with cyclohexane (2 × 50 mL) to remove traces of DCM and Cl_2_, affording 14.353 g (99%) of the target product **19** as a yellowish oil.

^1^H NMR (400 MHz, CDCl_3_) δ 8.72 (dd, *J* = 4.8, 1.8 Hz, 1H), 8.28 (dd, *J* = 7.9, 1.8 Hz, 1H), 7.36 (dd, *J* = 7.9, 4.8 Hz, 1H), 5.12 (s, 2H, C*H*_2_Cl), 4.43 (q, *J* = 7.2 Hz, 2H, OC*H*_2_), 1.43 (t, *J* = 7.1 Hz, 3*H*, C*H*_3_). LR-MS (*m*/*z*): 200 [M+H]^+^, The ^1^H NMR spectrum is in agreement with previously reported [24].

##### Synthesis of Dimethyl (2-nitrophenyl)propanedioate (**22**)

The product was obtained according to a slightly modified literature procedure [27]. The thick-wall, glass pressure tube was charged with 1-fluoro-2-nitrobenzene **21** (4.015 g, 3.0 mL, 28.5 mmol, 1 equiv), dimethyl malonate (4.155 g, 3.6 mL, 31.5 mmol, 1.05 equiv), anhydrous DMF (60 mL), and K_2_CO_3_ (9.832 g, 71.1 mmol, 2.5 equiv), and the suspension was stirred at 60 °C for 20 h. The reaction mixture was diluted with Et_2_O (100 mL), and aqueous 1M HCl (~140 mL) was added to adjust the pH to 7–8. The phases were separated, and the aqueous one was extracted with Et_2_O (3 × 50 mL). Combined organic extracts were washed with water (2 × 5 mL) and brine (75 mL), dried over Na_2_SO_4_, and filtered, and the volatiles were removed under reduced pressure. The compound, after solidifying in the fridge, was triturated with chilled cyclohexane, filtered, and washed with chilled cyclohexane (2 times) to give 6.975 g (97%) of product as a yellowish, viscous solid.

^1^H NMR (400 MHz, CDCl_3_) 8.07 (d, *J* = 8.0 Hz, 1H, C^3^*H*), 7.65 (td, *J* = 7.6, 1.4 Hz, 1H, C^5^*H*), 7.55–7.49 (m, 2H, C^4^*H*, C^6^*H*), 5.33 [s, 1H, C*H*(CO_2_Me)], 3.80 (s, 6H, 2 x C*H*_3_); ^13^C NMR (100 MHz, CDCl_3_) δ 167.8 (*C*O_2_Me), 148.8 (*C*NO_2_), 133.7, 131.5, 129.5, 128.0, 125.4, 54.2, 53.3 LR-MS (*m*/*z*): 254 [M+H]^+^, 276 [M+Na]^+^, 529 [2M+Na]^+^. The spectra are in agreement with those previously reported in the literature [28].

##### Synthesis of Dimethyl {[3-(ethoxycarbonyl)pyridin-2-yl]methyl}(2-nitrophenyl)propanedioate (**23**)

An oven dried round-bottom flask equipped with a stirring bar was flushed with argon and charged with ethyl 2-(chloromethyl)nicotinate **20** (1.622 g, 8.13 mmol,1 equiv), dimethyl (2-nitrophenyl)propanedioate **22** (2.058 g, 8.13 mmol, 1 equiv), tetrabutylammonium bromide (52 mg, 0.16 mmol, 1 equiv), KI (135 mg, 0.81 mmol, 10 mol%), anhydrous DMF (20 mL), and calcinated potassium carbonate (1.685g, 12.19 mmol, 1.5 equiv). The mixture was stirred at 50 °C for 17 h, cooled to rt, diluted with Et_2_O (25 mL), and the pH was adjusted to ~7 using 1 M HCl. The phases were separated, and the aqueous one was extracted with Et_2_O (5 × 25 mL). All organic extracts were combined, washed with water (2 × 5 mL) and brine (40 mL), dried over Na_2_SO_4_, filtered, and evaporated. The crude was subjected to column chromatography (silica; MeOH/DCM 0–0.5%) to give 1.856 g (55%) of product as a beige solid.

^1^H NMR (400 MHz, CDCl_3_) 8.33 (dd, *J* = 4.7, 1.8 Hz, 1H), 8.21 (dd, *J* = 7.9, 1.8 Hz, 1H), 7.99 (dd, *J* = 7.9, 1.7 Hz, 1H), 7.39 (td, *J* = 7.6, 1.5 Hz, 1H), 7.34 (td, *J* = 7.6, 1.7 Hz, 1H), 7.20 (dd, *J* = 7.9, 1.5 Hz, 1H), 7.16 (dd, *J* = 7.9, 4.8 Hz, 1H), 4.54 (s, 2H, PyC*H*_2_), 4.40 (q, *J* = 7.1 Hz, 2H, OC*H*_2_), 3.70 (s, 6H, 2 × OC*H*_3_), 1.42 (t, *J* = 7.1 Hz, 3H, CH_2_C*H*_3_) (1.25 (s)—from grease-contamination in CHCl_3_); ^13^C NMR (100 MHz, CDCl_3_) δ 169.9 (*C*O_2_R), 166.2 (*C*O_2_R’), 157.4, 150.6, 150.2, 138.6, 132.1, 132.0, 131.3, 128.4, 125.9, 125.6, 121.5, 61.7, 61.0, 53.1, 41.6, 14.4 (CH_2_*C*H_3_) (29.8—from grease-contamination in CHCl_3_). LR-MS (*m*/*z*): 417 [M+H]^+^, 855 [2M+Na]^+^. ESI HRMS (*m*/*z*): calcd for C_20_H_21_N_2_O_8_^+^ [M+H]^+^: 417.1292, found: 417.1289.

##### Synthesis of Methyl 2-[2-(2-nitrophenyl)ethyl]pyridine-3-carboxylate (**25**)

The round-bottom flask equipped with a stirring bar was charged with dimethyl {[3-(ethoxycarbonyl)pyridin-2-yl]methyl}(2-nitrophenyl)propanedioate **23** (1.33 g, 3.19 mmol, 1 equiv), 1,4-dioxane (13.7 mL), and 2M NaOH aqueous solution (12.8 mL, 25.6 mmol, 8 equiv). The suspension was stirred at rt for 20.5 h. The volatiles were removed under reduced pressure, the bulb was cooled in an ice bath, and HCl-1,4-dioxane solution (4 M, 6.4 mL, 25.6 mmol, 8 equiv) was added dropwise. The volatiles were evaporated, and the crude 2-[2-carboxy-2-(2-nitrophenyl)ethyl]pyridine-3-carboxylic acid **24** was additionally evaporated with cyclohexane (3 × 10 mL) to remove traces of residual solvents. LR-MS (m/z): 317 [M+H]^+^; 315 [M-H^+^]^−^, 653 [2(M-H^+^)+Na^+^]^−^. Then, calcinated potassium carbonate (0.88 g, 6.39 mmol, 2 equiv) and anhydrous DMF (10 mL) were added, and the mixture was stirred at 60 °C for 23 h to give crude sodium/potassium 2-[2-(2-nitrophenyl)ethyl]pyridine-3-carboxylates (LR-MS (*m*/*z*): 273 [M+H]^+^) that were O-methylated/esterified by adding iodomethane (0.4 mL, 6.4 mmol, 2 equiv), and stirring the mixture at rt for 70.5 h. The reaction mixture was diluted with Et_2_O (15 mL) and distilled water (20 mL), and the phases were separated. The aqueous phase was extracted with Et_2_O (5 × 15 mL). The organic extracts were combined, extracted with distilled water (2 × 3 mL), and washed with brine (15 mL), and the brine phase was re-extracted with Et_2_O (15 mL). All organic extracts were combined, dried over Na_2_SO_4_, filtered, and evaporated. The crude was dissolved in DCM (10 mL), and *n*-hexane (10 mL) was added. The product was precipitated using a rotary evaporator. The solid was washed with *n*-hexane (3 × 3 mL) to give 702 mg of product **25** as a yellow solid (77%).

^1^H NMR (400 MHz, CDCl_3_) 8.66 (dd, *J* = 4.8, 1.7 Hz, 1H), 8.14 (dd, *J* = 7.9, 1.8 Hz, 1H), 7.89–7.86 (m, 1H), 7.47 (td, *J* = 7.6, 1.3 Hz, 1H), 7.38–7.35 (m, 1H), 7.34–7.29 (m, 1H), 7.22 (dd, *J* = 7.9, 4.8 Hz, 1H), 3.88 (s, 3H, OC*H*_3_), 3.58–3.53 (m, 2H, C*H*_2_), 3.36 (m, 2H, C*H*_2_); ^13^C NMR (100 MHz, CDCl_3_) δ 167.0, 161.4, 152.1, 149.7, 138.6, 136.8, 132.9, 132.2, 127.1, 125.7, 124.6, 121.3, 52.5, 37.4, 32.3. LR-MS (*m*/*z*): 287 [M+H]^+^. ESI HRMS (*m*/*z*): calcd for C_15_H_15_N_2_O_4_^+^ [M+H]^+^: 287.1026, found: 287.1018.

##### Synthesis of Methyl 2-[2-(2-aminophenyl)ethyl]pyridine-3-carboxylate (**26**)

A round-bottom flask equipped with a stirring bar was charged with methyl 2-[2-(2-aminophenyl)ethyl]pyridine-3-carboxylate **25** (0.66 g, 2.32 mmol, 1 equiv), SnCl_2_ (2.2 g, 11.6 mmol, 5 equiv), and MeOH (30 mL). The resulting suspension was stirred at rt for 21.5 h, then at 60 °C for 2 h, and the volatiles were evaporated. The crude was dissolved in AcOEt (30 mL). A saturated aqueous solution of NaHCO_3_ (25 mL) was added dropwise, and the formed organic solid was filtered. The liquid phases were separated. The aqueous phase was extracted with AcOEt (3 × 15 mL), and the filtrates from each extraction were also used to wash the aforementioned inorganic solid. The organic extracts were combined, washed with brine (15 mL), and the brine was re-extracted wıth AcOEt (15 mL). All organic extracts were combined, dried over Na_2_SO_4_, filtered, and evaporated. Then, all organic extracts were evaporated with cyclohexane (3 × 10 mL) to remove the traces of AcOEt. Overall yield: 582 mg (98%).

^1^H NMR (400 MHz, CDCl_3_) δ 8.71 (dd, *J* = 4.8, 1.8 Hz, 1H), 8.21 (dd, *J* = 7.9, 1.8 Hz, 1H), 7.24 (dd, *J* = 7.9, 4.7 Hz, 1H) overlapped by residual CHCl_3_, 7.15 (dd, *J* = 7.4, 1.6 Hz, 1H), 7.05 (td, *J* = 7.6, 1.6 Hz, 1H), 6.74–6.68 (m, 2H), 4.27 (br s, 2H, N*H*_2_), 3.92 (s, 3H, OC*H*_3_), 3.45–3.36 (m, 2H, C*H*_2_Py), 2.97–2.88 (m, 2H, C*H*_2_Ar); ^13^C NMR (101 MHz, CDCl_3_) δ 167.0, 163.0, 152.4, 145.1, 138.8, 130.4, 127.4, 125.8, 125.0, 121.3, 118.4, 115.6, 52.6, 37.4, 32.7. LR-MS (*m*/*z*): 257 [M+H]^+^. ESI HRMS (*m*/*z*): calcd for C_30_H_33_N_4_O_4_^+^ [M+H]^+^: 257.1285, found: 257.1285.

##### Synthesis of (3-Ethoxycarbonyl-pyridin-2-ylmethyl)-triphenyl-phosphonium chloride (**28**)

The oven-dried round-bottom bulb was charged with ethyl 2-(chloromethyl)pyridine-3-carboxylate **20** (14.35 g, 71.9 mmol, 1 equiv), anhydrous MeCN (72 mL), and PPh_3_ (20.74 g, 79.1 mmol, 1.1 equiv), and the mixture was stirred at rt for 5 days, and the solvent was evaporated. The residue was additionally evaporated with cyclohexane (50 mL) and portioned between water (250 mL) and Et_2_O (25 mL). The phases were separated, and the aqueous one (containing product) was washed with Et_2_O (5 × 25 mL) and evaporated to give 33.21 g (94%) of product as a brownish foam.

^1^H NMR (400 MHz, D_2_O) δ 8.46 (dd, *J* = 5.0, 1.8 Hz, 1H), 8.29–8.24 (m, 1H), 7.84–7.77 (m, 3H), 7.63–7.53 (m, 12H), 7.48–7.43 (m, 1H), 5.39 (d, ^2^*J*_PH_ = 15.1 Hz, 2H, C*H*_2_-P), 4.04 (q, *J* = 7.1 Hz, 2H, C*H*_2_O), 1.17 (t, *J* = 7.1 Hz, 3H, C*H*_2_); ^13^C NMR (101 MHz, D_2_O) δ 166.3 (*C*O_2_Et), 152.4 (d, *J*_PC_ = 2.3 Hz), 150.04 (d, *J*_PC_ = 8.8 Hz) overlapping 150.0 (d, *J*_PC_ = 8.8), 140.2 (d, *J*_PC_ = 2.4 Hz), 135.0 (d, *J*_PC_ = 3.1 Hz), 133.9 (d, ^2^*J*_PC_ = 10.2 Hz, P*Ph*), 129.8 (d, ^3^*J*_PC_ = 12.9 Hz, P*Ph*), 126.9 (d, *J*_PC_ = 5.1 Hz), 124.0 (d, *J*_PC_ = 3.2 Hz), 117.3 (d, ^1^*J*_PC_ = 87.7 Hz, *Ph*), 117.2 (d, ^1^*J*_PC_ = 88.1 Hz, *Ph*), 62.8 (O*C*H_2_). 31.3 (d, ^1^*J*_PC_ = 51.1 Hz, *C*H_2_P), 13.0 (*C*H_3_). LR-MS (*m*/*z*): 426 [M-Cl^−^]^+^. ESI HRMS (*m*/*z*): calcd for C_27_H_25_NO_2_P^+^ [M-Cl^−^]^+^: 426.1617, found: 426.1612.

##### Synthesis of Ethyl 2-[2-(2-nitrophenyl)ethenyl]pyridine-3-carboxylate (**29**)

The round-bottom bulb was charged with (3-ethoxycarbonyl-pyridin-2-ylmethyl)-triphenyl-phosphonium chloride **28** (31.217 g, 67.581 mmol, 1 equiv), 2-nitrobenzaldehyde (10.213 g, 67.58 mmol, 1 equiv), and non-anhydrous 1,4-dioxane (135 mL). After dissolution, K_2_CO_3_ (11.208 g, 20.74 mmol, 1.3 equiv) was added, and the suspension was refluxed for 6 h. The mixture was cooled to rt, an additional portion of K_2_CO_3_ (2.802 g, 20.27 mmol, 0.3 equiv) was added, and the mixture was refluxed for an additional 6 h. The volatiles were removed by evaporation, and the residue was partitioned between water (150 mL) and AcOEt (200 mL). The phases were separated, and the aqueous one was extracted with AcOEt (2 × 100 mL). All organic extracts were combined, washed with brine (100 mL), and the brine was re-extracted with AcOEt (100 mL). All organic extracts were combined, dried over Na_2_SO_4_, filtered, and evaporated. The residue was subjected to column chromatography (silica; using DCM/cyclohexane 50–100%, then AcOEt/cyclohexane 25%) to give 14.94 g (74%) of product (according to ^1^HNMR being a mixture: *E*/*Z:* ~92–93: 7–8%) as a yellowish solid.

^1^H NMR (400 MHz, CDCl_3_) δ 8.76–8.73 (m, 1H), 8.34 (d, *J* = 15.5 Hz, 1H, *H*-C=C; *E*), 8.23 (dd, *J* = 8.0, 1.8 Hz, 1H), 8.15 (d, *J* = 15.4 Hz, 1H, C=C-*H*; *E*), 7.96 (d, *J* = 8.1 Hz, 1H), 7.85 (d, *J* = 7.8 Hz, 1H), 7.64–7.59 (m, 1H), 7.44 (t, *J* = 7.7 Hz, 1H), 7.27 (dd, *J* = 8.0, 4.6 Hz, 1H) overlapped by residual CHCl_3_, 4.41 (q, *J* = 7.1 Hz, 2H, C*H*_2_), 1.41 (t, *J* = 7.1 Hz, 3H, C*H*_3_); ^13^C NMR (100 MHz, CDCl_3_) δ 166.4, 154.6, 152.5, 148.8, 138.8, 133.2, 132.9, 131.0, 130.2, 129.0, 128.8, 124.8 (x2), 122.3, 61.7 (O*C*H_2_), 14.4 (*C*H_3_). LR-MS (*m*/*z*): 299 [M+H]^+^. ESI HRMS (*m*/*z*): calcd for C_16_H_15_N_2_O_4_^+^ [M+H]^+^: 299.1026, found: 299.1029.

##### Synthesis of Ethyl 2-[2-(2-aminophenyl)ethyl]pyridine-3-carboxylate (**27**)

**Method A**

The round-bottom bulb was charged with ethyl 2-[2-(2-nitrophenyl)ethenyl]pyridine-3-carboxylate **29** (14.84 g, 49.75 mmol; 1 equiv) and EtOH (330 mL). To the stirred suspension was added Pd/C (10 wt% palladium on activated carbon; 1.588 g, 1.49 mmol, 3 mol%) and, portion-wise, ammonium formate (18.824 g, 298.50 mmol; 6 equiv). The resulting suspension was stirred at rt for 91 h and filtered through a Celite plug. The celite was washed extensively with AcOEt, and the volatiles were removed under reduced pressure. The residue was portioned between water (50 mL) and EtOAc (100 mL), and the phases were separated. The aqueous one was extracted with AcOEt (3 × 50 mL). All organic extracts were combined and washed with brine. The brine was re-extracted with AcOEt (25 mL). All organic extracts were combined, dried over Na_2_SO_4_ and evaporated. The crude was subjected to column chromatography (on silica; AcOEt/cyclohexane 25–66%). After chromatography, the product was dissolved in DCM, n-hexane was added, and the mixture was precipitated by rotary evaporation and washed with n-hexane (4 times) to give 10.026 g of product **27** as a white solid. The filtrate was evaporated and precipitated in the same way to give an additional 0.938 g of product. Overall yield: 10.964 g (82%).

**Method B**

The pressure glass bulb was charged with ethyl 2-[2-(2-nitrophenyl)ethenyl]pyridine-3-carboxylate **29** (370 mg, 1.25 mmol, 1 equiv), EtOH (25 mL), Pd/C (470 mg, 0.44 mmol, 35% mol), and 1,4-cyclohexadiene (1.18 mL). Argon was passed through this mixture for 20 min. After that, the bulb was sealed, and the reaction was stirred at 85 °C for 7.5 h. The mixture was filtered through a plug of Celite and evaporated. The crude was dissolved in DCM, *n*-hexane was added, and the product was precipitated by concentrating the mixture on a rotary evaporator. The solid was washed with *n*-hexane (3 × 2 mL) to give 270 mg (80%) of product **27** as a yellowish solid.

^1^H NMR (400 MHz, CDCl_3_) δ 8.70 (dd, *J* = 4.8, 1.8 Hz, 1H), 8.21 (dd, *J* = 7.9, 1.8 Hz, 1H), 7.24 (dd, *J* = 7.9, 4.7 Hz, 1H) overlapped by residual CHCl_3_, 7.15 (dd, *J* = 7.4, 1.6 Hz, 1H), 7.05 (td, *J* = 7.6, 1.6 Hz, 1H), 6.74–6.67 (m, 2H), 4.39 (q, *J* = 7.1 Hz, 2H, OC*H*_2_), 4.05 (br s, 2H, N*H*_2_), 3.44–3.37 (m, 2H, C*H*_2_Py), 2.96–2.88 (m, 2H, C*H*_2_Ar), 1.40 (t, *J* = 7.1 Hz, 3H, C*H*_3_); ^13^C NMR (100 MHz, CDCl_3_) δ 166.6, 163.0, 152.3, 145.1, 138.7, 130.3, 127.4, 125.9, 125.4, 121.3, 118.4, 115.6, 61.7 (O*C*H_2_), 37.4, 32.7, 14.4 (*C*H_3_). LR-MS (*m*/*z*): 271 [M+H]^+^. ESI HRMS (*m*/*z*): calcd for C_16_H_19_N_2_O_2_^+^ [M+H]^+^: 271.1441, found: 271.1438.

##### Synthesis of 11,12-Dihydropyrido[3,2-c][1]benzazocin-5(6H)-one (**30**)

**Method A**

A thick-walled pressure-resistant glass tube (25 mL) equipped with a stirring bar was flushed with argon and charged with methyl 2-[2-(2-aminophenyl)ethyl]pyridine-3-carboxylate **26** (376 mg, 1.5 mmol), TBD (61 mg, 0.4 mmol), and anhydrous 1,4-dioxane (8.5 mL). The mixture was stirred at 130 °C for 6 days. Next, MeOH (0.5 mL) was added, and the volatiles were evaporated. The crude was portioned between distilled water (10 mL) and AcOEt (10 mL), and the phases were separated. The aqueous phase was extracted with AcOEt (6 × 10 mL). The organic extracts were combined, washed with brine (10 mL), and the brine was re-extracted with AcOEt (10 mL). All organic extracts were combined, dried over Na_2_SO_4_, filtered, and evaporated. The crude was subjected to column chromatography (silica; AcOEt/cyclohexane 20–66%). The fractions, being a mixture of the unreacted substrate and the product, were combined, evaporated, and triturated with AcOEt (2 mL), and the solid was washed with AcOEt (3 × 1 mL) to give 51 mg (16%) of product **30** as a white solid.

**Method B**

The oven-dried heavy-walled pressure tube was charged with TBD (70 mg, 0.5 mmol, 1 equiv), methyl 2-[2-(2-aminophenyl)ethyl]pyridine-3-carboxylate **26** (128 mg, 0.5 mmol, 1 equiv), and anhydrous 1,4-dioxane (2.5 mL) and heated at 135 °C for 48 h. After cooling to rt, 10 mL of a 50% MeOH/DCM solution and solid NH_4_Cl (53.5 mg, 1 mmol, 2 equiv) were added, and the volatiles were removed under reduced pressure. The residue was redissolved in MeOH/DCM 50% solution, evaporated, and subjected to column chromatography (silica, MeOH/DCM 1–2%) to give 28.5 mg (25%) of product **30** as a white solid. Moreover, 14.5 mg (9%) of unreacted substrate was recovered (isolated).

**Method C**

The oven-dried pressure tube was charged with TBD (2.577 g, 18.5 mmol, 1 equiv), ethyl 2-[2-(2-aminophenyl)ethyl]pyridine-3-carboxylate **27** (5.000 g, 18.5 mmol, 1 equiv), and anhydrous 1,4-dioxane (92.5 mL), and was stirred at 135 °C for 10.5 days. The mixture was cooled and evaporated. The residue was redissolved in 1,4-dioxane, NH_4_Cl (1.979 g, 39.99 mmol, 2 equiv) was added, and volatiles were evaporated. The residue was subjected to column chromatography (silica; MeOH/DCM 1–2%). The mixed fractions (containing unreacted substrate were combined, evaporated, refluxed with DCM, filtered, and washed with chilled DCM (3 times) to give 2.038 g of product as a white solid. The filtrate was evaporated, and the residue was subjected to column chromatography (silica; MeOH/DCM 1–2.5%). Within 2-column chromatography purifications, 0.832 g of product **30** was obtained. Overall yield: 69%. Taking into account the recovered, unreacted substrate (1.214 g), the overall yield was 91%.

**Method D**

The round-bottom bulb was charged with ethyl 2-[2-(2-aminophenyl)ethyl]pyridine-3-carboxylate **27** (125 mg, 0.46 mmol, 1 equiv), 1,4-dioxane (2 mL), and 2M aquieous solution of NaOH_aq_ obtained by dissolution of 37 mg (0.92 mmol, 2 quiv) of NaOH in water (0.46 mL), and the mixture was stirred for 21 h and then at 60 °C for 3 h. The reaction mixture was concentrated to about 0.5 mL, and then 1 mL of 1,4-dioxane was added, followed by the dropwise addition of a 4 M HCl·1,4-dioxane solution (116 µL, containing 0.46 mmol, 1 equiv of HCl). The volatiles were removed under reduced pressure, and the crude was further evaporated with cyclohexane (3 × 5 mL) to remove traces of water.

Anhydrous DCM (5 mL) and DMAP (17 mg, 0.14 mmol, 30 mol%) were added, and the mixture was cooled to −15 °C. EDC · HCl (116 mg, 0.60 mmol, 1.3 equiv) was added, and the reaction mixture was allowed to warm slowly to room temperature by allowing the ice-water bath to warm to room temperature overnight. Overall, the reaction mixture was stirred for 91 h and was portioned between water (5 mL) and DCM (5 mL). The phases were separated, and the aqueous one was extracted with DCM (3 × 10 mL). All organic extracts were combined and dried over Na_2_SO_4_, filtered, and separated. The crude was subjected to column chromatography (silica; using MeOH/DCM 1–2%) to give 41 mg (40%) of product.

**Method E**

The round-bottom bulb was charged with NaOH (148 mg, 3.70 mmol) and water (1.85 mL). After dissolution and cooling to rt, ethyl 2-[2-(2-aminophenyl)ethyl]pyridine-3-carboxylate **27** (500 mg, 1.85 mmol) and 1,4-dioxane (7.4 mL) were added and the mixture was stirred at rt for 23.5 h, and then heated at 60 °C for 2 h, cooled to rt and 4M HCl · 1,4-dioxane solution (0.92 mL, containing 3.70 mmol of HCl, 2 equiv) was added. The volatiles were removed by evaporation, and the residue was further evaporated with cyclohexane (3 × 15 mL) to remove traces of water. Anhydrous DCM (18.5 mL) was added to the residue; the suspension was cooled to −15 °C, and DMAP (34 mg, 0.28 mmol, 0.15 equiv), EDC · HCl (535 mg, 2.77 mmol, 1.5 equiv), and Et_3_N (0.64 mL, 4.62 mmol, 2.5 equiv) were added. The reaction was allowed to warm slowly to rt by allowing the cooling bath to warm to rt overnight. The reaction mixture was stirred for a total of 43.5 h. The volatiles were evaporated, and the crude was subjected to column chromatography (silica; MeOH/DCM 1–2%) to give 70 mg (17%) of product **30** as a yellowish solid.

^1^H NMR (400 MHz, CDCl_3_) δ 8.44 (dd, *J* = 4.9, 1.8 Hz, 1H), 7.64 (dd, *J* = 7.7, 1.8 Hz, 1H) overlapped by 7.64 (br s, 1H, N*H*), 7.19–7.14 (m, 1H), 7.13–7.06 (m, 3H), 7.05–7.02 (m, 1H), 3.48 (br s, 2H, C*H*_2_), 3.28 (s, 2H, C*H*_2_); ^1^H NMR (400 MHz, DMSO-*d*_6_) δ 9.95 (s, 1H, N*H*), 8.37 (dd, *J* = 4.8, 1.8 Hz, 1H), 7.57 (dd, *J* = 7.7, 1.8 Hz, 1H), 7.24–7.19 (m, 1H), 7.15 (dd, *J* = 7.7, 4.8 Hz, 1H), 7.12–7.07 (m, 2H), 7.02–6.97 (m, 1H), 3,43 (br s, 1H, C*H*_2_) overlapping water from DMSO and 3.21 (br s, 2H, C*H*_2_) overlapping 3.04 (br s, 1H, C*H*_2_); ^13^C NMR (101 MHz, DMSO-*d*_6_) δ 171.2 (*C*ONH), 154.7, 149.8, 137.0, 137.0, 135.7, 131.8, 130.2, 127.2, 127.0, 126.3, 121.4, 36.7 (*C*H_2_), 28.7 (*C*H_2_). LR-MS (*m*/*z*): 225 [M+H]^+^. ESI HRMS (*m*/*z*): calcd for C_14_H_13_N_2_O^+^ [M+H]^+^: 225.1022, found: 271.1022.

##### Synthesis of 9-Nitro-11,12-dihydropyrido[3,2-c][1]benzazocin-5(6H)-one (**31**)

The round-bottom bulb was charged with 11,12-dihydropyrido[3,2-*c*][1]benzazocin-5(6*H*)-one 2.093 g **30** (9.33 mmol, 1 equiv) and ~69% HNO_3_ (4.9 mL, 74.7 mmol, 8 equiv). After dissolution, the reaction mixture was cooled to −20 °C, and 95% H_2_SO_4_ (4.2 mL, 74.7 mmol, 8 equiv) was added dropwise. The solution was stirred at −15 °C for 2 h and cautiously basified with 25% aqueous ammonia solution (50 mL). The solid was filtered, washed with water (5 × 5 mL), and dried under vacuum. The solid (2.382 g) was stirred in Et_2_O (300 mL) for 1 h at rt, filtered, and washed with Et_2_O (5 × 10 mL). Then, the solid was refluxed with AcOEt (30 mL), filtered, washed with AcOEt (3 × 5 mL), and Et_2_O (3 × 5 mL) to give 1.559 g (62%) of a light brown solid.

^1^H NMR (400 MHz, CDCl_3_) δ 8.50 (dd, *J* = 4.8, 1.8 Hz, 1H, C^2^*H*), 8.35 (s, 1H, N*H*), 8.09 (d, *J* = 2.6 Hz, 1H, C^10^*H*), 7.98 (dd, *J* = 8.6, 2.6 Hz, 1H, C^8^*H*), 7.67 (dd, *J* = 7.7, 1.8 Hz, 1H, C^4^*H*), 7.23 (d, *J* = 8.6 Hz, 1H, C^7^*H*), 7.14 (dd, *J* = 7.8, 4.8 Hz, 1H, C^3^*H*), 3.55 (t, *J* = 7.5 Hz, 2H, C*H*_2_), 3.39 (t, *J* = 7.6 Hz, 2H, C*H*_2_); ^1^H NMR (400 MHz, DMSO-*d*_6_) δ 10.14 (s, 1H, N*H*), 8.41 (dd, *J* = 4.8, 1.8 Hz, 1H, C^2^*H*), 8.21 (d, *J* = 2.7 Hz, 1H, C^10^*H*), 7.97 (dd, *J* = 8.7, 2.7 Hz, 1H, C^8^*H*), 7.62 (dd, *J* = 7.7, 1.8 Hz, 1H, C^4^*H*), 7.25 (d, *J* = 8.7 Hz, 1H, C^7^*H*), 7.19 (dd, *J* = 7.7, 4.8 Hz, 1H, C^3^*H*), 3.39 (br s, 2H, C*H*_2_), 3.26 (t, *J* = 7.4 Hz, 2H, C*H*_2_); ^13^C NMR (101 MHz, DMSO-*d*_6_) δ 170.9 (*C*ON), 154.1, 150.2, 145.7, 143.4, 138.6, 136.3, 131.2, 126.8, 125.3, 122.5, 121.7, 36.5 (*C*H_2_), 28.3 (*C*H_2_). LR-MS (*m*/*z*): 270 [M+H]^+^. ESI HRMS (*m*/*z*): calcd for C_14_H_12_N_3_O_3_^+^ [M+H]^+^: 270.0873, found: 270.0874.

##### Synthesis of 6-Ethyl-9-nitro-11,12-dihydropyrido[3,2-c][1]benzazocin-5(6H)-one (**32**)

The oven-dried bulb was charged with 9-nitro-11,12-dihydropyrido[3,2-*c*][1]benzazocin-5(6*H*)-one **31** (750 mg, 2.79 mmol, 1 equiv), calcinated, milled, anhydrous K_2_CO_3_ (770 mg, 5.57 mmol, 2 equiv), anhydrous DMF (7.5 mL), and EtBr (0.83 mL, 11.1 mmol, 4 equiv), and the suspension was stirred at rt for 18 h. The volatiles were evaporated, and the crude was portioned between water (20 mL) and DCM (20 mL). The phases were separated, and the aqueous one was extracted with DCM (5 × 20 mL). All organic extracts were combined, dried over Na_2_SO_4_, filtered, and evaporated. The residue was stirred with Et_2_O (15 mL) at rt overnight. The precipitated solid was filtered and washed with Et_2_O (3 × 3 mL) to yield 419 mg of beige crystals of product **32**. The filtrate was evaporated, and the residue was stirred with Et_2_O (5 mL). The precipitated solid was filtered and washed with chilled Et_2_O (3 × 1 mL) to give an additional 189 mg of product **32**. Overall yield: 608 mg (73%).

^1^H NMR (400 MHz, CDCl_3_) δ 8.37 (d, J = 4.9 Hz, 1H, C^2^*H*), 8.04 (d, J = 2.7 Hz, 1H, C^10^*H*), 7.98 (dd, J = 8.7, 2.0 Hz, 1H, C^8^*H*), 7.50 (dd, J = 7.7, 1.8 Hz, 1H, C^4^*H*), 7.25 (d, J = 9.4 Hz, 1H, C^7^*H*), 7.04 (dd, J = 7.8, 4.9 Hz, 1H, C^3^*H*), 4.43–4.32 (m, 1H, ½ C*H*_2_CH_3_), 3.66–3.55 (m, 2H, ½ C*H*_2_CH_3_, ½ C*H*_2_Ar), 3.41–3.24 (m, 2H, C*H*_2_Ar), 3.20–3.11 (m, 1H, ½ C*H*_2_Ar), 1.25 (t, J = 7.2 Hz, 3H, C*H*_3_); ^13^C NMR (101 MHz, CDCl_3_) δ 169.6 (*C*ON), 154.1, 150.4, 147.1, 147.0, 140.2, 135.6, 132.6, 127.6, 125.5, 123.1, 121.7, 44.7, 36.4, 29.2, 13.0 (*C*H_3_). LR-MS (*m*/*z*): 298 [M+H]^+^. ESI HRMS (*m*/*z*): calcd for C_16_H_15_N_3_O_3_^+^ [M+H]^+^: 298.1182, found: 298.1186.

##### Synthesis of 9-Amino-6-ethyl-11,12-dihydropyrido[3,2-c][1]benzazocin-5(6H)-one (**33**)

The round-bottom bulb was charged with 6-ethyl-9-nitro-11,12-dihydropyrido[3,2-*c*][1]benzazocin-5(6*H*)-one **32** (0.598 g, 2.01 mmol, 1 equiv), EtOH (30 mL), and anhydrous SnCl_2_ (1.907 g, 10.06 mmol, 5 equiv), and the solution was stirred at rt for 25 h. The volatiles were removed under reduced pressure, and the residue was dissolved in AcOEt (100 mL). A saturated aqueous solution of NaHCO_3_ (25 mL) was added dropwise to the stirred solution. The inorganic solid was filtered, and the phases were separated. The aqueous one was extracted with AcOEt (5 × 50 mL). The inorganic solid was washed with each organic extract after extraction. All the organic extracts were combined, washed with brine (50 mL), and the brine was re-extracted with AcOEt (50 mL). All organic extracts were combined, dried over Na_2_SO_4_, filtered, and evaporated. The product was precipitated from the DCM/*n*-hexane mixture using a rotary evaporator and washed with *n*-hexane to give 557 mg (~100%) of the brick-red-colored solid.

^1^H NMR (400 MHz, CDCl_3_) δ 8.34 (dd, *J* = 5.1, 1.8 Hz, 1H), 7.50 (dd, *J* = 7.7, 1.8 Hz, 1H), 7.01 (dd, *J* = 7.7, 4.9 Hz, 1H), 6.80 (d, *J* = 8.2 Hz, 1H), 6.42–6.35 (m, 2H), 4.28–4.19 (m, 1H, ½ C*H*_2_CH_3_), 3.76–3.43 (m, 4H, N*H*_2_, ½ C*H*_2_CH_3_, ½ CH_2_), 3.26–3.12 (m, 2H, C*H*_2_), 2.91–2.80 (m, 2H, ½ C*H*_2_), 1.23 (t, *J* = 7.2 Hz, 3H, C*H*_3_); ^13^C NMR (101 MHz, CDCl_3_) δ 170.1 (*C*ON), 155.3, 149.5, 146.3, 139.0, 135.0, 133.9, 132.1, 127.8, 121.4, 116.1, 114.2, 44.2, 36.4, 29.5, 12.9 (*C*H_3_). LR-MS (*m*/*z*): 268 [M+H]^+^, 557 [2M+Na]^+^. ESI HRMS (*m*/*z*): calcd for C_16_H_18_N_3_O^+^ [M+H]^+^: 268.1444 found: 268.1440.

##### Synthesis of N-(6-Ethyl-5-oxo-5,6,11,12-tetrahydrobenzo[b]pyrido[2,3-f]azocin-9-yl)-2-(4-fluorophenyl)acetamide (**10**)

The oven-dried round-bottom bulb was charged with 2-amino-5-ethyl-11,12-dihydrodibenzo[*b*,*f*]azocin-6(5*H*)-one **33** (75 mg, 0.28 mmol, 1 equiv), 4-fluorophenylacetic acid (65 mg, 0.42 mmol, 1.5 equiv), and anhydrous DCM (5 mL). The suspension was cooled down to −15 °C, and DMAP (5 mg, 0.04 mmol, 15 mol%), EDC·HCl (81 mg, 0.42 mmol, 1.5 equiv), and Et_3_N (139 μL, 0.93 mmol, 3.3 equiv) were added. The reaction was allowed to warm slowly to rt by allowing the cooling bath to warm to rt overnight. The reaction mixture was stirred overall for 18 h. The volatiles were evaporated, and the crude was subjected to column chromatography (silica; using acetone/DCM 20–25%). The product was precipitated as an oil from the mixture of DCM and *n*-hexane using a rotary evaporator, washed with *n*-hexane (2 times), and dried in vacuo to give 96 mg (85%) of product **10** as a white solid.

^1^H NMR (400 MHz, DMSO-*d*_6_) δ 10.14 (s, 1H), 8.30 (dd, J = 4.8, 1.0 Hz, 1H, N*H*), 7.49 (dd, *J* = 7.5, 1.0 Hz, 1H), 7.42 (d, *J* = 2.5 Hz, 1H), 7.38–7.27 (m, 3H), 7.16–7.03 (m, 4H), 4.20–4.09 (m, 1H, ½ C*H*_2_CH_3_), 3.57 (s, 2H, C*H*_2_CONH), 3.55–3.44 (m, 1H, ½ C*H*_2_CH_3_), 3.42–3.36 (m, 1H, ½ ArC*H*_2_) overlapped by water from DMSO, 3.19–3.04 (m, 2H, C*H*_2_), 2.96–2.86 (m, 1H, ½ C*H*_2_), 1.11 (t, *J* = 7.1 Hz, 3H, C*H*_3_). ^13^C NMR (126 MHz, DMSO) δ 169.0, 168.9, 161.1 (d, ^1^*J*_CF_ = 242.2 Hz), 154.3, 149.4, 138.6, 138.3, 135.8, 134.9, 133.1, 131.9 (d, ^4^*J*_CF_ = 3.1 Hz), 130.9 (d, ^3^*J*_CF_ = 8.1 Hz), 127.1, 121.4, 119.8, 118.3, 115.0 (d, ^2^*J*_CF_ = 21.3 Hz), 43.3, 42.2, 36.0, 28.6, 12.4. ^19^F NMR (471 MHz, DMSO-*d*_6_) δ −116.50 (tt, ^3^*J*_HF_ = 9.1; ^4^*J*_HF_ = 5.5 Hz). LR-MS (*m*/*z*): 404 [M+H]^+^, 807 [2M+H]^+^, 829 [2M+Na]^+^. ESI HRMS (*m*/*z*): calcd for C_24_H_23_FN_3_O_2_^+^ [M+H]^+^: 404.1769 found: 404.1761.

##### Synthesis of (E)-5-Ethyl-2-((1-hydroxy-4-phenylbut-3-en-2-yl)amino)-11,12-dihydrobenzo[b]pyrido[2,3-f]azocin-5(6H)-one (**12**)

The screw-capped vial was charged with 2-amino-5-ethyl-11,12-dihydrobenzo[*b*]pyrido[2,3-*f*]azocin-5(6*H*)-one **33** (75 mg, 0.28 mmol, 1 equiv), *trans*-2-phenylvinylboronic acid (63 mg, 0.42 mmol, 1.5 equiv), glycolaldehyde dimer (17 mg, 0.14 mmol, 0.5 equiv), and MeOH (1.5 mL). The mixture was stirred at rt for 22 h. The volatiles were evaporated, and the crude was subjected to column chromatography (C-18; MeCN/H_2_O 25–35%) to give 83 mg (72%) of product as a white foam.

^1^H NMR (400 MHz, CDCl_3_) δ 8.24 (d, J = 4.8 Hz, 0.5 H), 8.17 (d, J = 4.8 Hz, 0.5H), 7.47 (d, J = 7.7 Hz, 1H), 7.30–7.23 (m, 4H) overlapped by residual CHCl_3_, 7.23–7.18 (m, 1H), 6.94–6.88 (m, 1H), 6.81–6.75 (m, 1H), 6.51 (d, J = 15.7 Hz, 1H, CH=C*H*Ph), 6.38–6.30 (m, 2H), 6.05 (dd, J = 16.0, 6.0 Hz, 1H, C*H*=CHPh), 4.46–4.10 (m, 3H), 4.01–3.92 (m 1H), 3.77–3.70 (m, 1H), 3.65–3.40 (m, 3H), 3.24–2.93 (m, 3H), 2.87–2.75 (m, 1H), 1.20 (t, J = 7.2 Hz, 3H, CH_3_); ^13^C NMR (100 MHz, CDCl_3_) δ [170.5, 170.4; *C*ON], 155.2, [149.3, 149.2], [147.3 (× 2)], [138.6 (× 2)], 136.5, [135.1, 135.0], [133.9 (× 2)], [132.1 (× 2)], [131.2, 131.1], 128.6, [128.1, 128.0], [127.9, 127.8], [127.7, 127.5], [126.5 (× 2)], [121.4 (× 2)], 115.1, 114.2, 112.7, 112.1, [65.1 (× 2)], 57.4, 44.3, [36.3, 36.2], [29.6, 29.5], [12.9 (× 2), *C*H_3_]; (doubled signals are due to the presence of atropisomers); LR-MS (*m*/*z*): 414 [M+H]+, 849 [2M+Na]+. ESI HRMS (*m*/*z*): calcd for C_26_H_28_N_3_O_2_^+^ [M+H]^+^: 414.2176, found: 414.2168.

### 3.2. Protein Expression and Purification

The N-terminal domain of TbPEX14 (aa 19–84) was cloned into pETM-11 (EMBL, Heidelberg, Germany). The plasmid was transformed into *E. coli* BL21. An aliquot of 5 mL of the overnight culture was inoculated into 500 mL of the autoinduction medium [29], supplemented with 50 μg/mL kanamycin. When the cell density (OD600) reached 0.8, the temperature was lowered to 18 °C, and the cells were grown overnight. The cells were harvested by centrifugation, resuspended in lysis buffer (50 mM Tris, pH 8.0, 300 mM NaCl, 10 mM β-mercaptoethanol, 20 mM imidazole, 10 mg/mL DNAseI, 1 mM AEBSF), and then lysed by sonication. The lysate, clarified by centrifugation, was passed over a Ni-NTA agarose resin (Qiagen, Hilden, Germany) pre-equilibrated with buffer A (50 mM Tris pH 8.0, 300 mM NaCl, 10 mM β-mercaptoethanol, 20 mM imidazole), and the protein of interest was eluted with the same buffer containing 250 mM imidazole. The concentrated eluate was further purified on a Superdex 75 Hiload 16/60 column (GE Healthcare, Chicago, IL, USA) in phosphate-buffered saline (PBS).

### 3.3. AlphaScreen Assay

The AlphaScreen assay was used to derive the half-maximal effective concentration (EC_50_) values for the PEX5-TbPEX14 inhibitors, according to the published protocol [6]. A total of 3 nM N-His-PEX14 was mixed with 10 nM biotinylated PEX5-derived peptide (ALSENWAQEFLA) in PBS supplemented with 5 mg/mL of BSA and 0.01% (*v*/*v*) Tween-20. A total of 5 μg/mL of streptavidin donor beads and 5 μg/mL of nickel chelate acceptor beads (PerkinElmer) were added to the mixture. The serial dilutions of the inhibitors were prepared in DMSO and mixed, with the DMSO concentration held constant at 5% (which has been shown to have no effect on the assay readout). The competition curves were measured using a serial dilution of the inhibitor, with the concentrations of all other assay components held constant. Data were measured in quadruplicate. The inhibitor EC_50_ was calculated from Hill sigmoidal fitting, with the asymptotes fixed at the maximal assay signal (no inhibitor added) and 0, respectively. The signal was determined according to the bead manufacturer’s instructions. The data were analyzed using Origin Pro 9.0 (OriginLab, Northampton, MA, USA) [30].

### 3.4. Cellular Assays

#### 3.4.1. In Vitro Trypanocidal Activity of Compounds Against *T. b. brucei*

*T. b. brucei* bloodstream form (Lister 427, MITat 1.2) parasites were grown in an HMI-11 medium containing 10% fetal bovine serum (FBS) at 37 °C with 5% CO_2_. The anti-trypanosomal activities of the compounds were evaluated using a resazurin-based 96-well plate assay. Twofold serial dilutions of each compound (10 wells per row) were prepared in 96-well plates in HMI-11 medium (100 μL/well, quadruplicate). As controls, each row included a well without a compound and a well with medium alone. A total of 100 μL of parasite cultures (4 × 10^3^/mL) were inoculated into all wells, except the well with medium alone. The final concentration of parasites was 2 × 10^3^/mL. The plates were incubated for 66 h. Resazurin (25 μL of 0.1 mg/mL in Hank’s Balanced Salt Solution) was added to all wells, and the plates were further incubated until the 72 h time point. Reduction of resazurin by living cells was quantified by measuring the fluorescence with a Synergy H1 (Agilent) microplate reader (excitation 530 nm, emission 585 nm). After subtracting the background fluorescence of the well with media alone, percent survival values were calculated by setting the fluorescence of the wells without compound to ‘100% survival’. Nonlinear regression graphs were plotted in GraphPad Software (Boston, MA, USA) GraphPad Prism 10 [31] to yield sigmoidal dose–response curves, and half-maximal effective concentration (EC_50_) values were determined.

#### 3.4.2. Cytotoxicity of Compounds Against HepG2 Cells

HepG2 (Hepatocyte) cells were seeded in 96-well plates (5000 cells/well in rows B–H) and grown overnight at 37 °C in a humidified incubator with 5% CO_2_. Compounds were tested in triplicate from 100 to 3.125 μM (twofold serial dilutions, from row H to row C). Row A contained medium alone and served as a negative control. Row B contained cells alone without inhibitors and served as a positive control. Hygromycin B (InvivoGen, San Diego, CA, USA) was used as a positive control for cytotoxicity. After incubation for 66 h, 25 μL of 0.1 mg/mL resazurin (dissolved in Hank’s Balanced Salt Solution, HBSS; Sigma) was added to all wells. Plates were incubated for an additional 6 h. Fluorescence was measured, and the data were processed as described above for the *T. b. brucei* cytotoxicity assay.

### 3.5. In Silico Studies

The computational study followed closely the protocols we previously reported [10]. In short, we used the previously prepared PEX14 structure from the PDB-ID 7qrc [9] for compound docking using Schrödinger (New York, NY, USA) Maestro pipeline [32]. Crystallization waters and other components were removed before docking. A total of 15 poses were generated per compound, and we retained the pose with the best QM binding score for each compound. Afterward, energy decomposition and deconvolution analysis (EDDA [24]) calculations were performed on each model, as available from ULYSSES [33]. For additional calculation details, we refer the reader to the previous report [10]. Molecular representations were performed using ChimeraX (University of California, San Francisco, CA, USA) [34]. Quantum mechanical binding energies were correlated with the AlphaScreen EC_50_ (its natural logarithm) to assess the relationship between model information and AlphaScreen activity. We observed that compound **9** is an outlier in the series by applying the jackknife method.

## 4. Conclusions

For a few decades, targeting PPIs with small molecules has been considered an attractive strategy in drug development. However, because of the structural characteristics of the interfaces through which proteins bind, this approach has proven extremely difficult to implement. PEX14-PEX5 PPI poses challenges typical of this target class. On the other hand, our previous structure-based drug discovery efforts have led to several inhibitor classes. One of the key findings on how this PPI can be disrupted by small molecules was the development of 6+7+6 tricyclic scaffolds **4**–**8** with a unique binding mode to the hydrophobic PPI pockets of PEX14 NTD and to the diaromatic Phe–Phe motif that separates them. In this report, we have extended this chemotype, adding more flexibility to the central scaffold by homologation with a -CH_2_- unit. The challenging chemistry of 8-membered ring formation led us to the dibenzo[*b*,*f*]azocin-6(5*H*)-one and benzo[*b*]pyrido[2,3-*f*]azocin-5(6*H*)-one scaffolds. As expected, the synthesized prototype derivatives **9**, **10**, and **11** of those 6+8+6 tricyclic derivatives did not differ markedly from the previously developed 6+7+6 inhibitors in disrupting the TbPEX14-PEX5 complex formation nor in the cellular activities against *Trypanosoma brucei*, thus offering a new, alternative scaffold for targeting this PPI. We believe that the presented findings not only constitute an interesting starting point for the development of new antiparasitic compounds but can also be employed in the design of inhibitors of other PPIs mediated by a similar topology of hydrophobic binding pockets to those observed in the PEX14-PEX5 complex.

## Figures and Tables

**Table 1:** Disruption of the PEX5-PEX14 PPI and cellular activity of compounds **7**–**12**.

7–8:	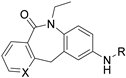		9–12:	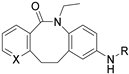	
#	-X-	R	TbPEX14 EC_50_ [µM] ^a^	*T. brucei* EC_50_ [µM] ^b^	*HepG*2 EC_50_ [µM] ^b^
**7** ^c^	CH	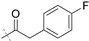	95	7.2 (6.5–7.9)	>100
**8** ^c^	N	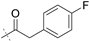	301	15 (12–19)	>50
**9**	CH	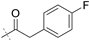	119	8.4 (7.6–9.3)	>100
**10**	N	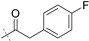	>1000	38.0 (33.9–42.4)	>100
**11**	CH	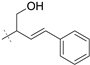	61	9.3 (8.1–10.7)	53.6 (47.4–59.6)
**12**	N	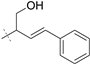	>1000	27.8 (19.6–36.3)	>100

^a^ EC_50_ values were calculated as a Hill curve fit to a 12-point titration (*n* = 4), with SD mostly within 20%. ^b^ EC_50_ values are shown as the mean (*n* = 4). Values in parentheses are 95% confidence intervals. ^c^ Reference compounds, data from ref [10].

## Data Availability

The original contributions presented in this study are included in the article/Supplementary Materials. Further inquiries can be directed to the corresponding authors.

## Supplementary Materials

The following supporting information can be downloaded at: https://www.mdpi.com/article/10.3390/ijms27187998/s1.
