# Supplementary material for: Dibenzo[b,f]azocin-6(5H)-ones and Benzo[b]pyrido[2,3-f]azocin-5(6H)-ones: Useful Scaffolds for Development of Protein–Protein Interaction Inhibitors
Source: Int J Mol Sci. 2026 Sep 8;27(18):7998. doi: 10.3390/ijms27187998 (PMC13606207; doi:10.3390/ijms27187998)
Supplement: Supplementary file 1 [file ijms-27-07998-s001.zip › ijms-4521384-supplementary.pdf]

Dibenzo[*b*,*f*]azocin-6(5*H*)-ones and Benzo[*b*]pyrido[2,3-*f*]azocin-5(6*H*)-ones as Useful Scaffolds for Development of Protein-Protein Interaction Inhibitors

Michał Nowacki [1,*], Filipe Menezes [2], Başak Dağdeviren [1], Federico Ballabio [2], Valeria Napolitano [2], Chethan K. Krishna [3], Vishal C. Kalel [3], Ralf Erdmann [3], Michael Sattler [2,4], Grzegorz M. Popowicz [2,4] and Maciej Dawidowski [1,*]

[1] Department of Drug Technology and Pharmaceutical Biotechnology, Medical University of Warsaw, Banacha 1, 02-097 Warszawa, Poland
[2] Institute of Structural Biology, Molecular Targets and Therapeutics Center, Helmholtz Zentrum München, Ingolstädter Landstraße 1, 85764 Neuherberg, Germany
[3] Institute of Biochemistry and Pathobiochemistry, Department of Systems Biochemistry, Faculty of Medicine, Ruhr-University Bochum, 44780 Bochum, Germany
[4] Bavarian NMR Center, Department of Bioscience, TUM School of Natural Sciences, Technical University of Munich, Lichtenbergstrasse 4, 85747 Garching, Germany
* Correspondence: michal.nowacki@wum.edu.pl (M.N.); maciej.dawidowski@wum.edu.pl (M.D.)

S1. Computational details

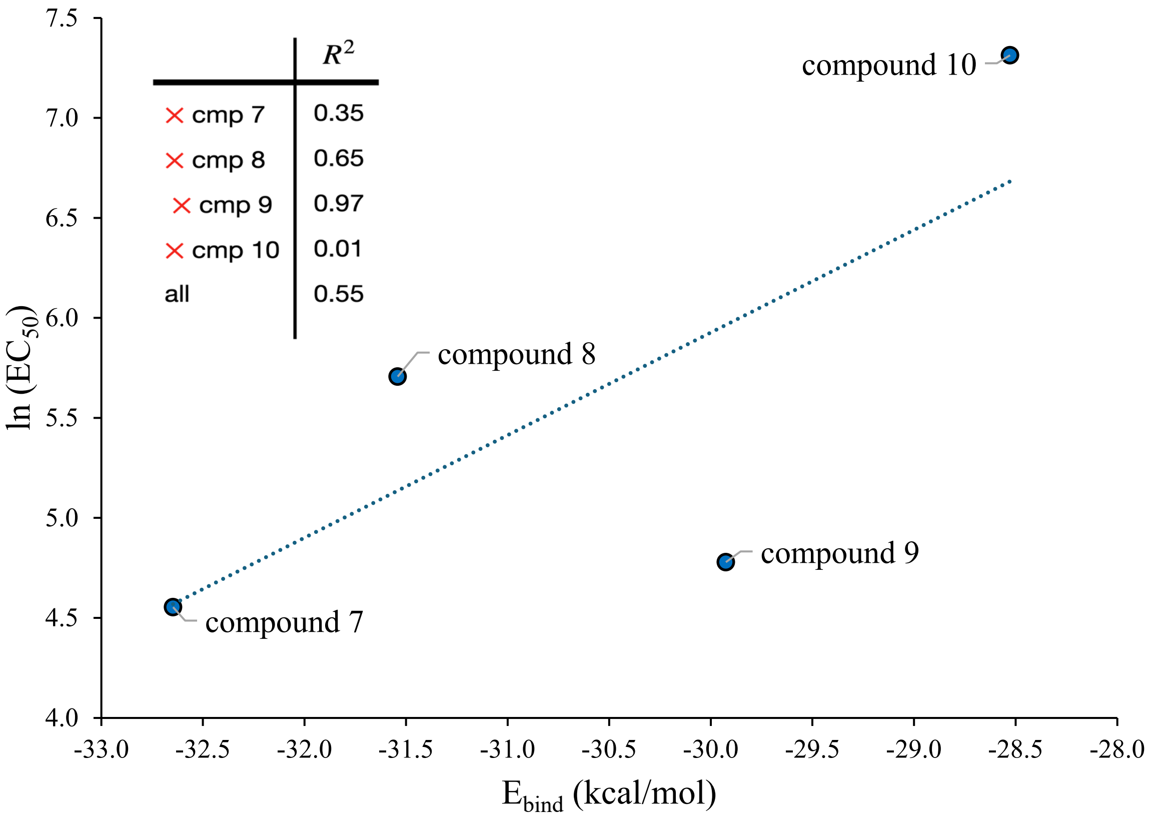

Figure S1: Correlation plot between the experimental EC_{50} (logarithm) and the QM calculated binding energies. Coefficients of determination calculated when all data points are included and when one is excluded.

Figure S1 shows the correlation between the experimental EC_{50} values and the QM interaction energies calculated for the selected binding poses of each ligand bound to PEX14. For compounds **7** and **8**, the previously selected binding poses were retained in this analysis to ensure consistency with our earlier work.[1] For completeness, additional binding poses for these two compounds were also explored, as they had the potential to yield lower QM binding energies toward Tb-PEX14; however, no improved poses were identified.

Overall, a coefficient of determination (R^2) of 0.55 was observed between the experimental and computational data. This moderate correlation appears to be largely influenced by the outlier behaviour of compound **9**, which is likely associated with a low-quality binding pose. This conclusion is supported by a jackknife robustness analysis, in which the coefficients of determination were recalculated while systematically omitting one observation at a time. Exclusion of compound **9** resulted in a marked increase in the R^2 value.

Taken together, these results indicate that the QM analysis and the underlying binding poses rationalize the experimental binding data to a good extent. To further expand our understanding of this new class of compounds, Energy Decomposition and Deconvolution Analysis (EDDA) was subsequently performed on the protein–ligand complexes. For an overview of the EDDA methodology, the reader is referred to the literature.[1-3]

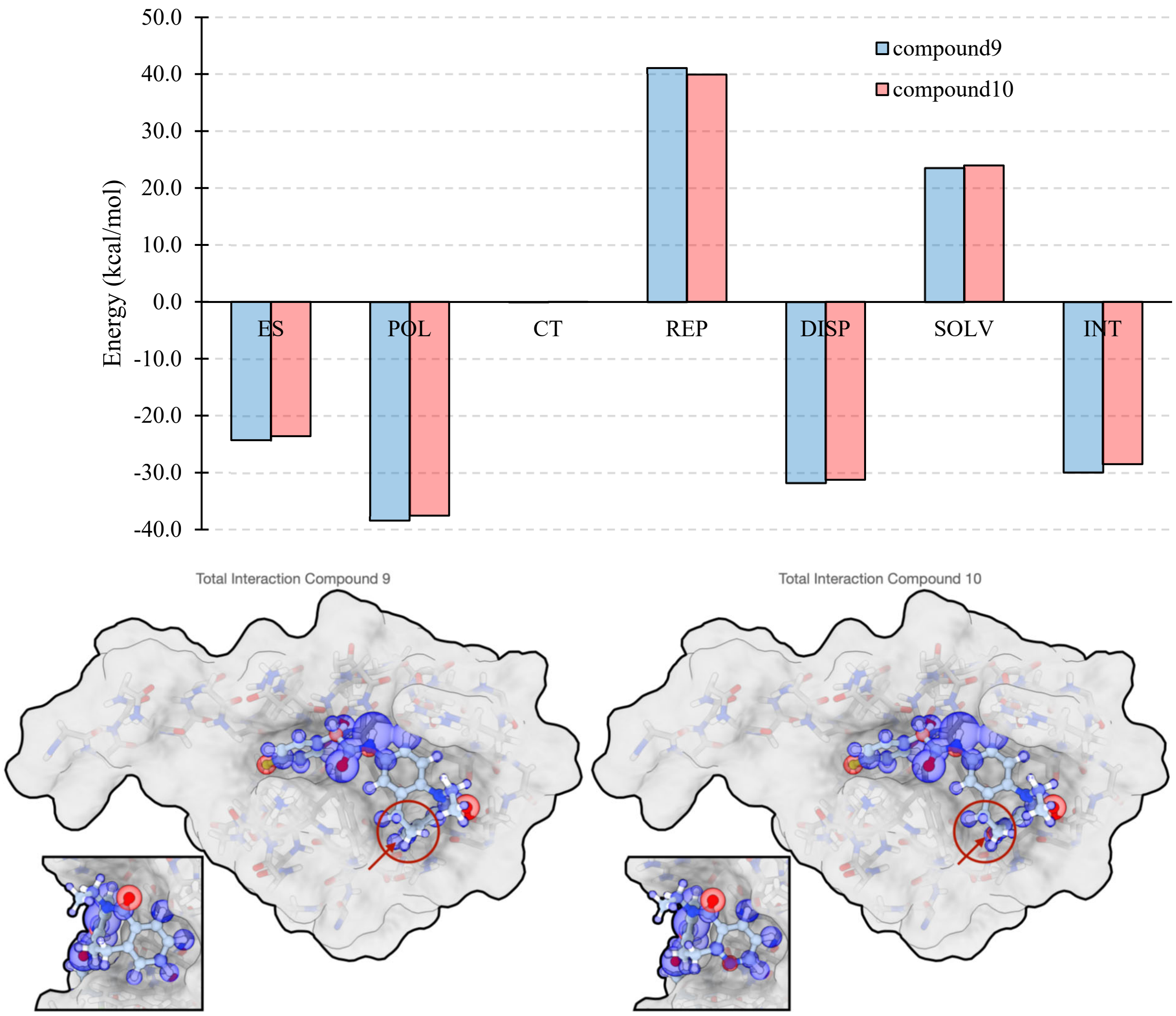

Figure S2. Energy decomposition and deconvolution analysis (EDDA) data comparing compounds **9** and **10**. Upper block, all the energy contributions calculated. Lower, total interaction maps comparing the two compounds.

Figure S2 presents the EDDA total interaction maps for compounds **9** and **10**. Overall, the interaction patterns are highly similar for both compounds and differ mainly in the region surrounding the distinct functional group, namely the CH or N atom within one of the six-membered rings (highlighted by the red circle and arrow; see also the corresponding zoomed representations of this region in the compounds).

As illustrated in the interaction maps, replacement of the aromatic CH group by a nitrogen atom introduces repulsive protein–ligand interactions, whereas such repulsive interactions are absent in the case of compound 10. The energetic distribution of interactions shown in Figure S2 further indicates that the observed difference in binding energy does not arise from a single dominant energetic contribution. Instead, it reflects the cumulative effect of several interaction components, including electrostatics, polarisation, and dispersion, together with smaller solvation penalties.

Interestingly, repulsion is slightly larger for compound **9**, a difference attributed to the replacement of the nitrogen atom by a CH group in the aromatic ring, which likely reflects a modest increase in protein-ligand shape complementarity. The relative magnitudes of the individual energetic contributions are also consistent

with our previous analysis of compounds **7** and **8**, with values of approximately ΔES ≈ −0.72 kcal/mol, ΔPOL ≈ −0.85 kcal/mol, ΔDISP ≈ −0.52 kcal/mol, and ΔINT ≈ −1.40 kcal/mol for the compound **9** and **10** pair.

Overall, these findings are in close agreement with our previous analyses and support the conclusion that introducing electron-rich atoms into the first six-membered ring of this compound series is detrimental to binding, primarily due to local repulsive effects.

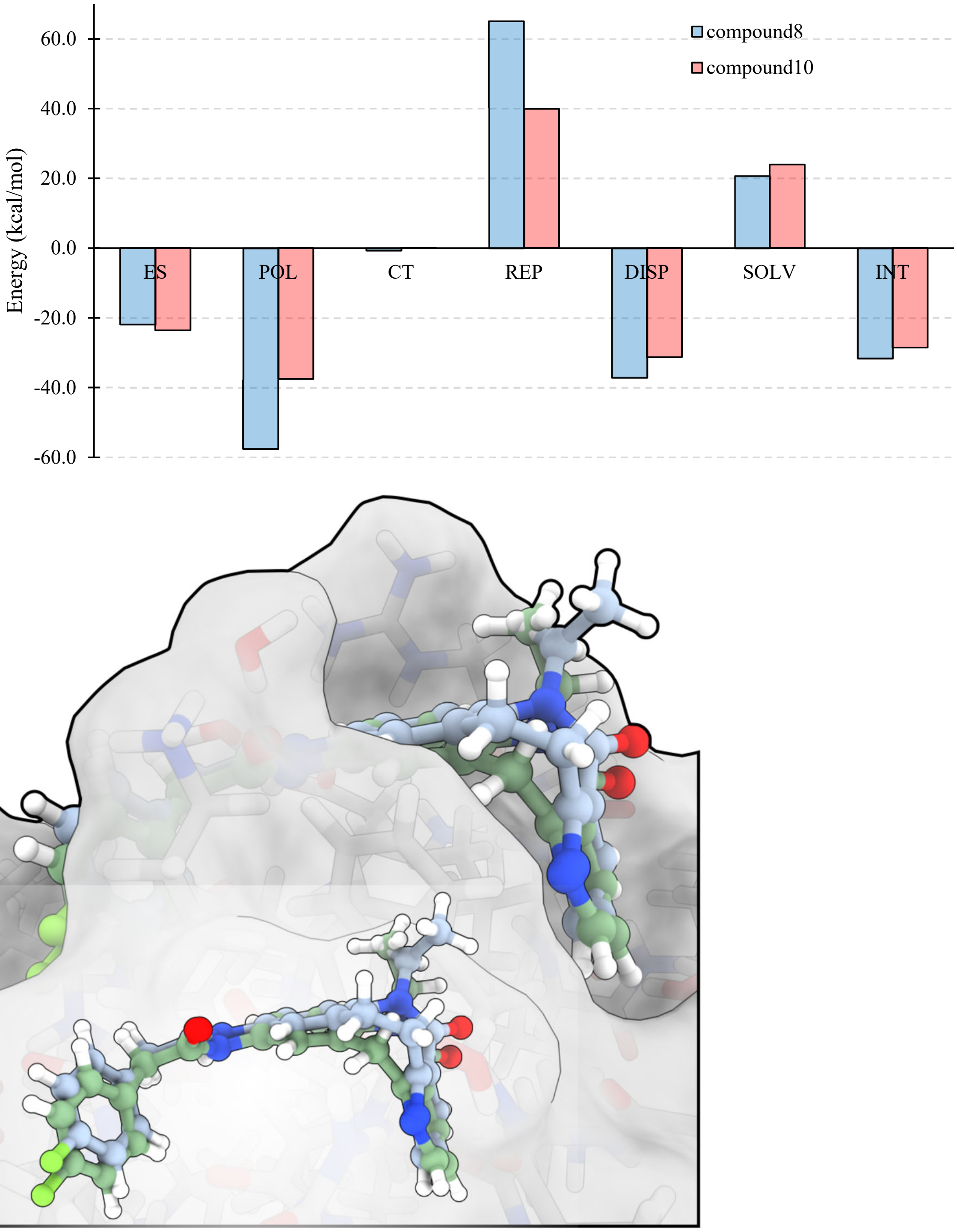

Figure S3. Energy decomposition and deconvolution analysis (EDDA) data comparing compounds **8** and **10**. Upper block, all the energy contributions calculated. Lower block, differences in binding poses.

Next, we performed a quantum mechanical analysis of compounds **8** and **10** to better understand the differences in binding between the 7- and 8-membered ring compound families. These two compounds were selected because they align more closely with the previously established correlation curve. Figure S3 presents the energy decomposition data for both compounds bound to *Tb*PEX14.

The EDDA analysis reveals that compound **8** displays a binding energy approximately 3.0 kcal/mol lower than that of compound **10**. On its own, this relative binding energy difference suggests that non-negligible entropy contributions may influence the observed results and may partially explain why compound **9** behaves as an outlier in the correlation plot shown in Figure S1.

Although both compounds **8** and **10** contain a nitrogen atom in the leftmost six-membered ring, electrostatic interactions are more favorable in the case of compound **10**. One possible explanation is that the increased flexibility associated with the larger ring system allows compound **10** to adopt conformations that better accommodate the Tb-PEX14 binding pocket, thereby enhancing electrostatic stabilization. However, this interpretation is not consistent with the observation that the electronic density overlap energy (REP in the EDDA plot), which reflects protein-ligand shape complementarity, is significantly larger for compound **8** than for compound **10**.

In principle, increased ligand flexibility is expected to facilitate adaptation to the protein pocket and maximize shape complementarity. Consequently, these results suggest that the additional flexibility gained when moving from the 6-7-6 scaffold to the 6-8-6 scaffold is accompanied by the need to accommodate an extra methylene group, which is not necessarily advantageous for binding. In other words, the 6-8-6 compounds may either be excessively flexible for optimal binding or display an imbalance in flexibility.

This interpretation is further supported by Figure S3, where the 6-8-6 compounds exhibit a poorer fit to the Tb-PEX14 pocket surface, primarily due to the increased surface extension associated with the central 8-membered ring. The need to accommodate this larger ring along the protein surface further destabilizes additional interactions. For example, it promotes a partial detachment of the fluorophenyl group as it enters the Trp pocket and also alters the interactions established between the central lactam moiety and Tb-PEX14.

Collectively, these results indicate that compounds **7** and **8** bind more favorably to Tb-PEX14 than compounds **9** and **10** because they achieve superior shape complementarity with the protein pocket, whereas the additional flexibility introduced by the 8-membered ring is detrimental to binding.

References

[1] Nowacki, M.; Menezes, F.; Pykacz, E.; Popiołek, M.; Napolitano, V.; Krishna, C.K.; Kalel, V.C.; Erdmann, R.; Fröhlich, T.; Plettenburg, O.; et al. Quantum mechanics-driven structure-activity relationship study of PEX5-PEX14 protein-protein interaction inhibitors based on a dibenzo[b,e]azepin-6(6H)-one scaffold. *Eur. J. Med. Chem.* **2025**, *298*, 117979.

[2] Góral, I.; Wichur, T.; Sługocka, E.; Grygier, P.; Głuch-Lutwin, M.; Mordyl, B.; Honkisz-Orzechowska, E.; Szałaj, N.; Godyń, J.; Panek, D.; et al. Exploring Novel GSK-3β Inhibitors for Anti-Neuroinflammatory and Neuroprotective Effects: Synthesis, Crystallography, Computational Analysis, and Biological Evaluation. *ACS Chem. Neurosci.* **2024**, *15*, 3181–3201.

[3] Grygier, P.; Pustelny, K.; Nowak, J.; Golik, P.; Popowicz, G.M.; Plettenburg, O.; Dubin, G.; Menezes, F.; Czarna, A. Silmitasertib (CX-4945), a Clinically Used CK2-Kinase Inhibitor with Additional Effects on GSK3β and DYRK1A Kinases: A Structural Perspective. *J. Med. Chem.* **2023**, *66*, 4009–4024.

S2. NMR spectra of the synthesized compounds

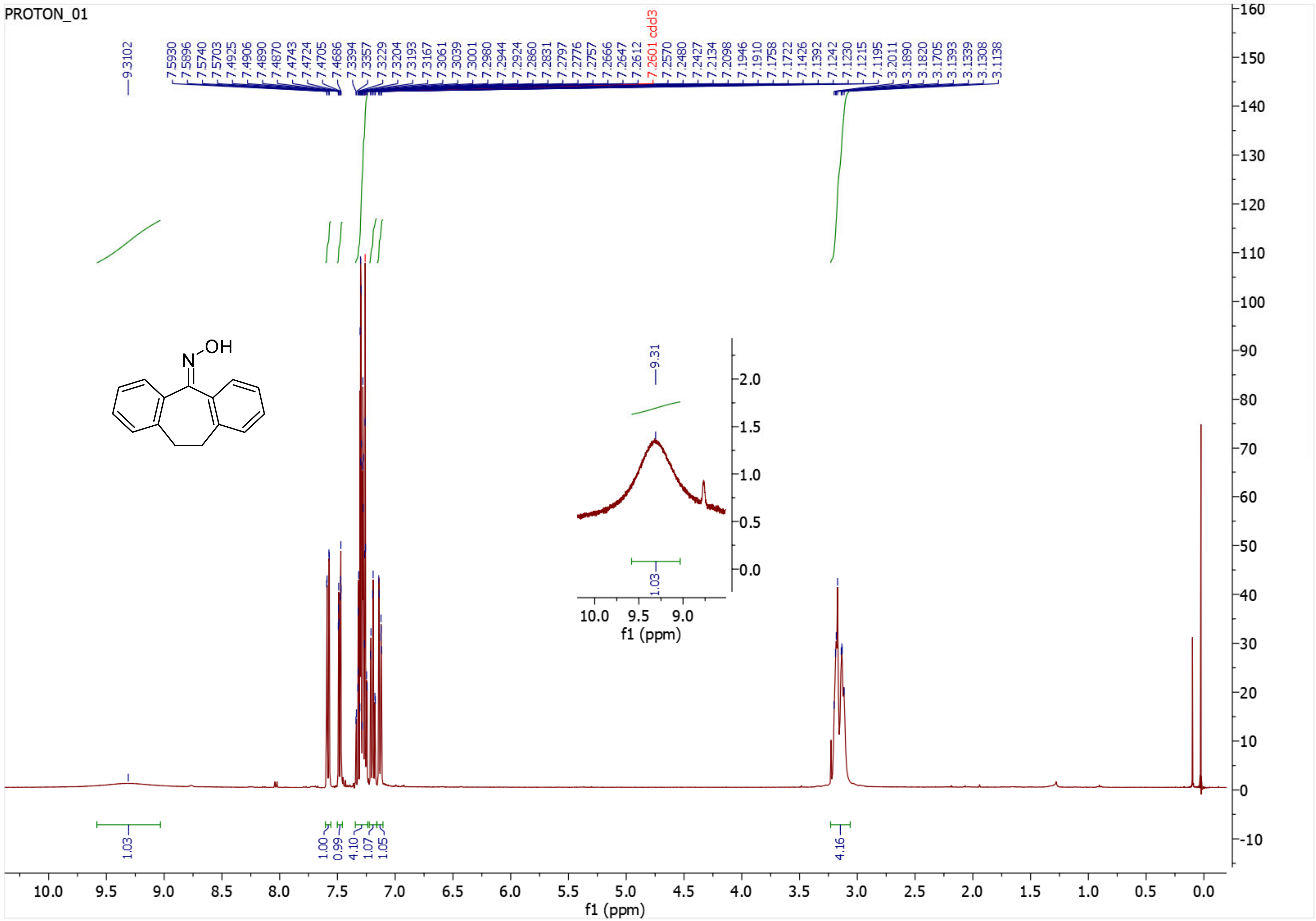

Figure S4. ^{1}H NMR spectrum ($CDCl_3$) of 10,11-dihydro-5*H*-dibenzo[*a*,*d*]cyclohepten-5-one oxime (**14**)

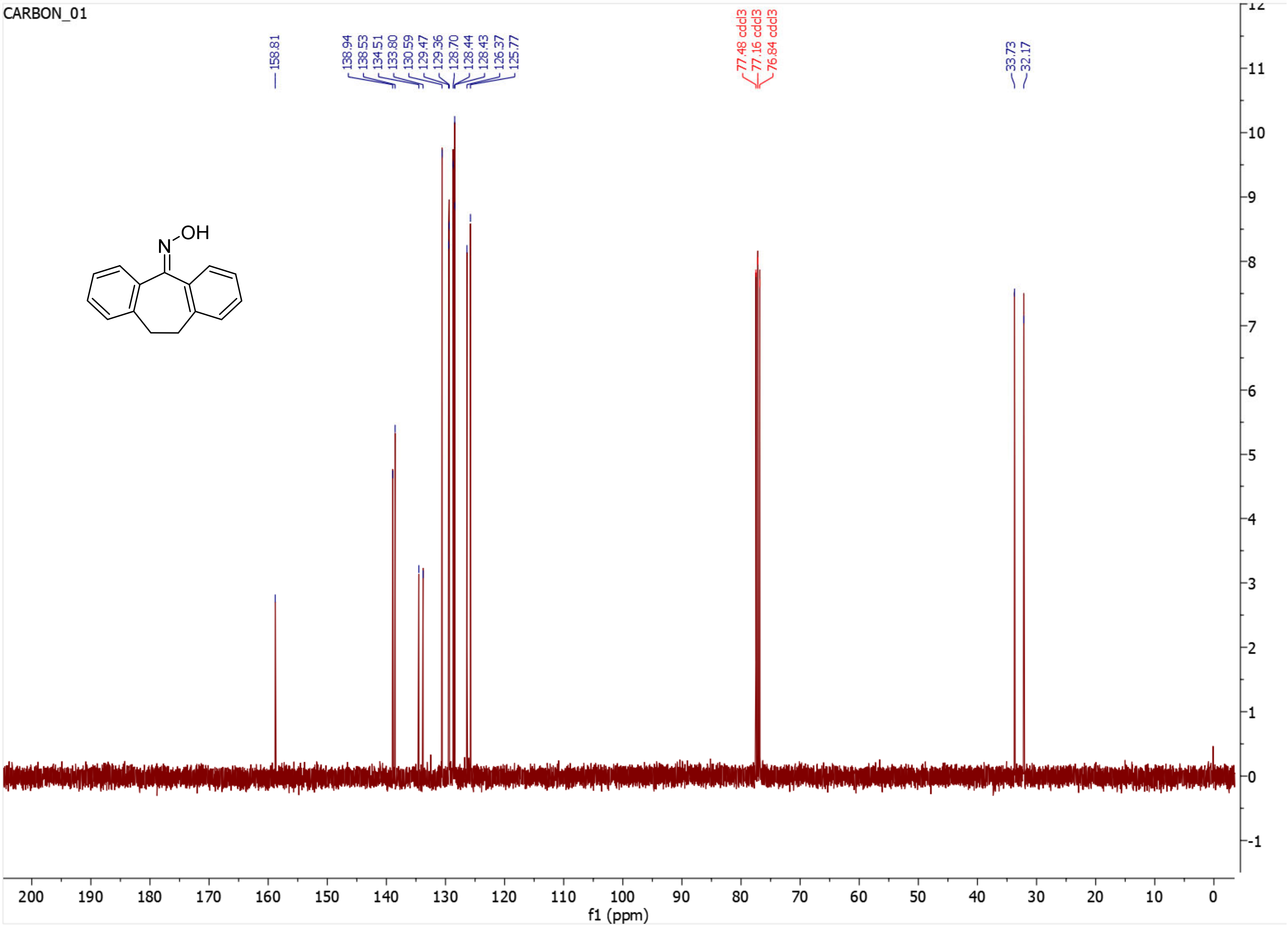

Figure S5. ^{13}C NMR spectrum ($CDCl_3$) of 10,11-dihydro-5*H*-dibenzo[*a*,*d*]cyclohepten-5-one oxime (**14**)

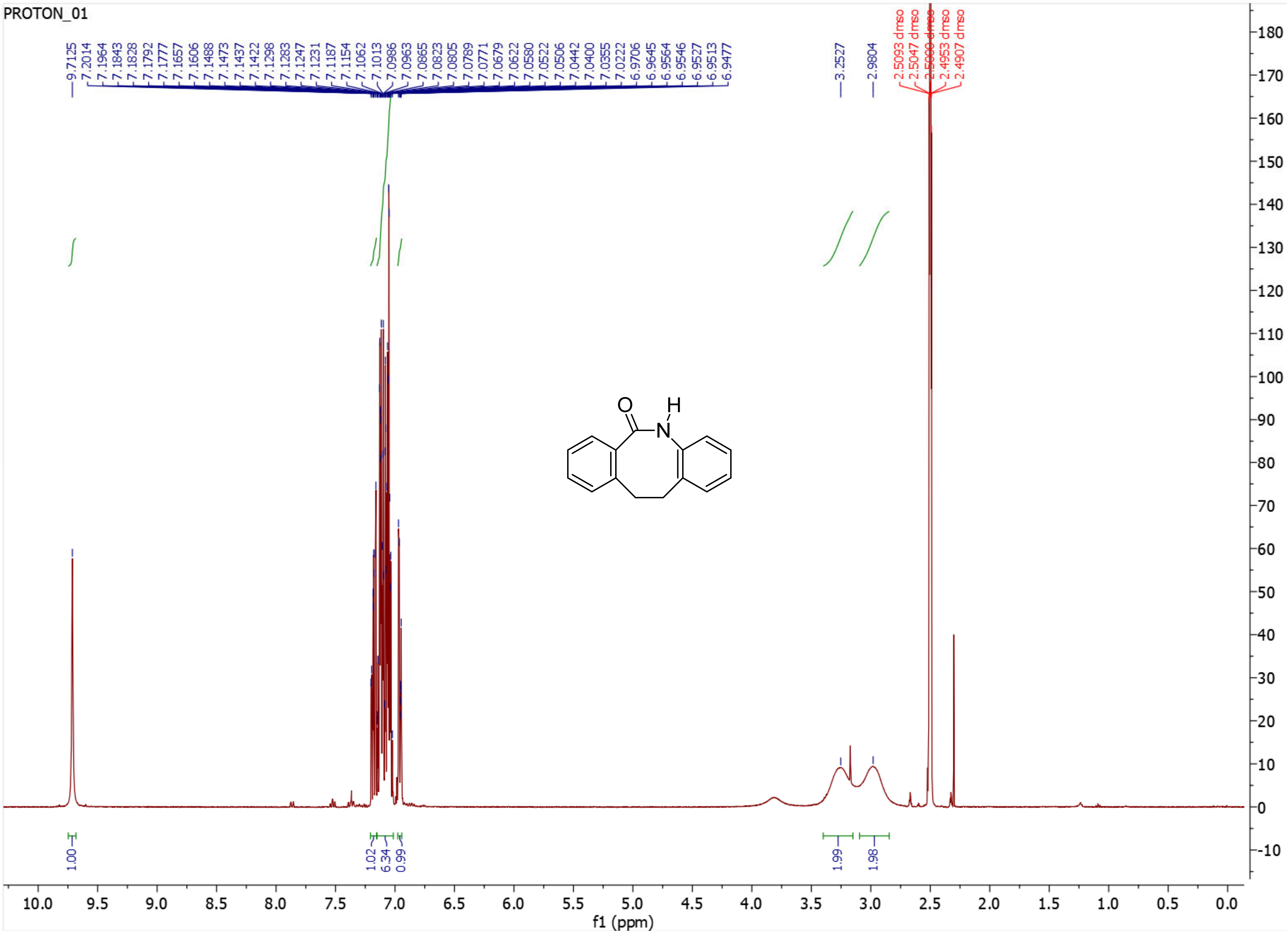

Figure S6. ^{1}H NMR spectrum (DMSO-d_6) of 11,12-dihydrodibenzo[*b*,*f*]azocin-6(5*H*)-one (**15**)

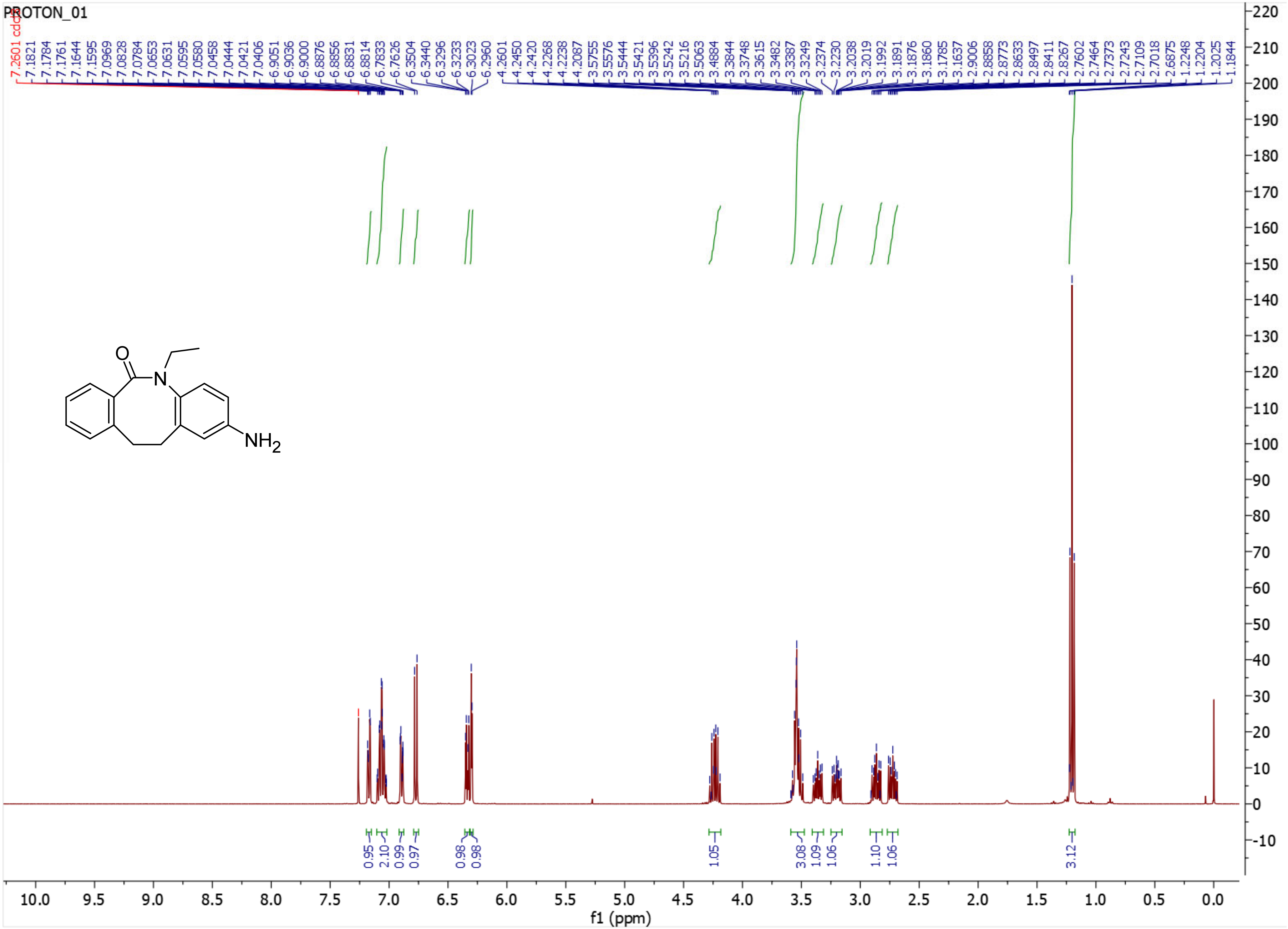

Figure S7. ^{1}H NMR spectrum ($CDCl_3$) of 2-amino-5-ethyl-11,12-dihydrodibenzo[*b*,*f*]azocin-6(5*H*)-one (**18**)

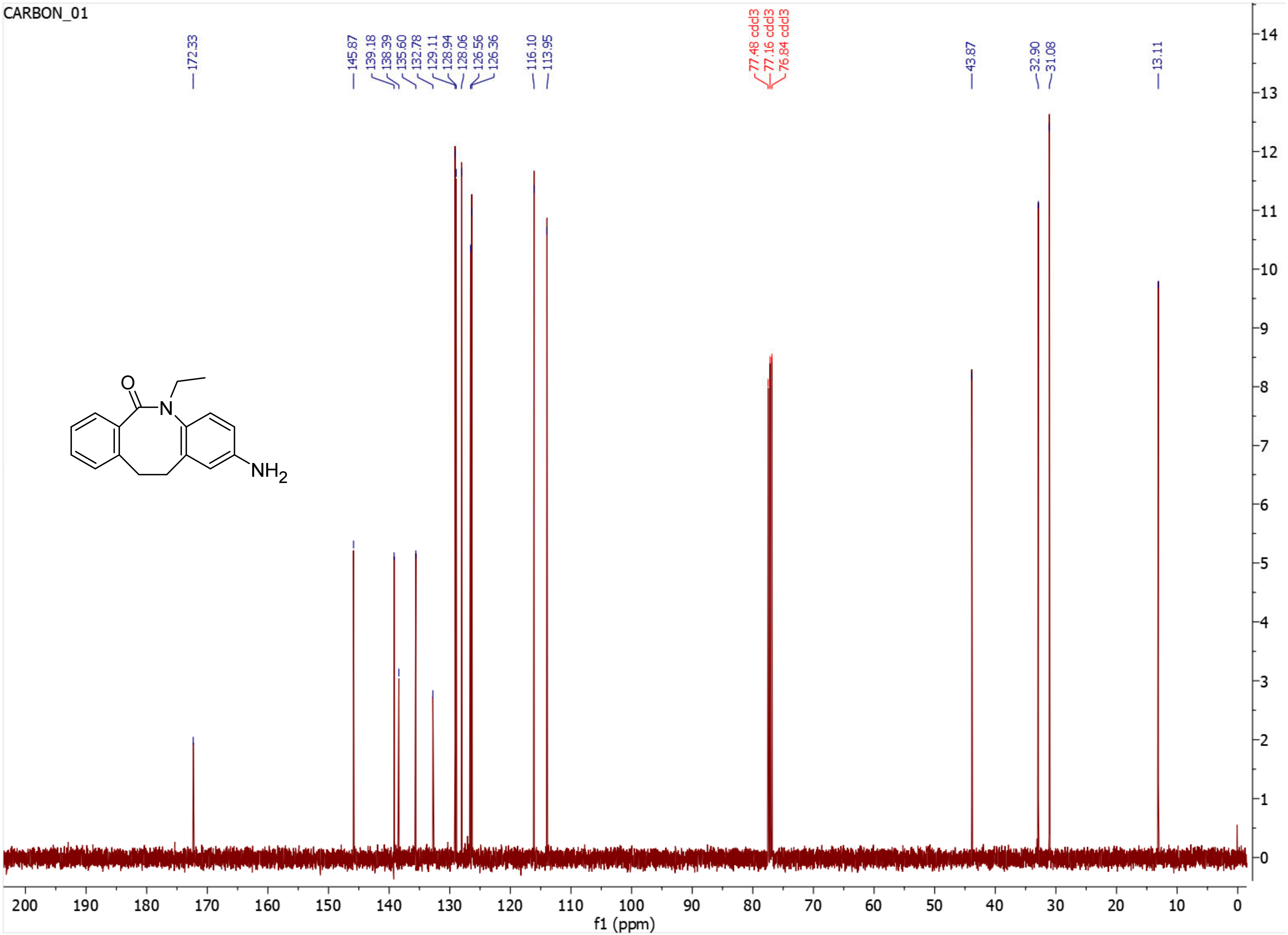

Figure S8. ^{13}C NMR spectrum ($CDCl_3$) of 2-amino-5-ethyl-11,12-dihydrodibenzo[*b*,*f*]azocin-6(5*H*)-one (**18**)

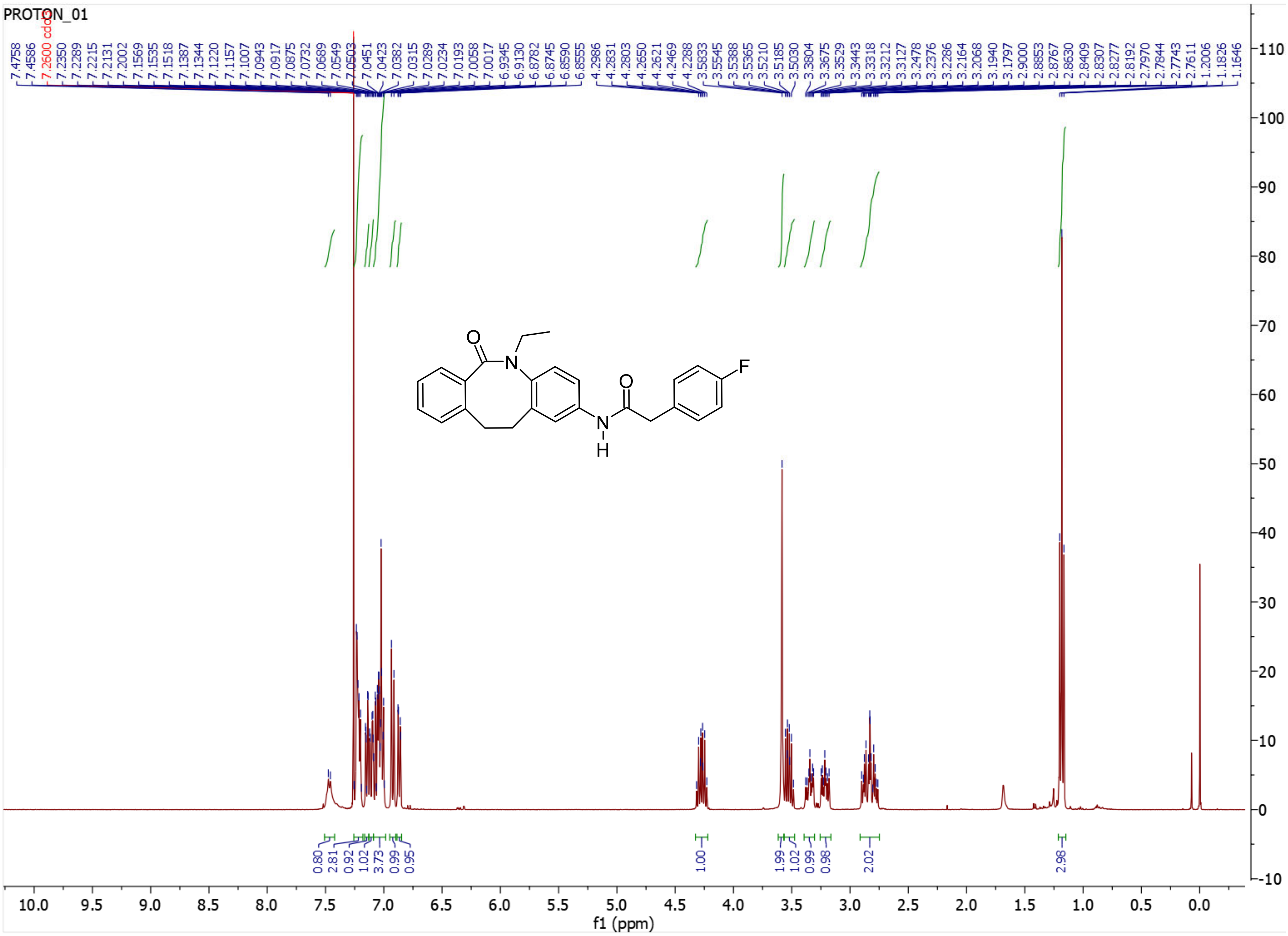

Figure S9. ^{1}H NMR spectrum ($CDCl_3$) of *N*-(5-ethyl-6-oxo-5,6,11,12-tetrahydrodibenzo[*b*,*f*]azocin-2-yl)-2-(4-fluorophenyl)acetamide (**9**)

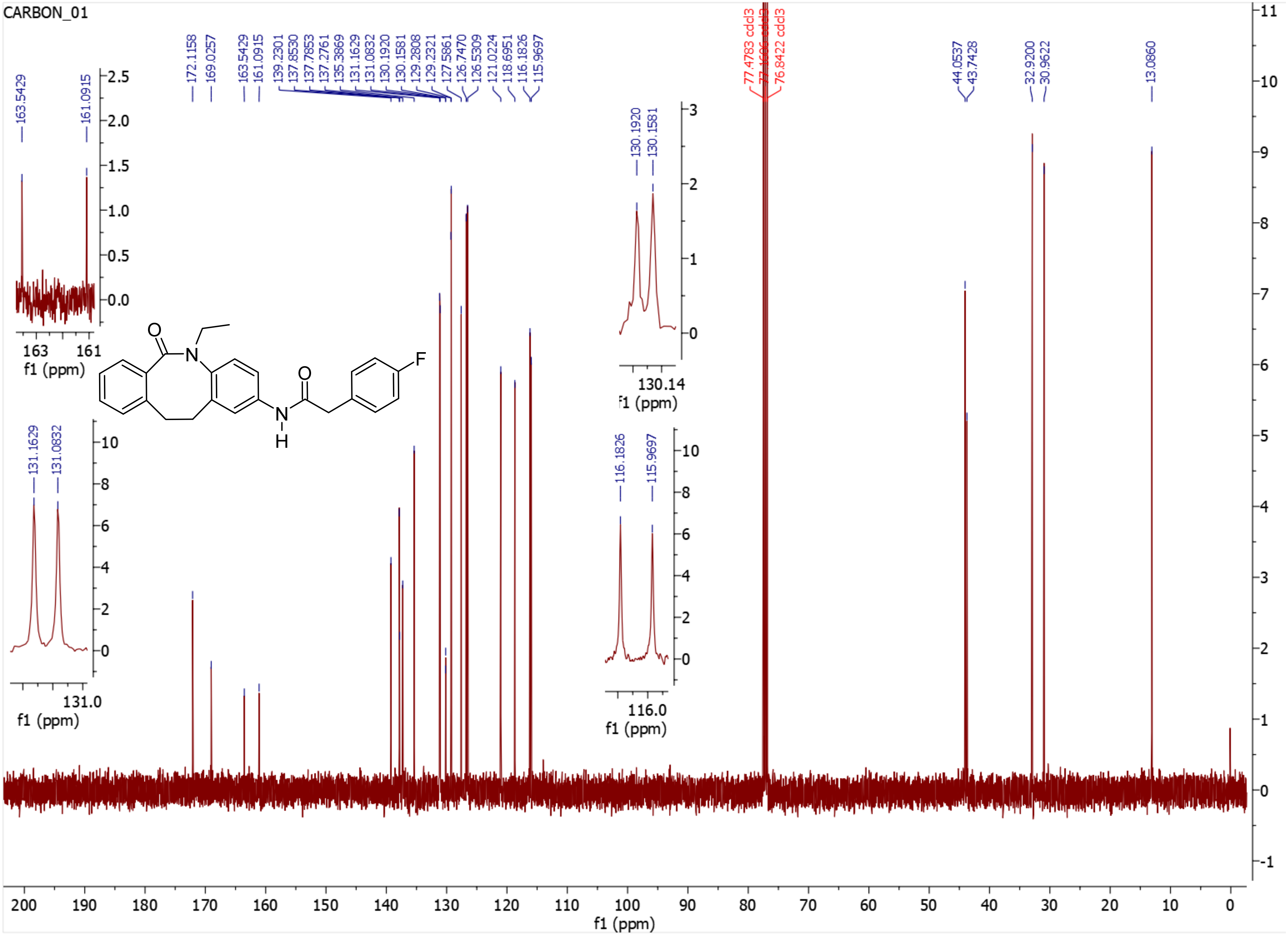

Figure S10. ^{13}C NMR spectrum ($CDCl_3$) of *N*-(5-ethyl-6-oxo-5,6,11,12-tetrahydrodibenzo[*b*,*f*]azocin-2-yl)-2-(4-fluorophenyl)acetamide (**9**)

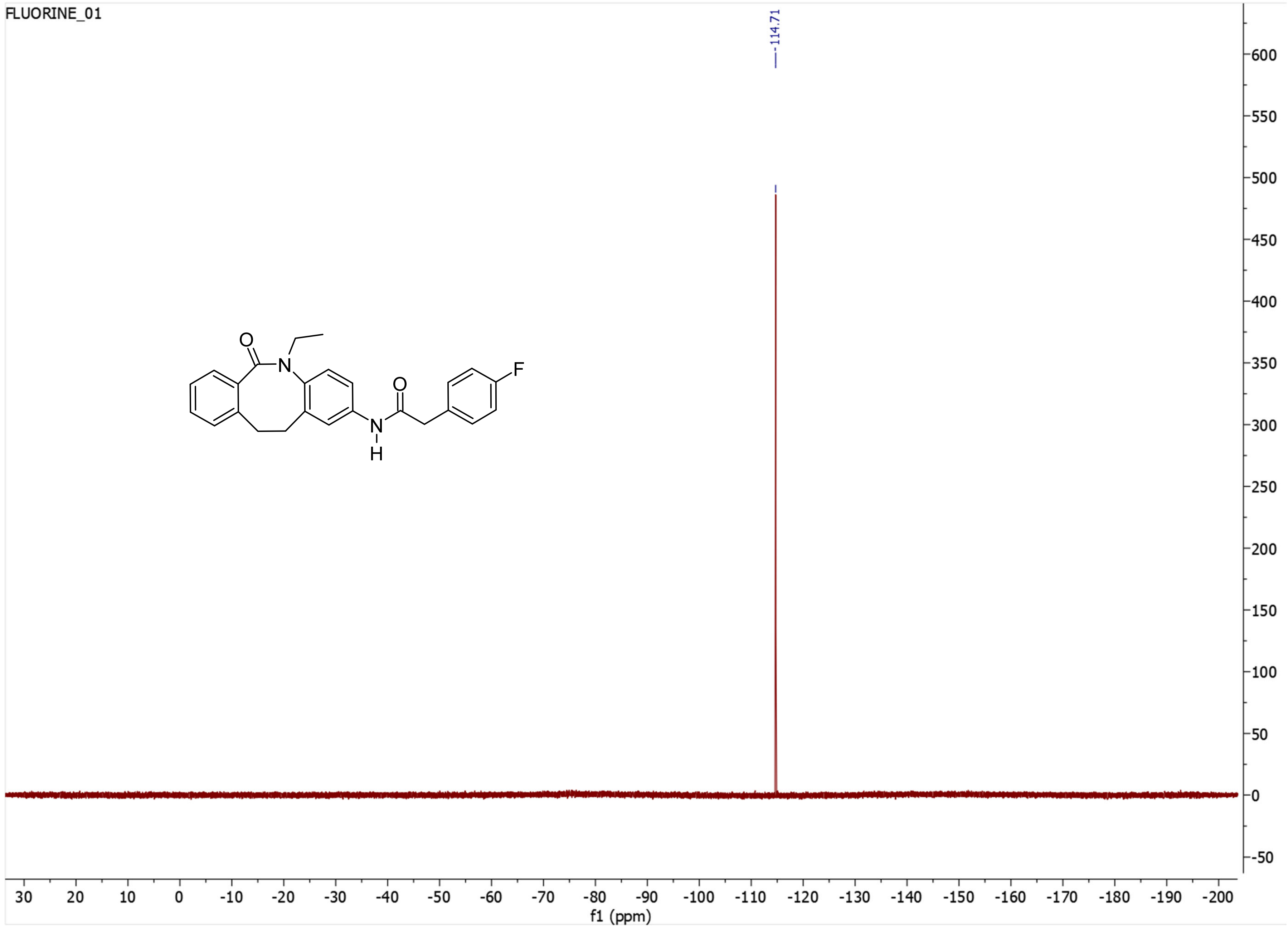

Figure S11. ^{19}F NMR spectrum ($CDCl_3$) of *N*-(5-ethyl-6-oxo-5,6,11,12-tetrahydrodibenzo[*b*,*f*]azocin-2-yl)-2-(4-fluorophenyl)acetamide (**9**)

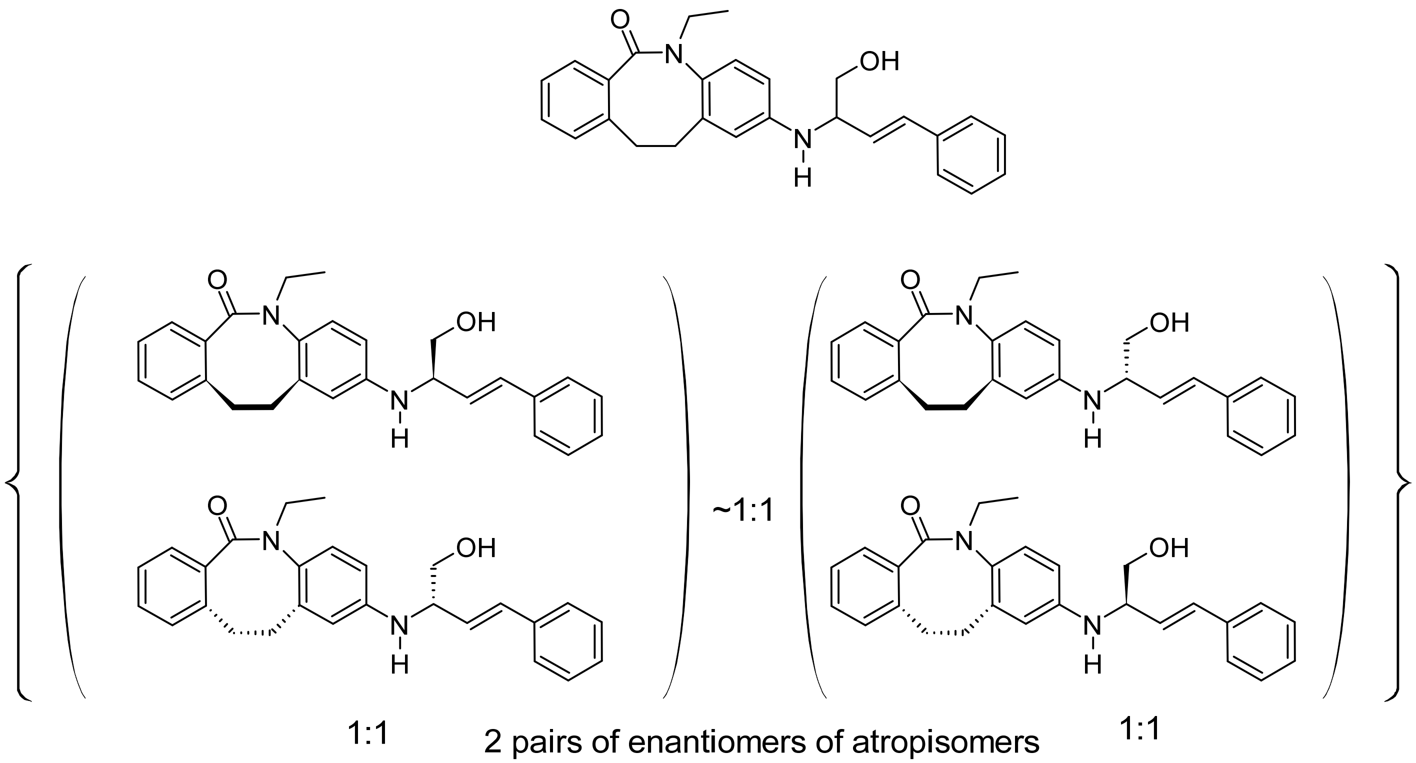

Figure S12. Structures of stereoisomers of (*E*)-5-ethyl-2-((1-hydroxy-4-phenylbut-3-en-2-yl)amino)-11,12-dihydrodibenzo[*b*,*f*]azocin-6(5*H*)-one (**11**) explaining doubled signals in ^{1}H and ^{13}C NMR

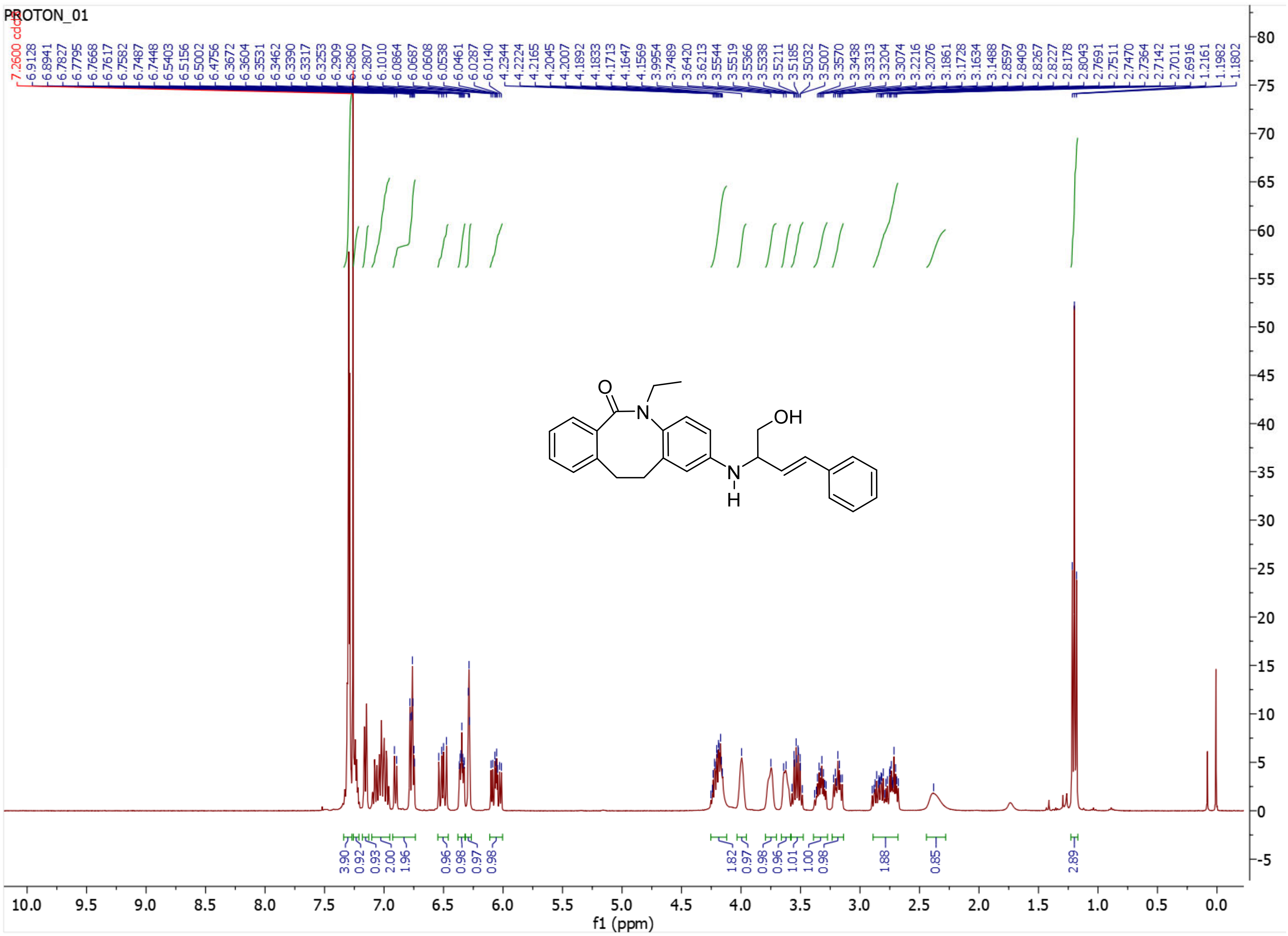

Figure S13. ^{1}H NMR spectrum ($CDCl_3$) of (*E*)-5-ethyl-2-((1-hydroxy-4-phenylbut-3-en-2-yl)amino)-11,12-dihydrodibenzo[*b*,*f*]azocin-6(5*H*)-one (**11**)

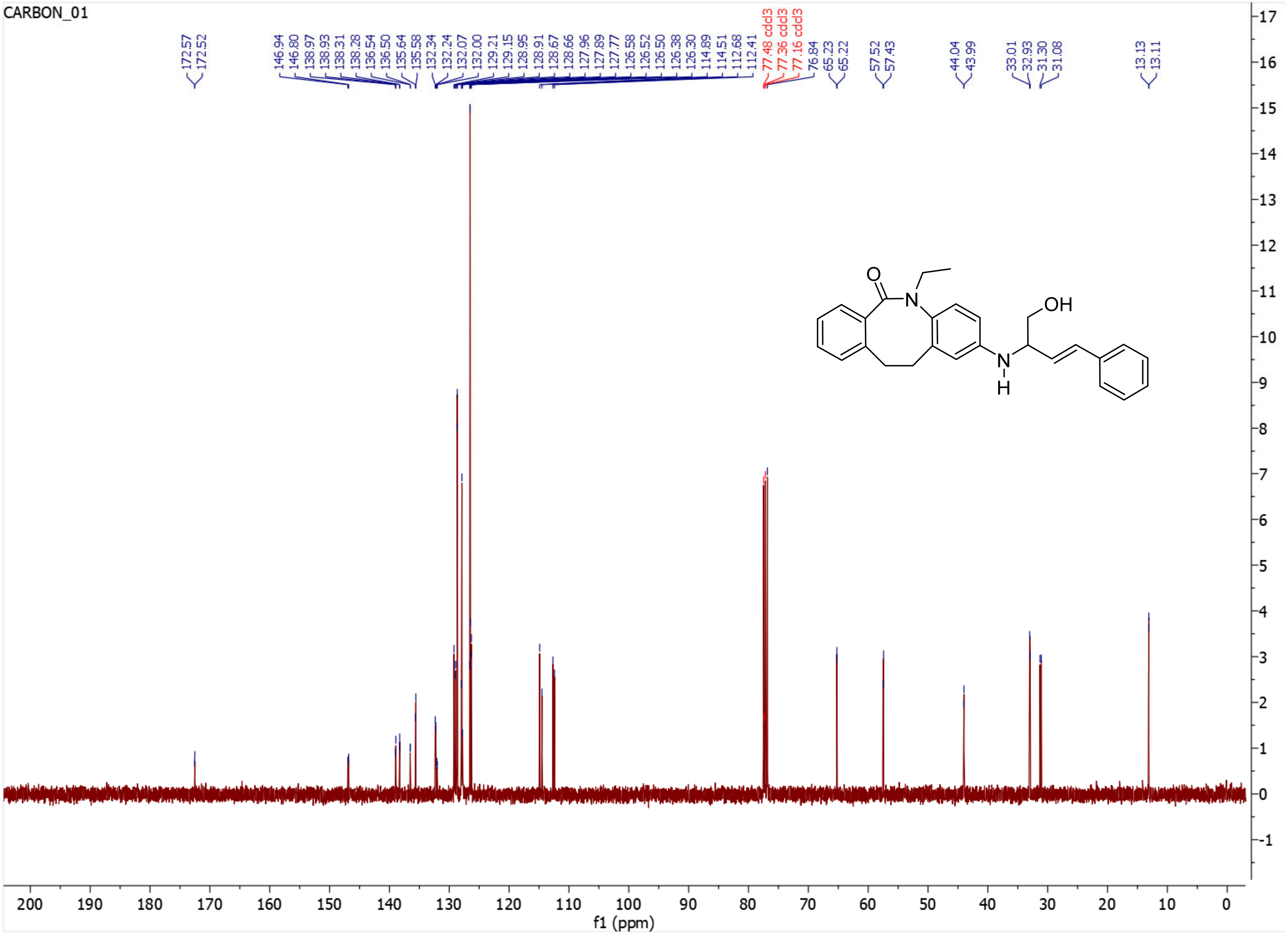

Figure S14. ^{13}C NMR spectrum ($CDCl_3$) of (*E*)-5-ethyl-2-((1-hydroxy-4-phenylbut-3-en-2-yl)amino)-11,12-dihydrodibenzo[*b*,*f*]azocin-6(5*H*)-one (**11**)

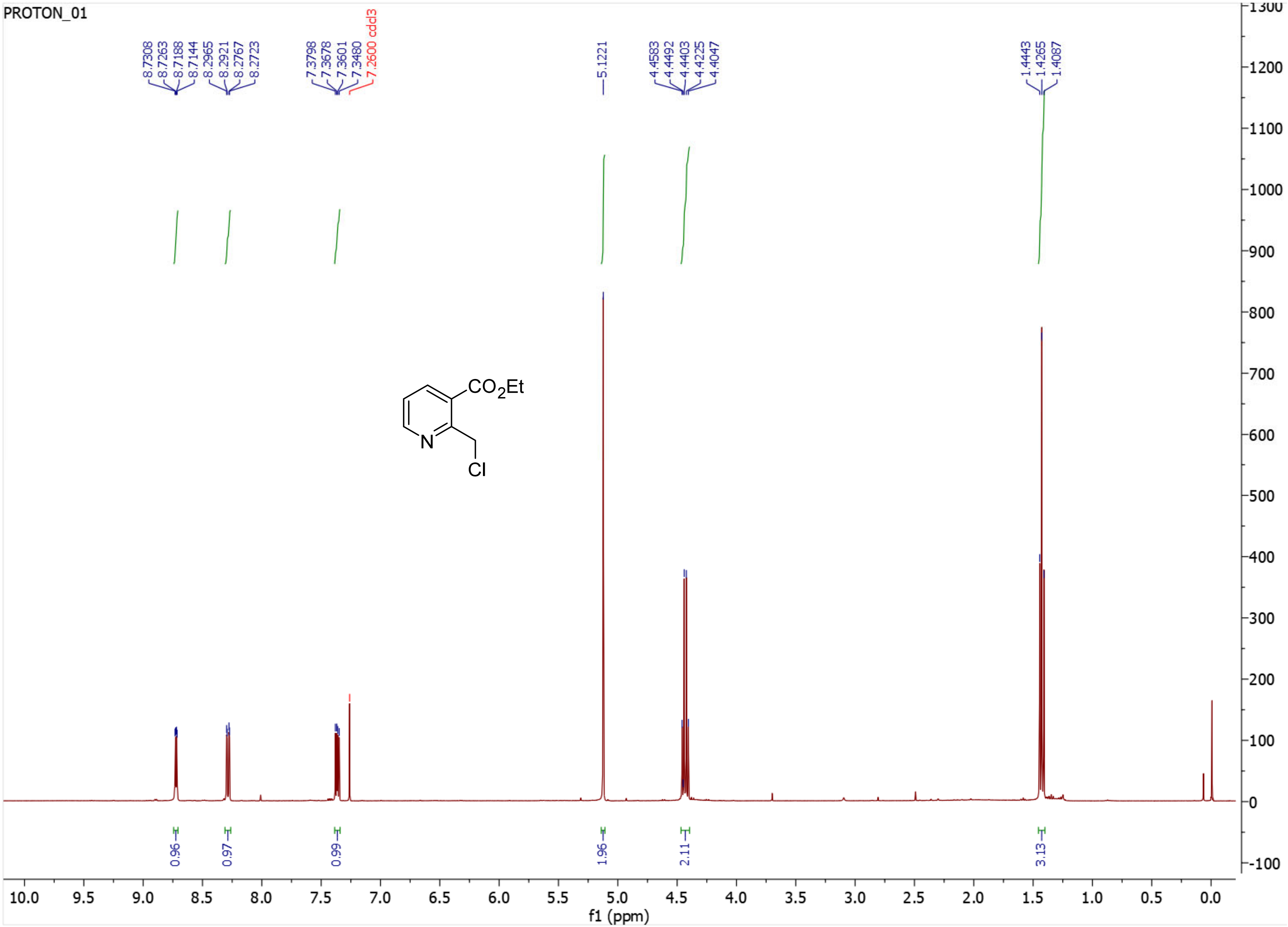

Figure S15. ^{1}H NMR spectrum ($CDCl_3$) of ethyl 2-(chloromethyl)pyridine-3-carboxylate (**20**)

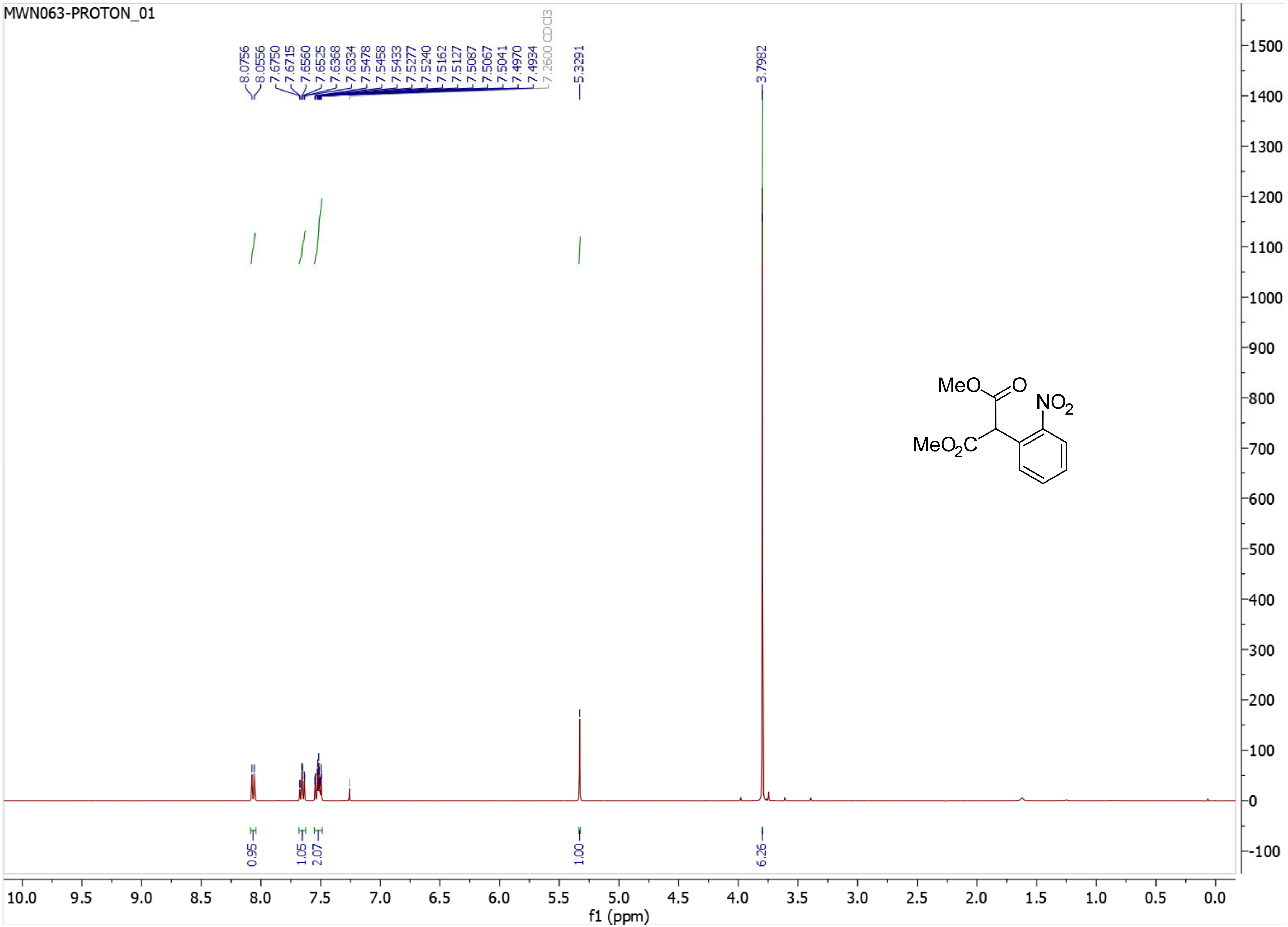

Figure S16. ^{1}H NMR spectrum ($CDCl_3$) of dimethyl (2-nitrophenyl)propanedioate (**22**)

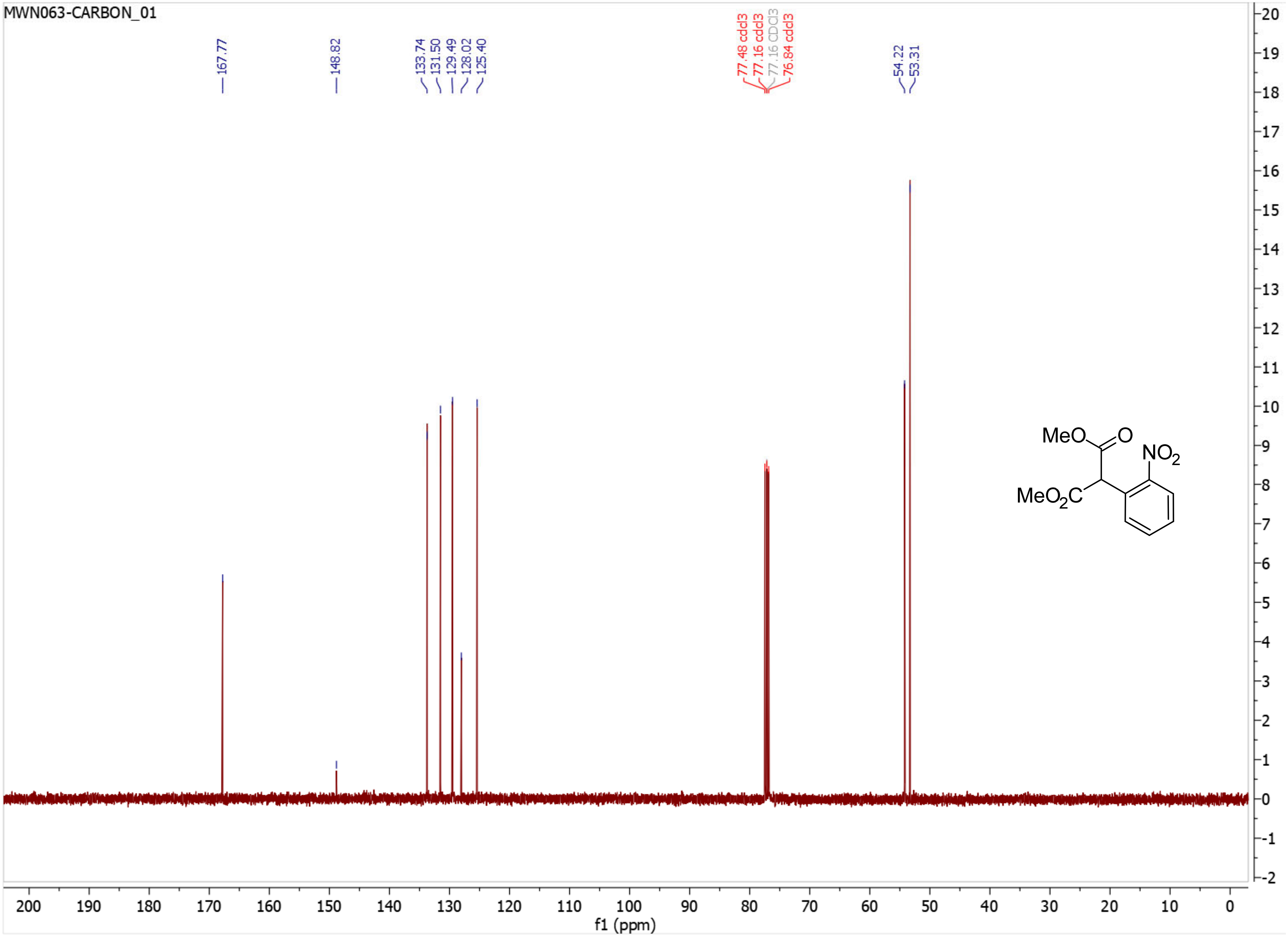

Figure S17. ^{13}C NMR spectrum ($CDCl_3$) of dimethyl (2-nitrophenyl)propanedioate (**22**)

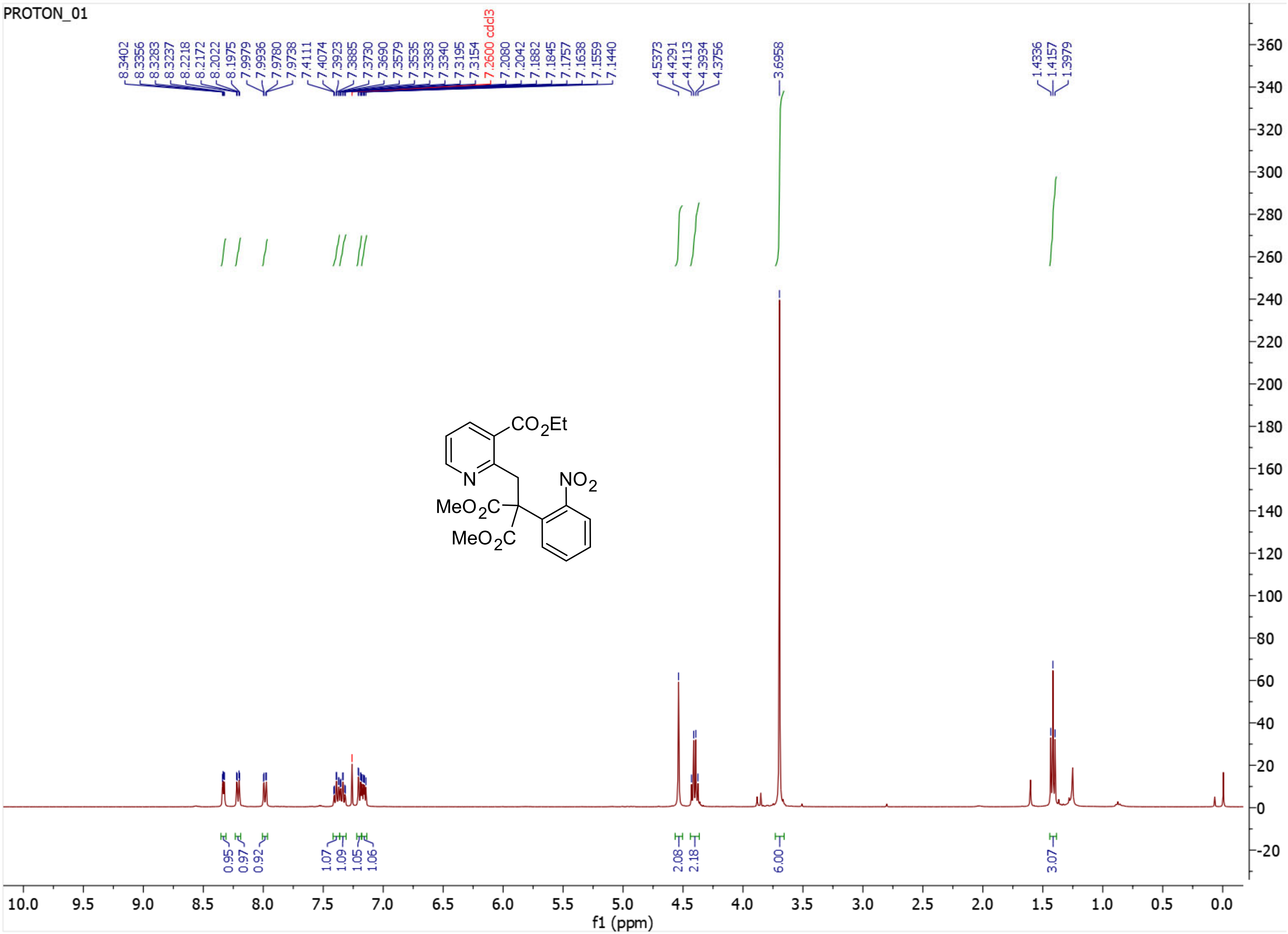

Figure S18. ^{1}H NMR spectrum ($CDCl_3$) of dimethyl {[3-(ethoxycarbonyl)pyridin-2-yl]methyl}(2-nitrophenyl)propanedioate (**23**)

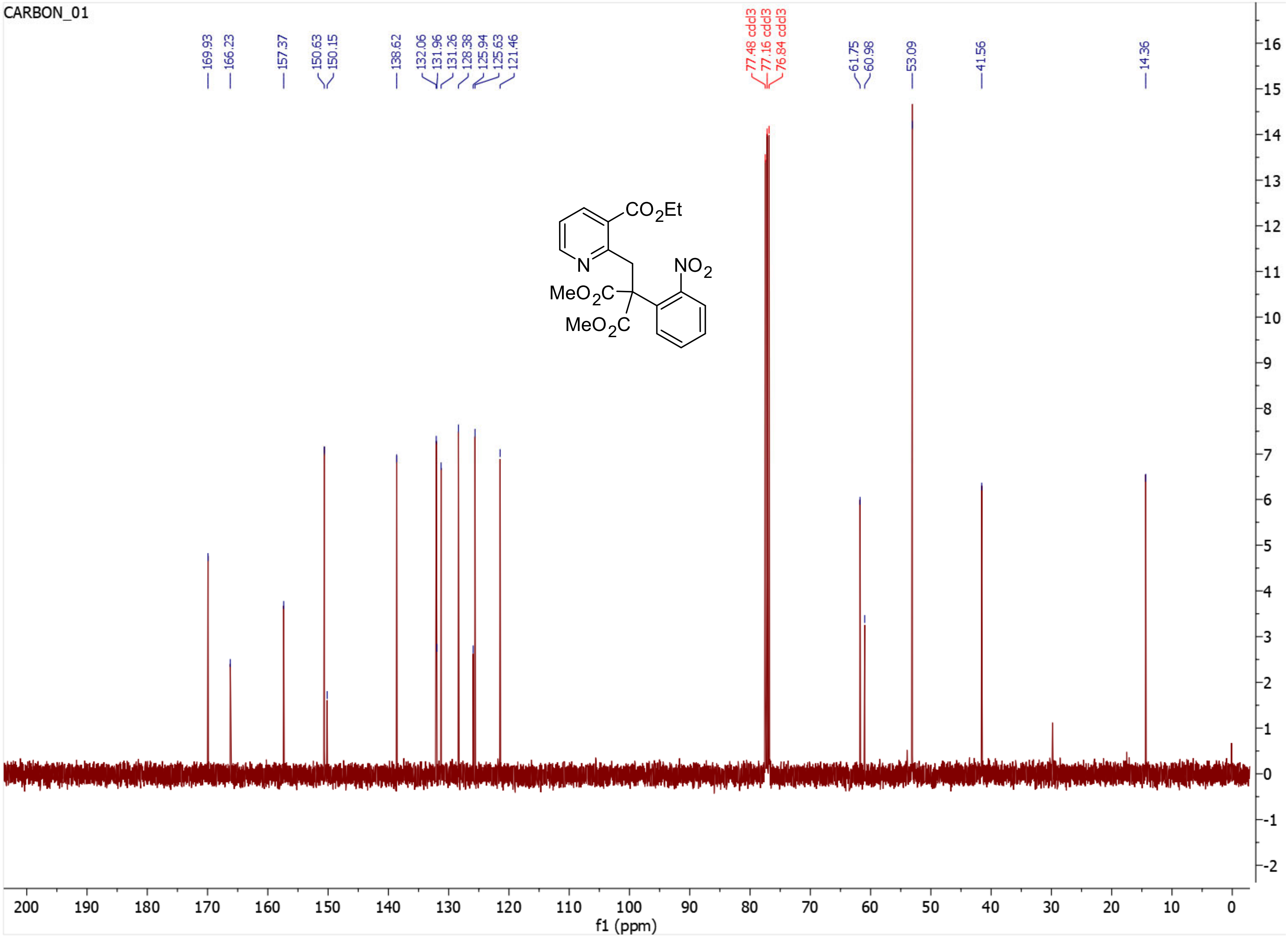

Figure S19. ^{13}C NMR spectrum ($CDCl_3$) of dimethyl {[3-(ethoxycarbonyl)pyridin-2-yl]methyl}(2-nitrophenyl)propanedioate (**23**)

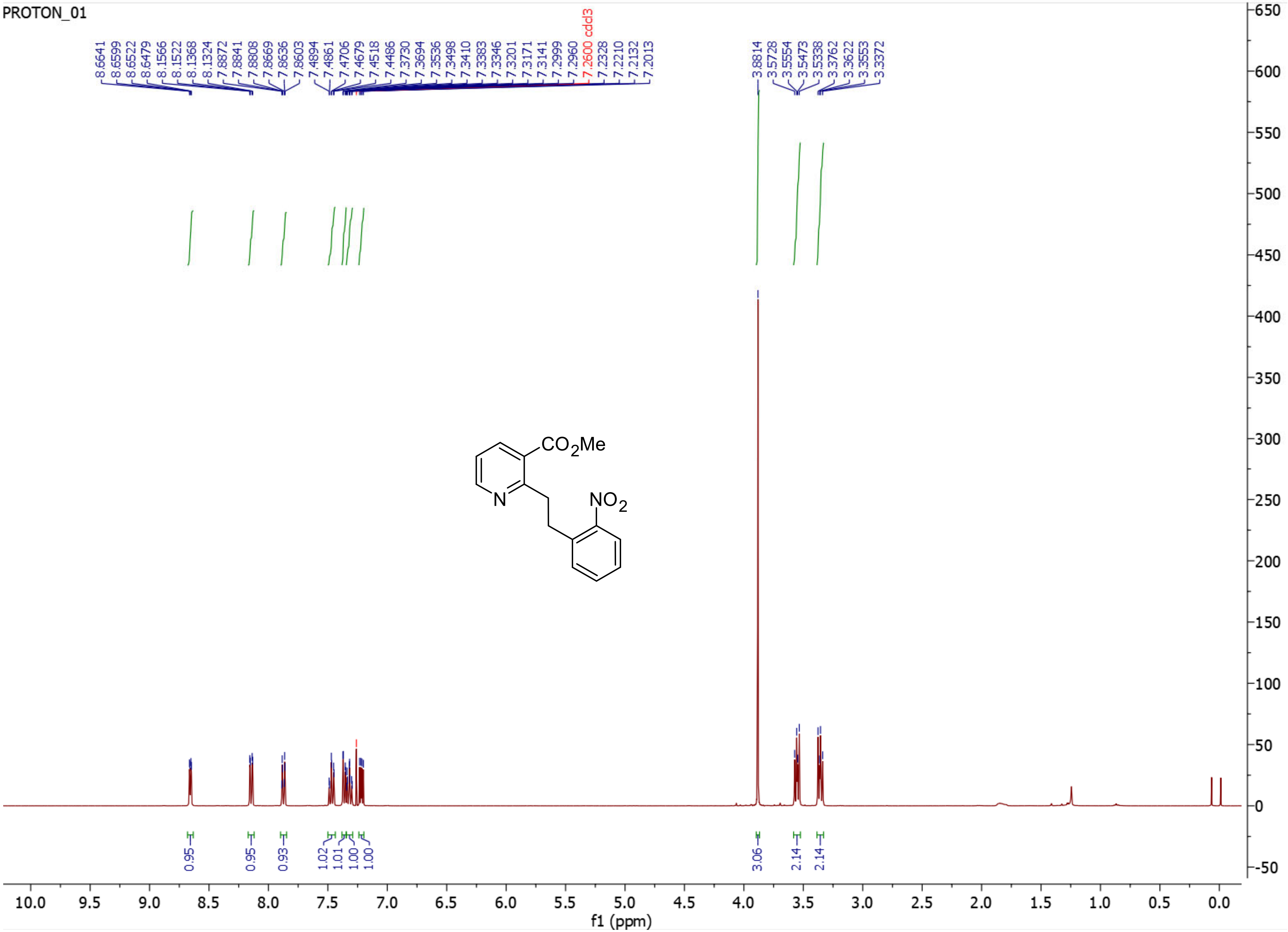

Figure S20. ^{1}H NMR spectrum ($CDCl_3$) of methyl 2-[2-(2-nitrophenyl)ethyl]pyridine-3-carboxylate (**25**)

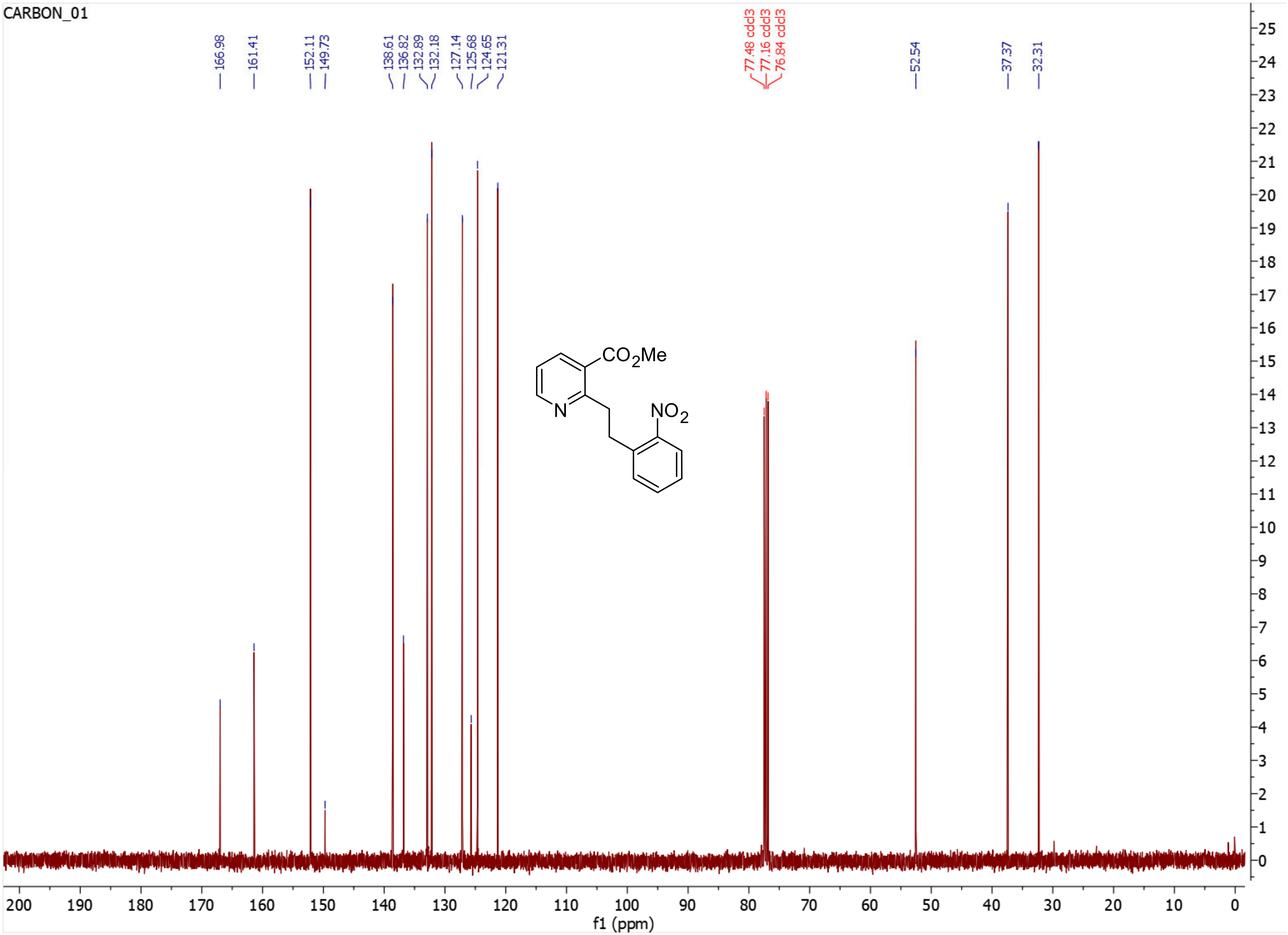

Figure S21. ^{13}C NMR spectrum ($CDCl_3$) of methyl 2-[2-(2-nitrophenyl)ethyl]pyridine-3-carboxylate (**25**)

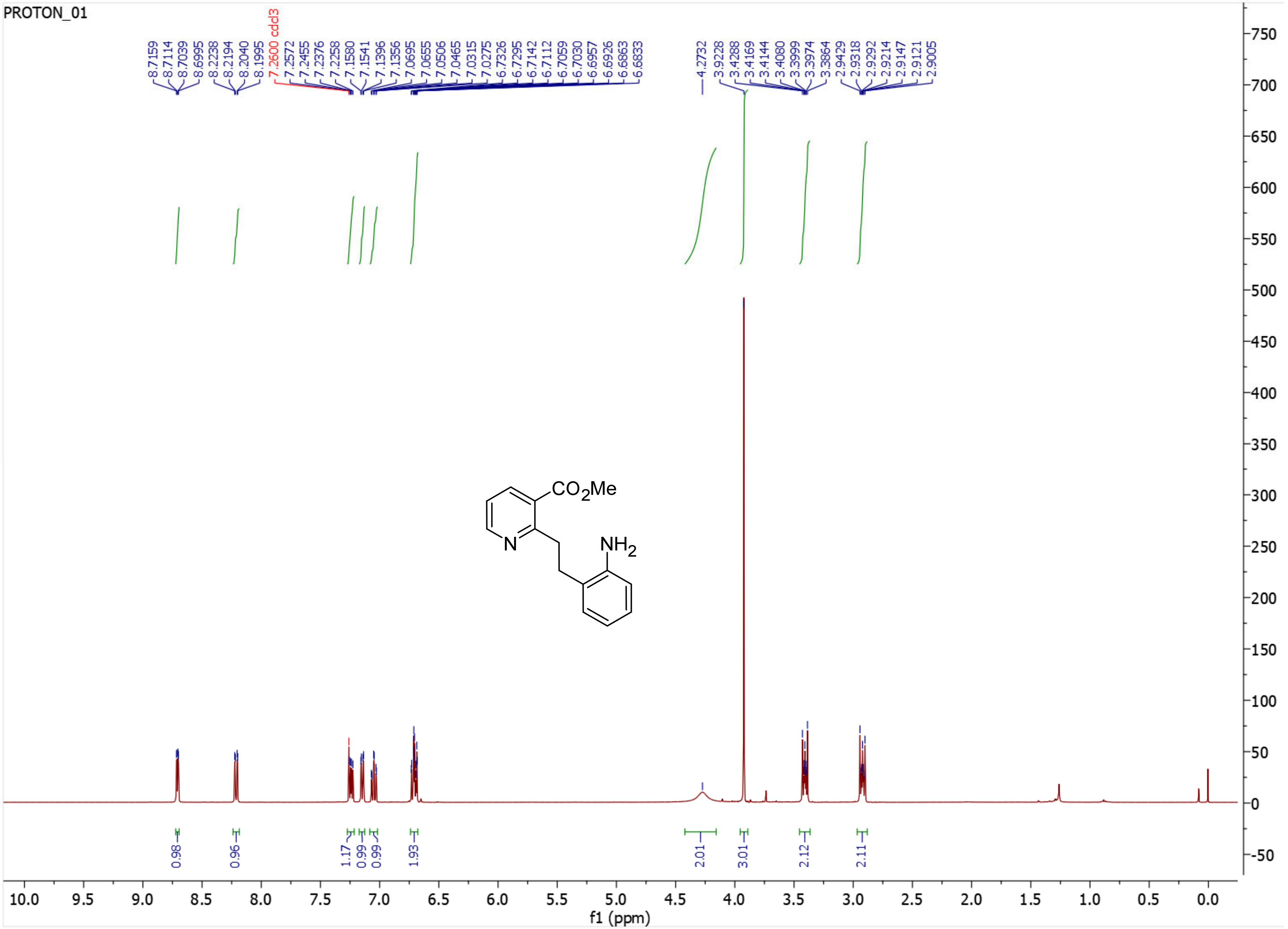

Figure S22. ^{1}H NMR spectrum ($CDCl_3$) of methyl 2-[2-(2-aminophenyl)ethyl]pyridine-3-carboxylate (**26**)

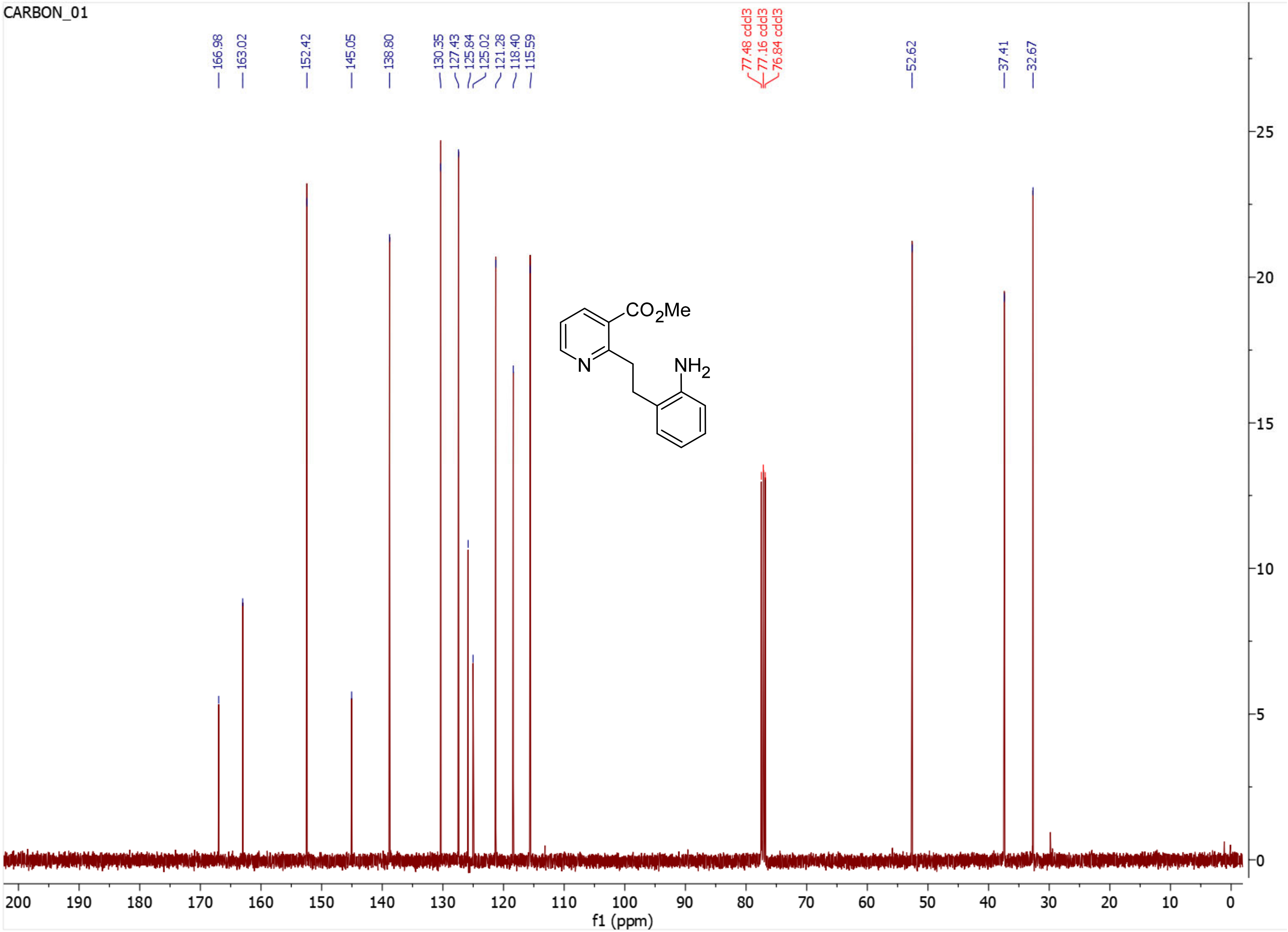

Figure S23. ^{13}C NMR spectrum ($CDCl_3$) of methyl 2-[2-(2-aminophenyl)ethyl]pyridine-3-carboxylate (**26**)

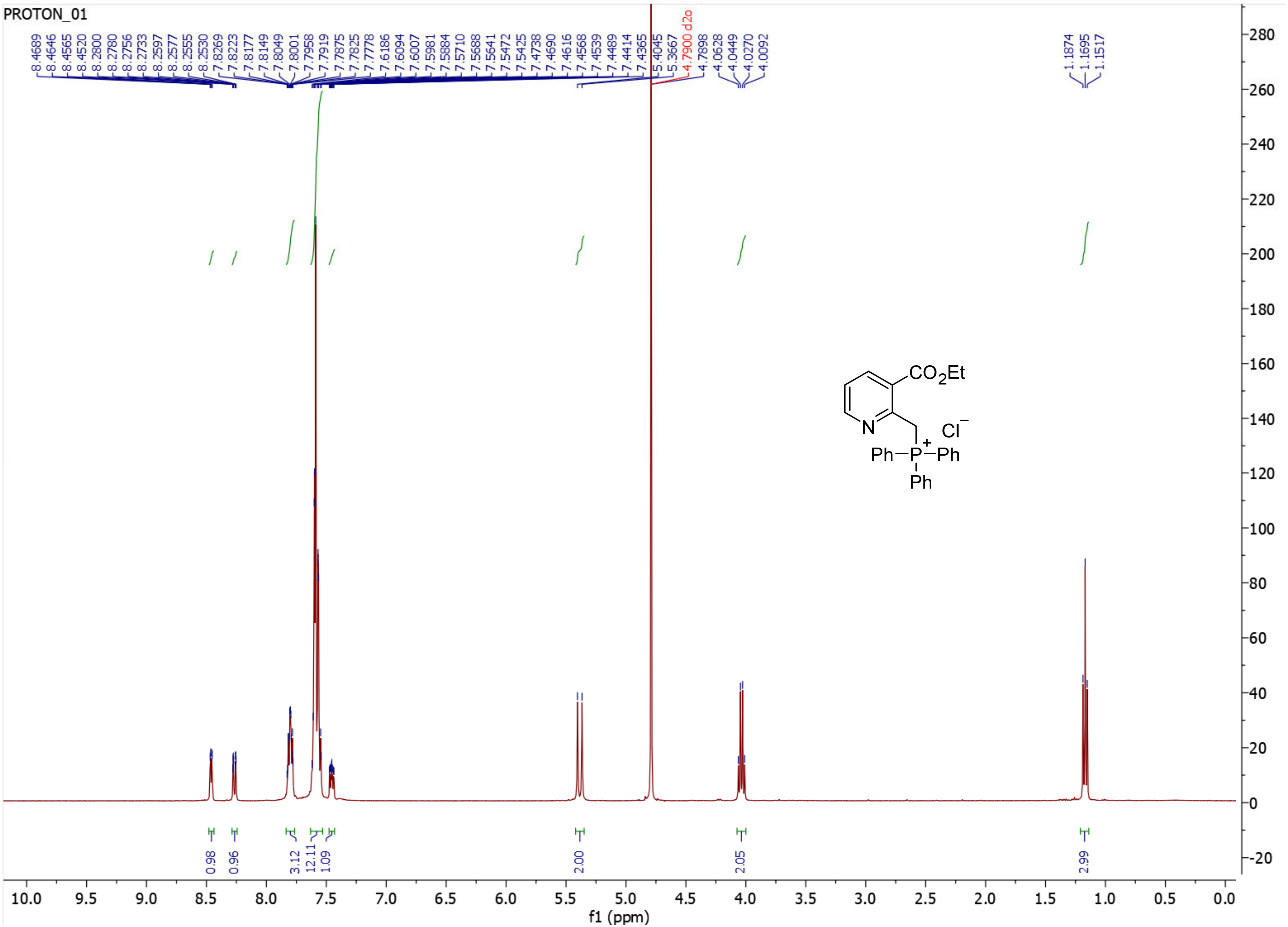

Figure S24. ^{1}H NMR spectrum (D_2O) of (3-ethoxycarbonyl-pyridin-2-ylmethyl)-triphenyl-phosphonium chloride (**28**)

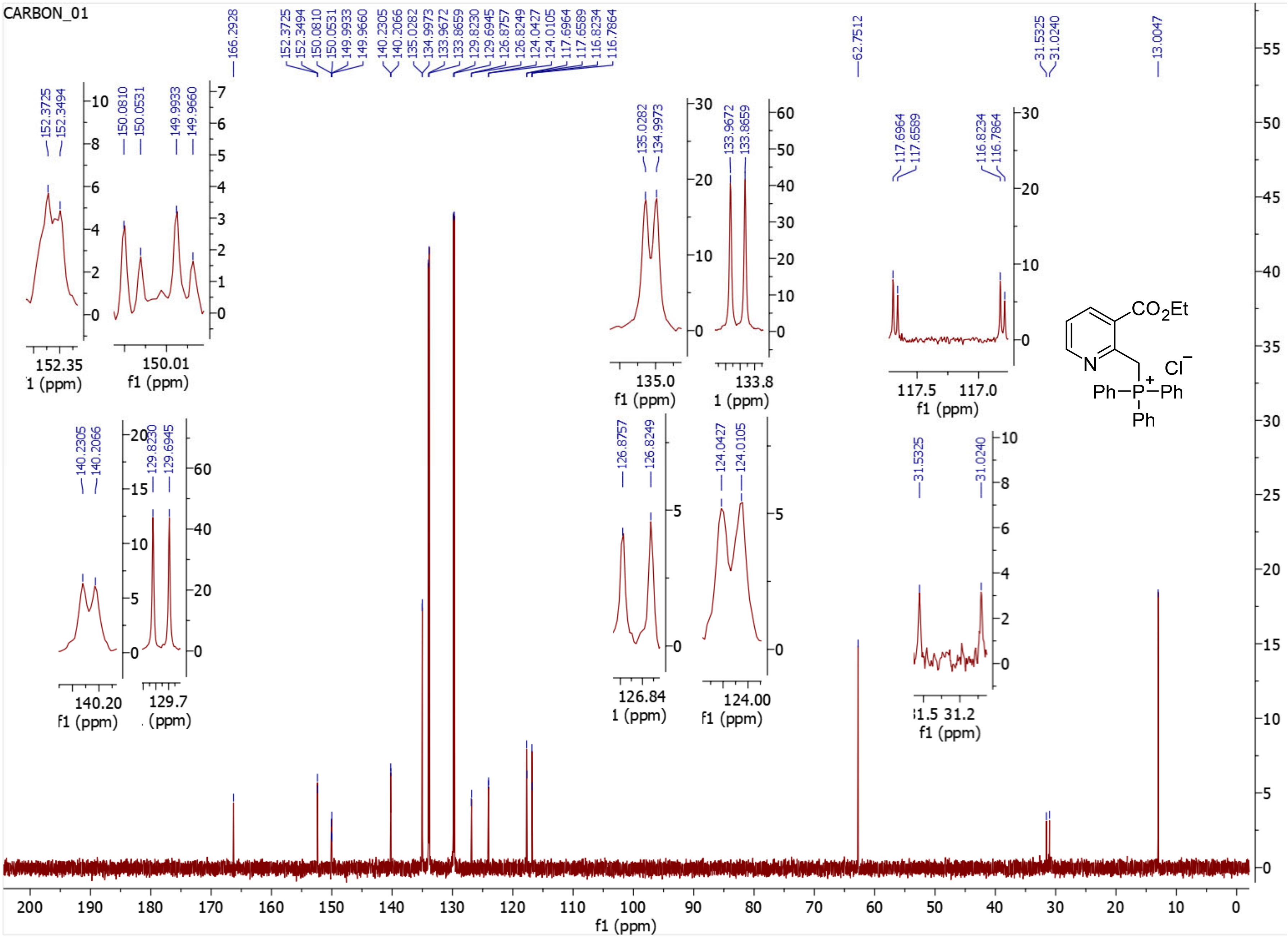

Figure S25. ^{13}C NMR spectrum (D_2O) of (3-ethoxycarbonyl-pyridin-2-ylmethyl)-triphenyl-phosphonium chloride (**28**)

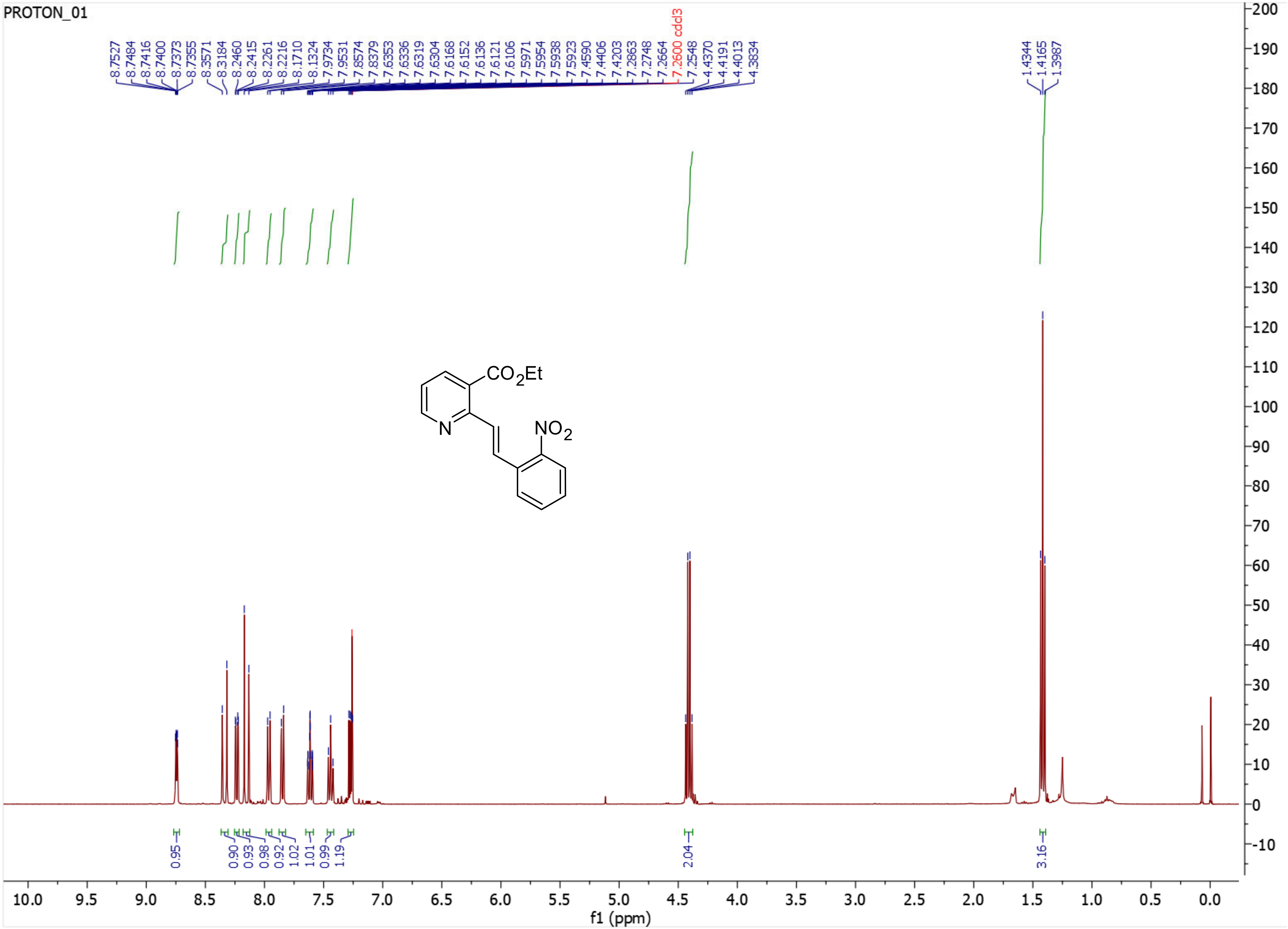

Figure S26. ^{1}H NMR spectrum ($CDCl_3$) of ethyl 2-[2-(2-nitrophenyl)ethenyl]pyridine-3-carboxylate (**29**)

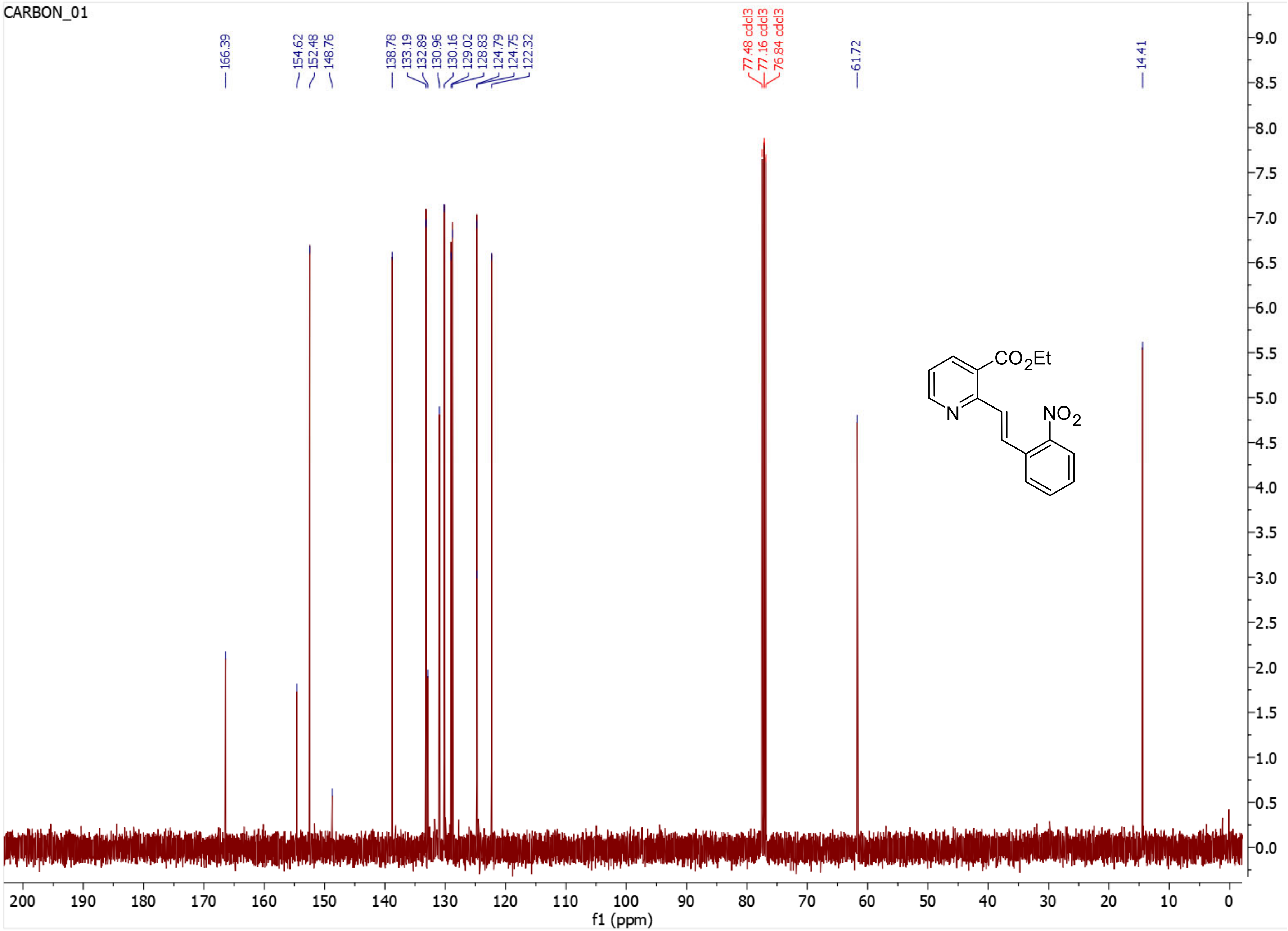

Figure S27. ^{13}C NMR spectrum ($CDCl_3$) of ethyl 2-[2-(2-nitrophenyl)ethenyl]pyridine-3-carboxylate (**29**)

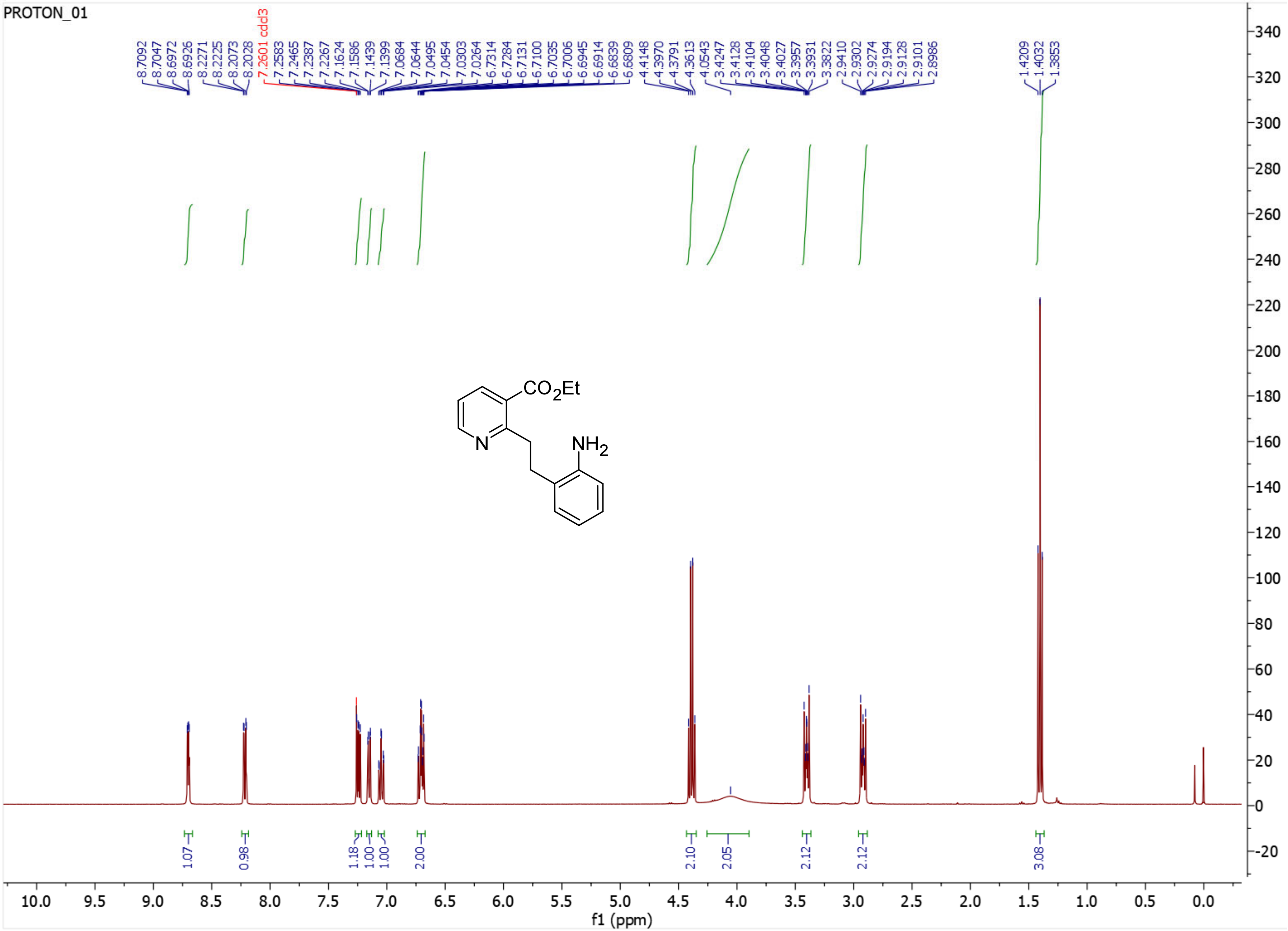

Figure S28. ^{1}H NMR spectrum ($CDCl_3$) of ethyl 2-[2-(2-aminophenyl)ethyl]pyridine-3-carboxylate (**27**)

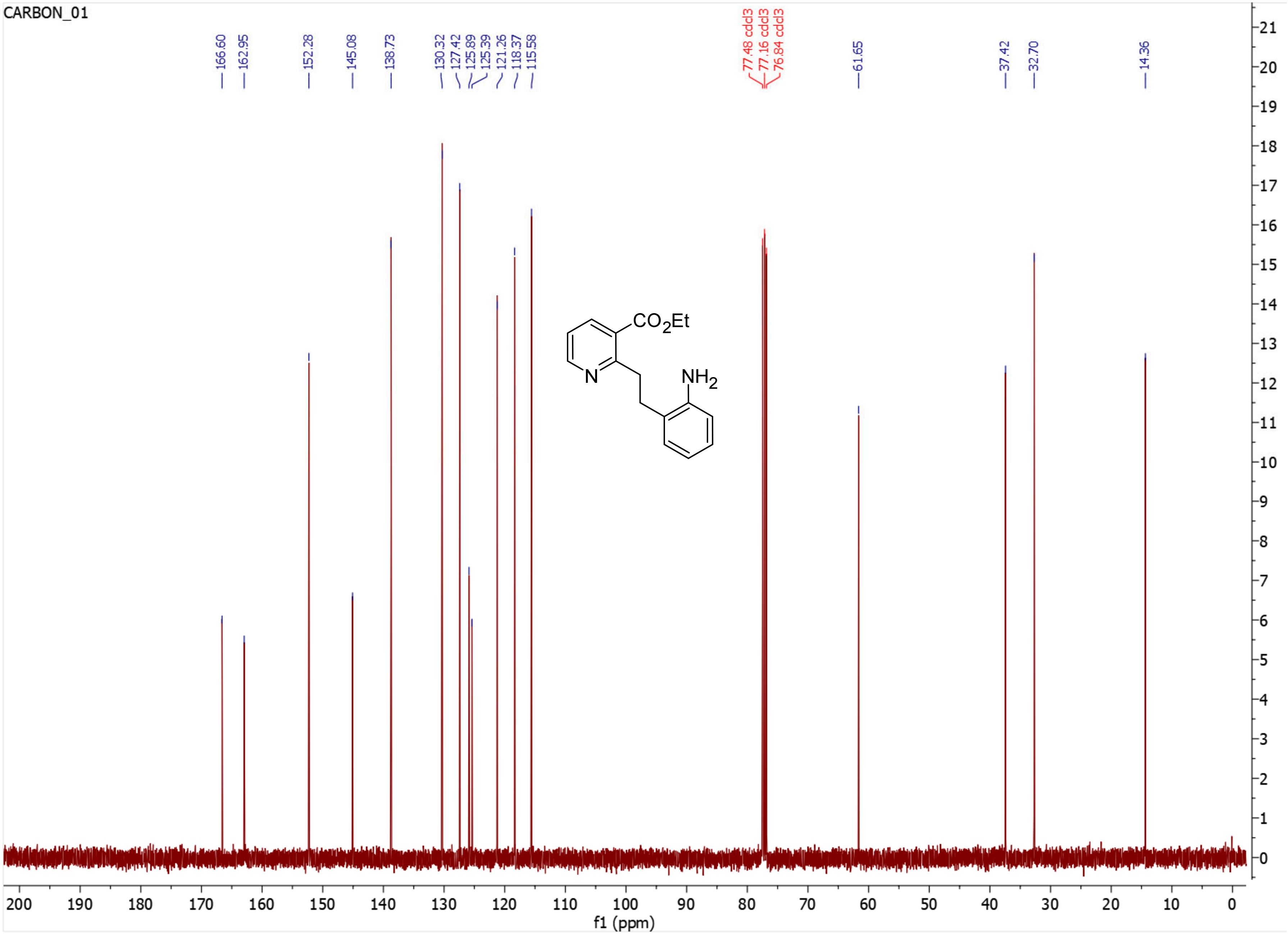

Figure S29. ^{13}C NMR spectrum ($CDCl_3$) of ethyl 2-[2-(2-aminophenyl)ethyl]pyridine-3-carboxylate (**27**)

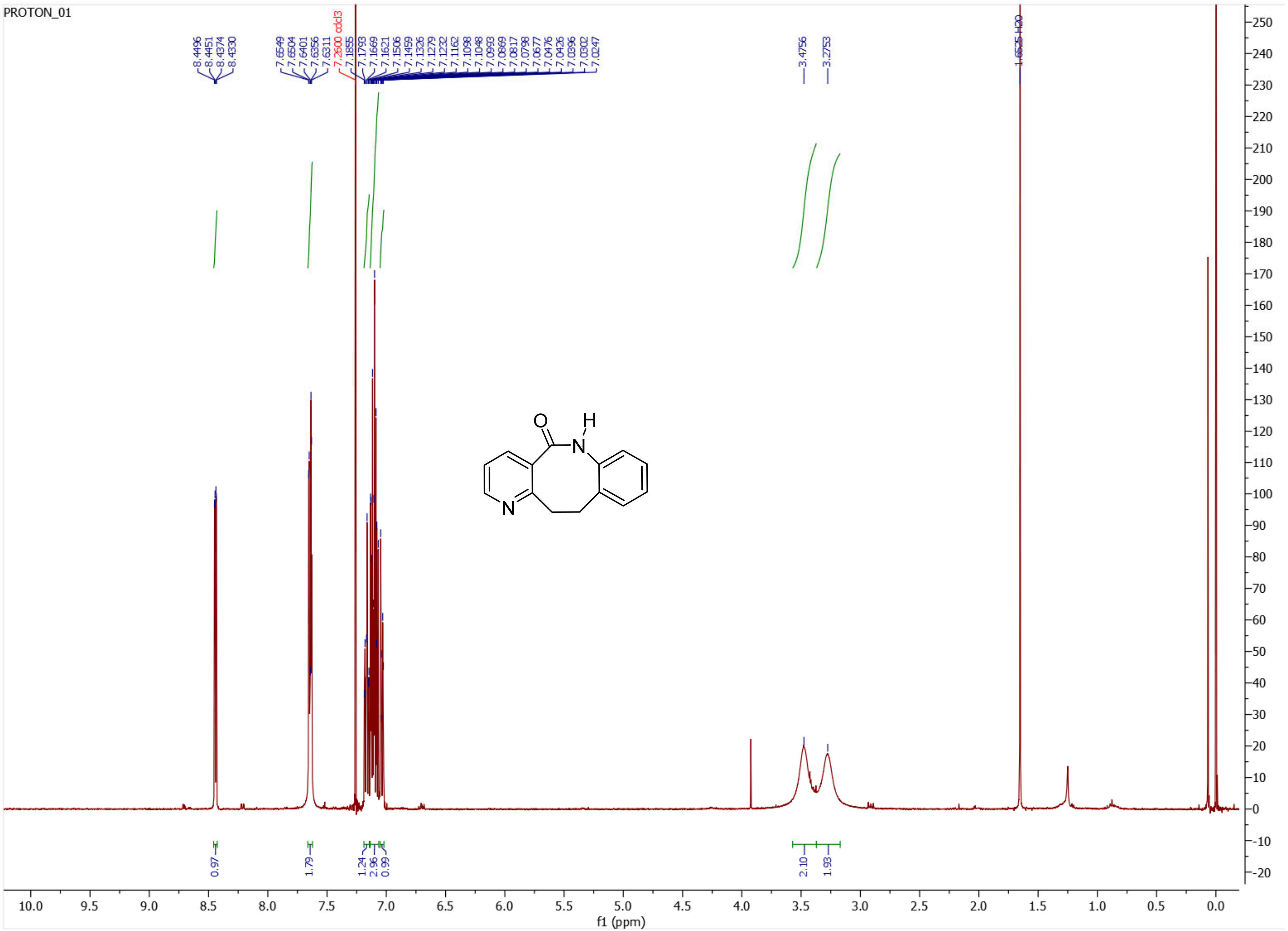

Figure S30. ^{1}H NMR spectrum ($CDCl_3$) of 11,12-dihydropyrido[3,2-*c*][1]benzazocin-5(6*H*)-one (**30**)

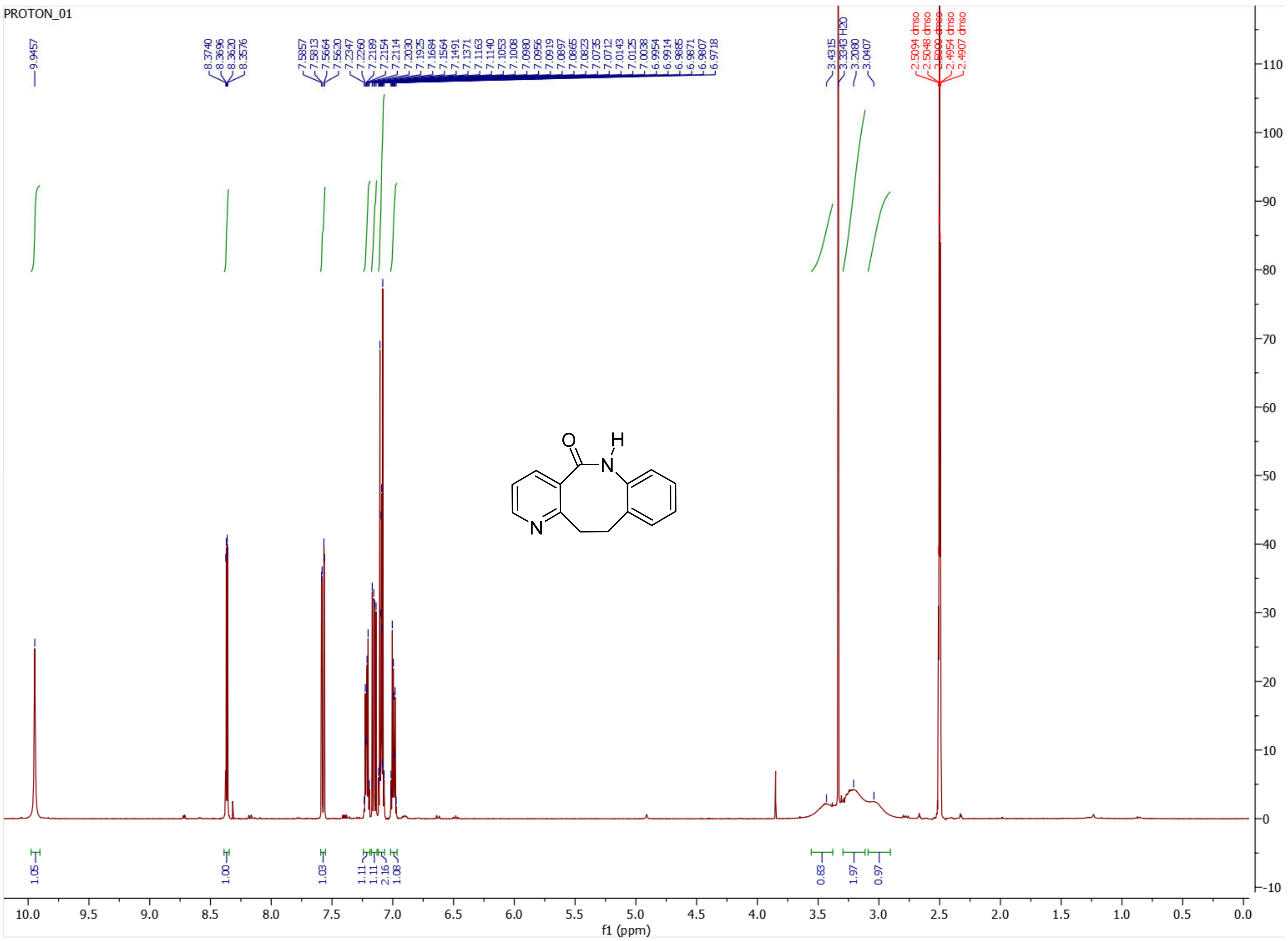

Figure S31. ^{1}H NMR spectrum (DMSO-d_6) of 11,12-dihydropyrido[3,2-*c*][1]benzazocin-5(6*H*)-one (**30**)

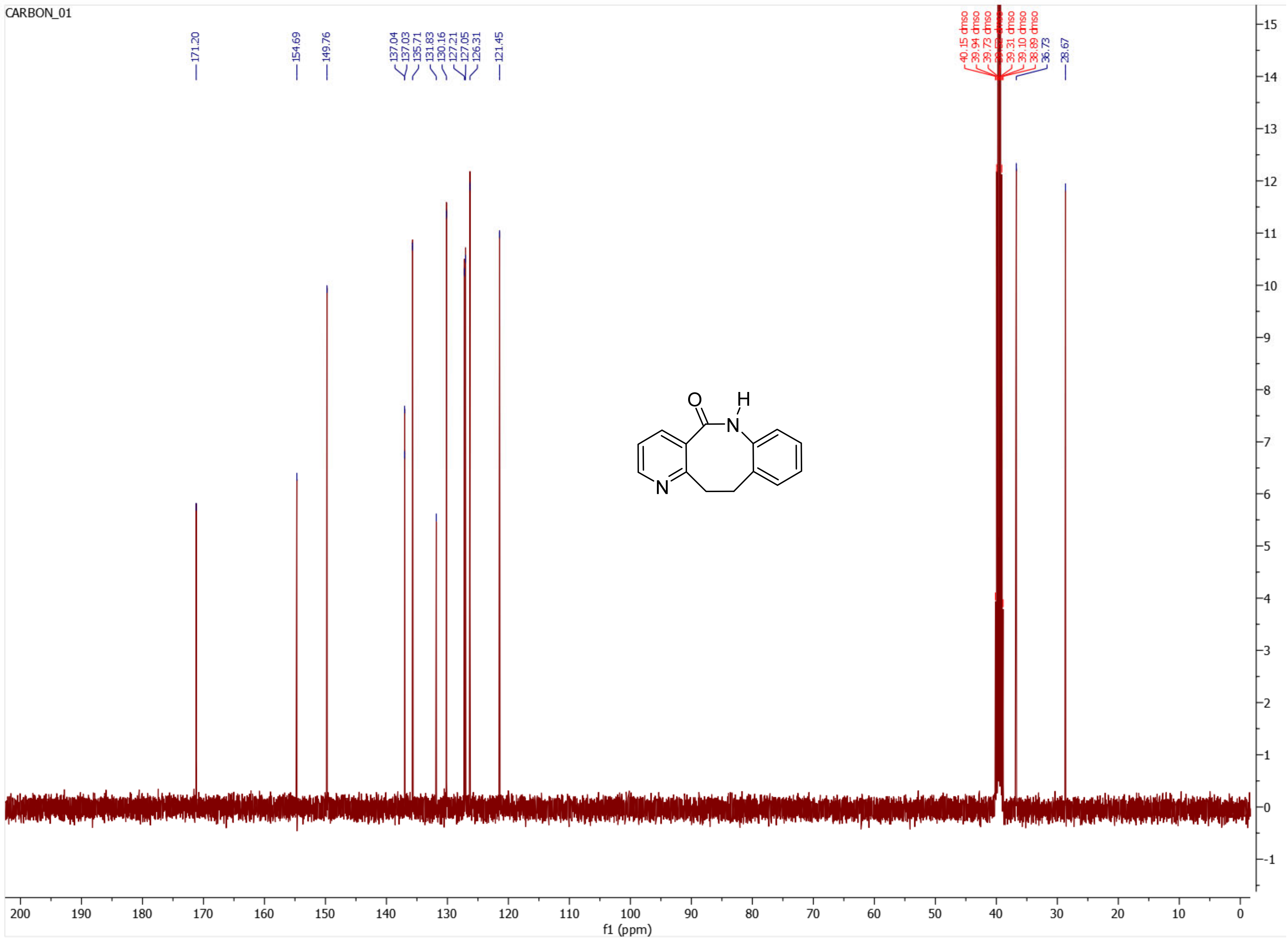

Figure S32. ^{13}C NMR spectrum (DMSO-d_6) of 11,12-dihydropyrido[3,2-*c*][1]benzazocin-5(6*H*)-one (**30**)

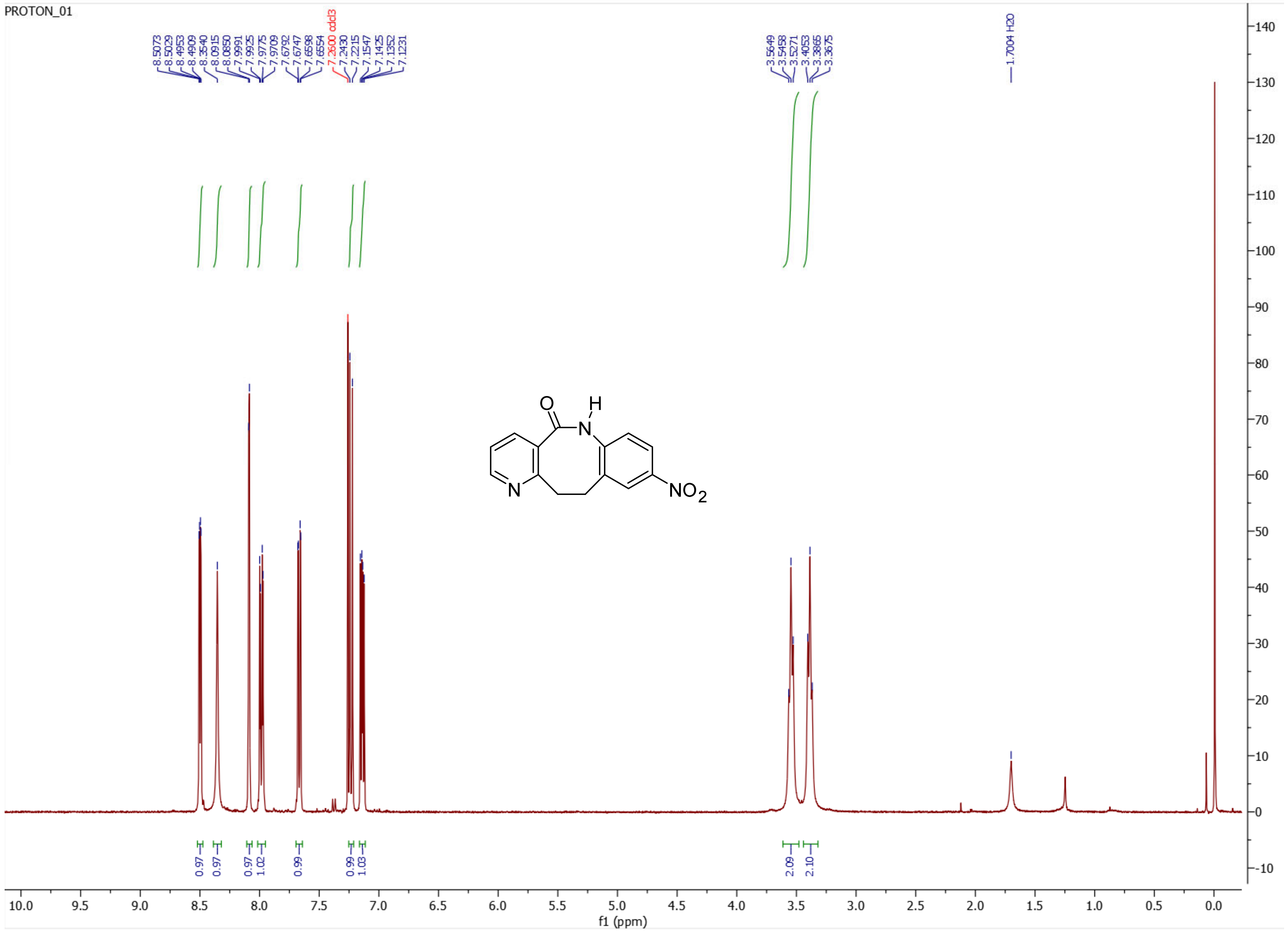

Figure S33. ^{1}H NMR spectrum ($CDCl_3$) of 9-nitro-11,12-dihydropyrido[3,2-*c*][1]benzazocin-5(6*H*)-one (**31**)

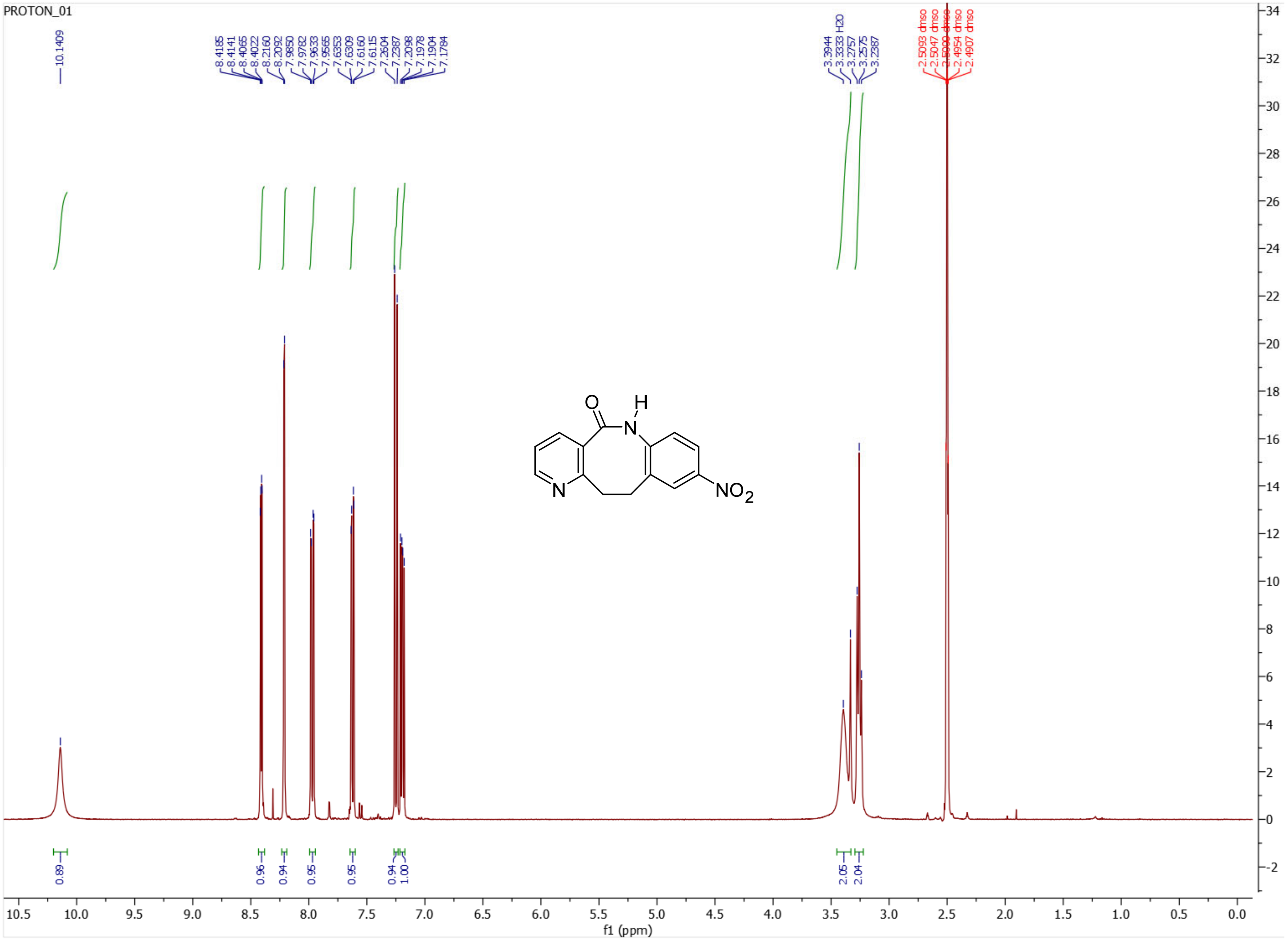

Figure S34. ^{1}H NMR spectrum (DMSO-d_6) of 9-nitro-11,12-dihydropyrido[3,2-*c*][1]benzazocin-5(6*H*)-one (**31**)

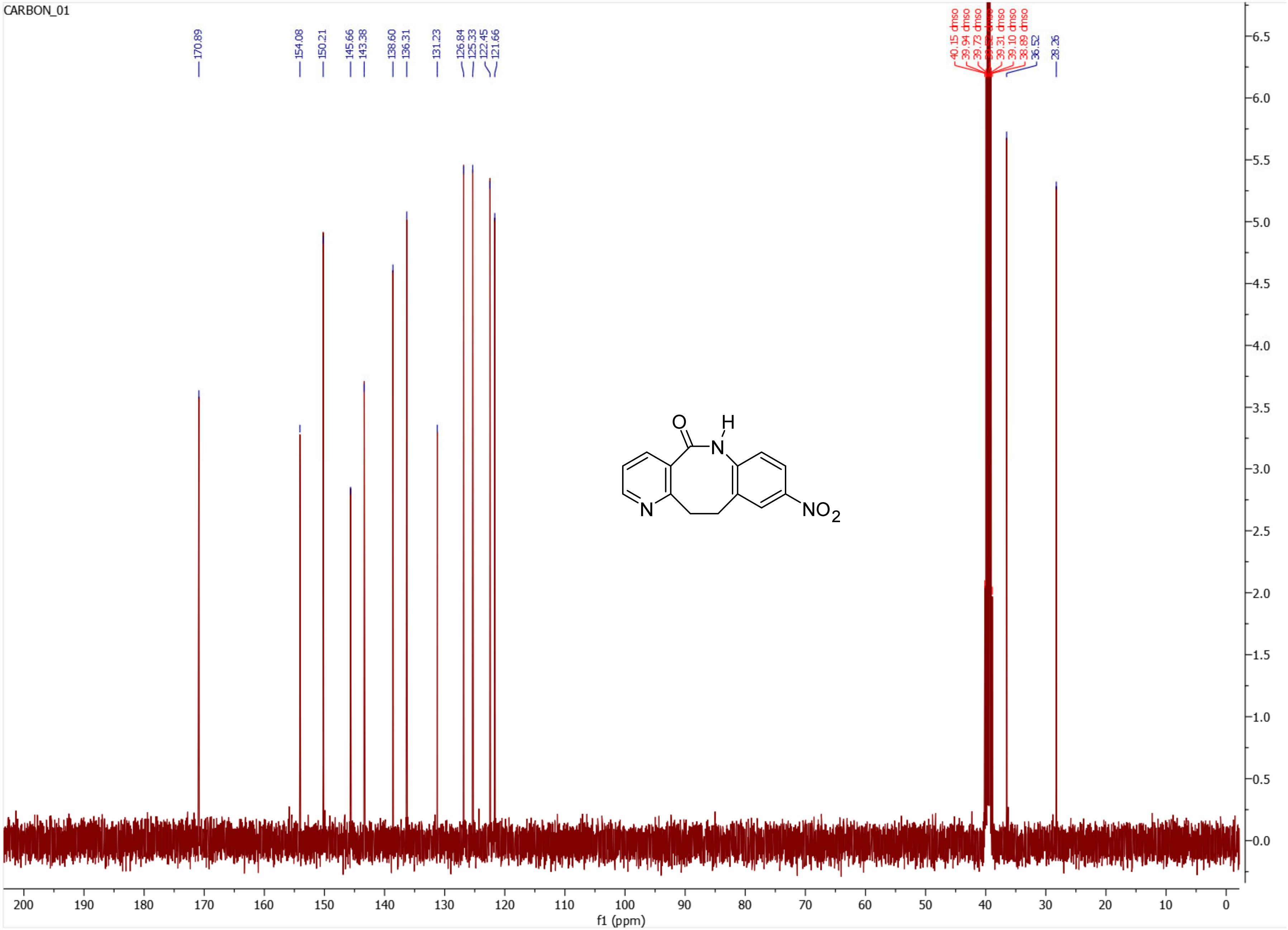

Figure S35. ^{13}C NMR spectrum (DMSO-d_6) of 9-nitro-11,12-dihydropyrido[3,2-*c*][1]benzazocin-5(6*H*)-one (**31**)

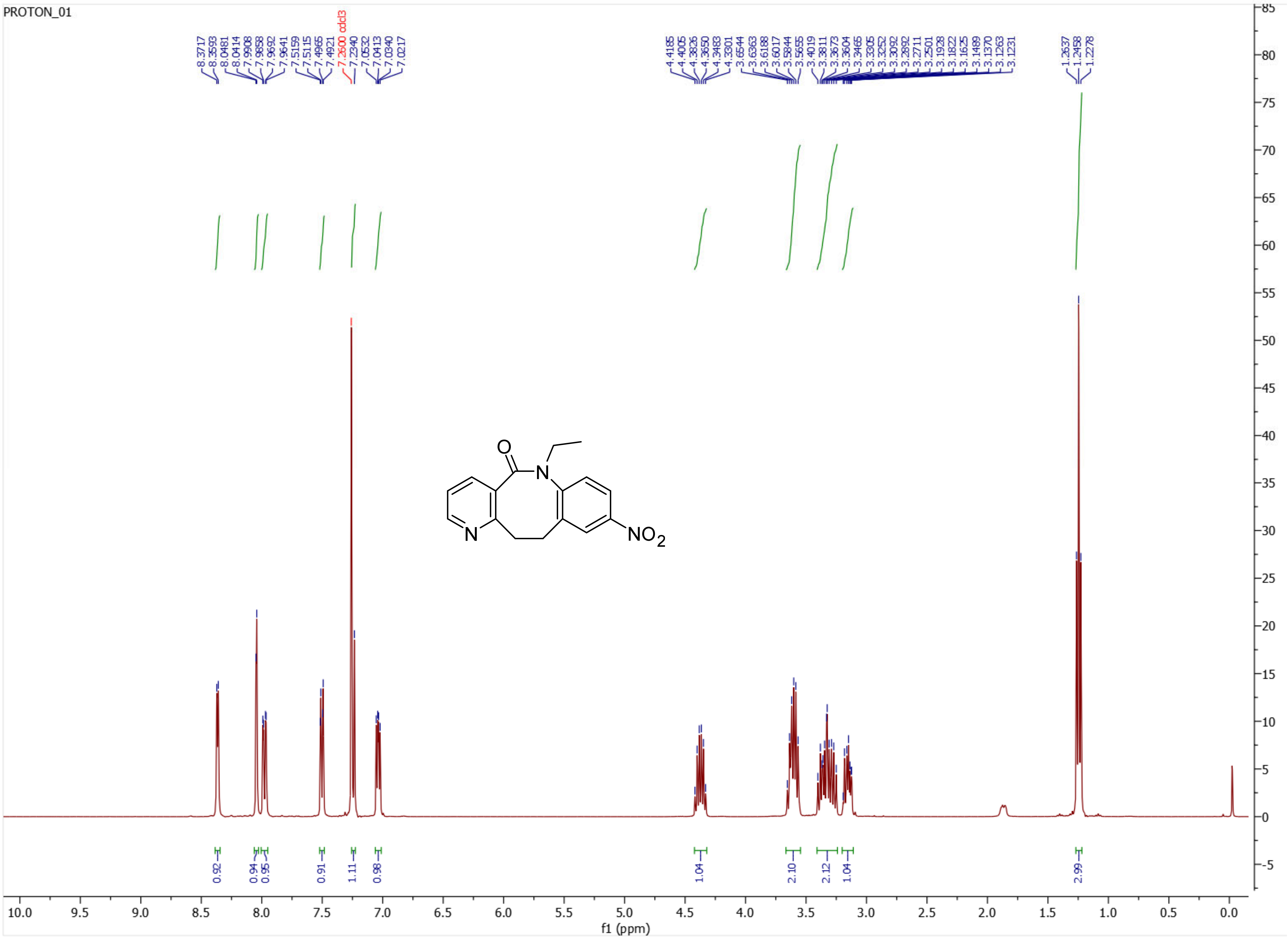

Figure S36. ^{1}H NMR spectrum ($CDCl_3$) of ethyl 6-ethyl-9-nitro-11,12-dihydropyrido[3,2-*c*][1]benzazocin-5(6*H*)-one (**32**)

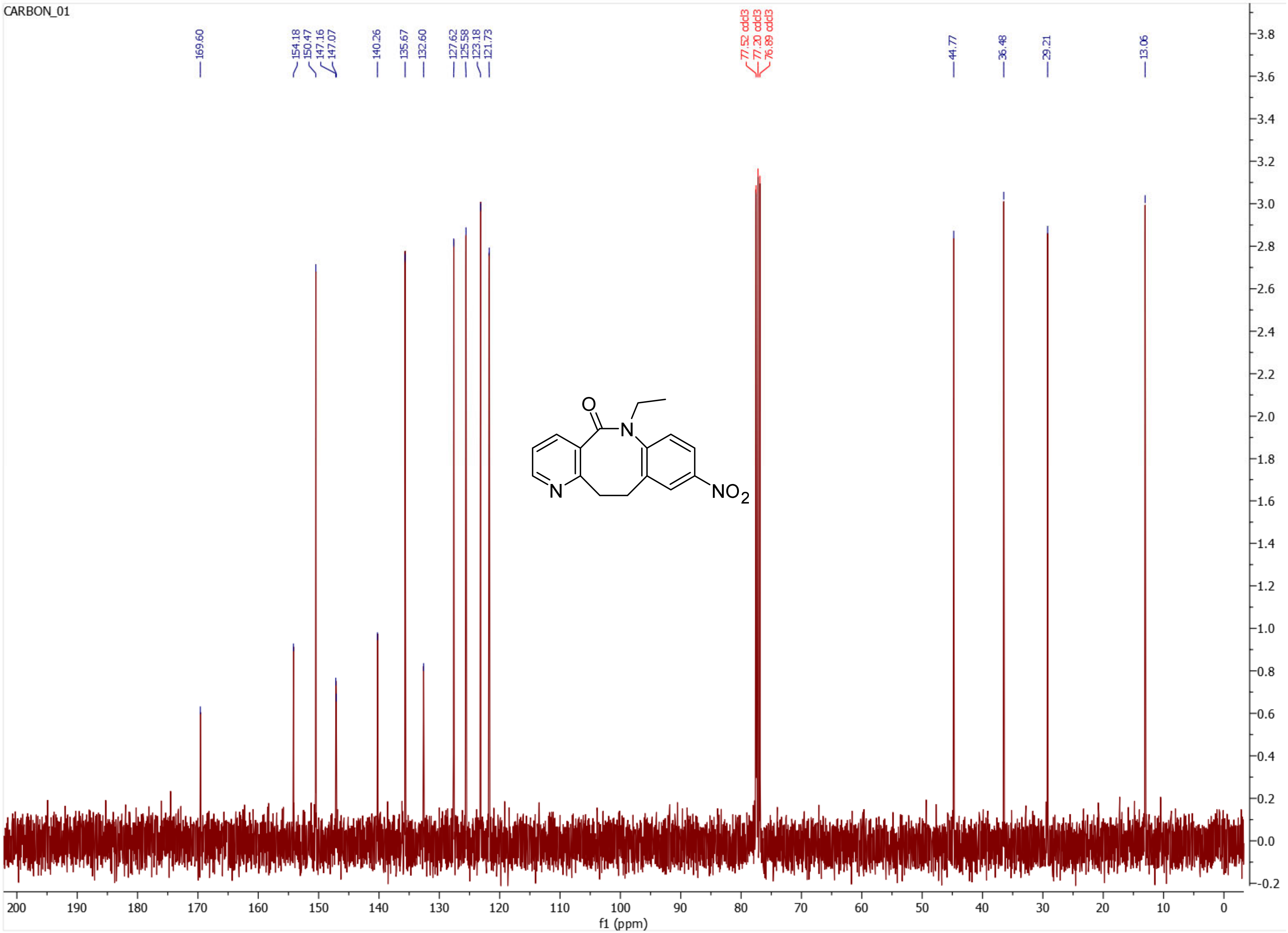

Figure S37. ^{13}C NMR spectrum ($CDCl_3$) of ethyl 6-ethyl-9-nitro-11,12-dihydropyrido[3,2-*c*][1]benzazocin-5(6*H*)-one (**32**)

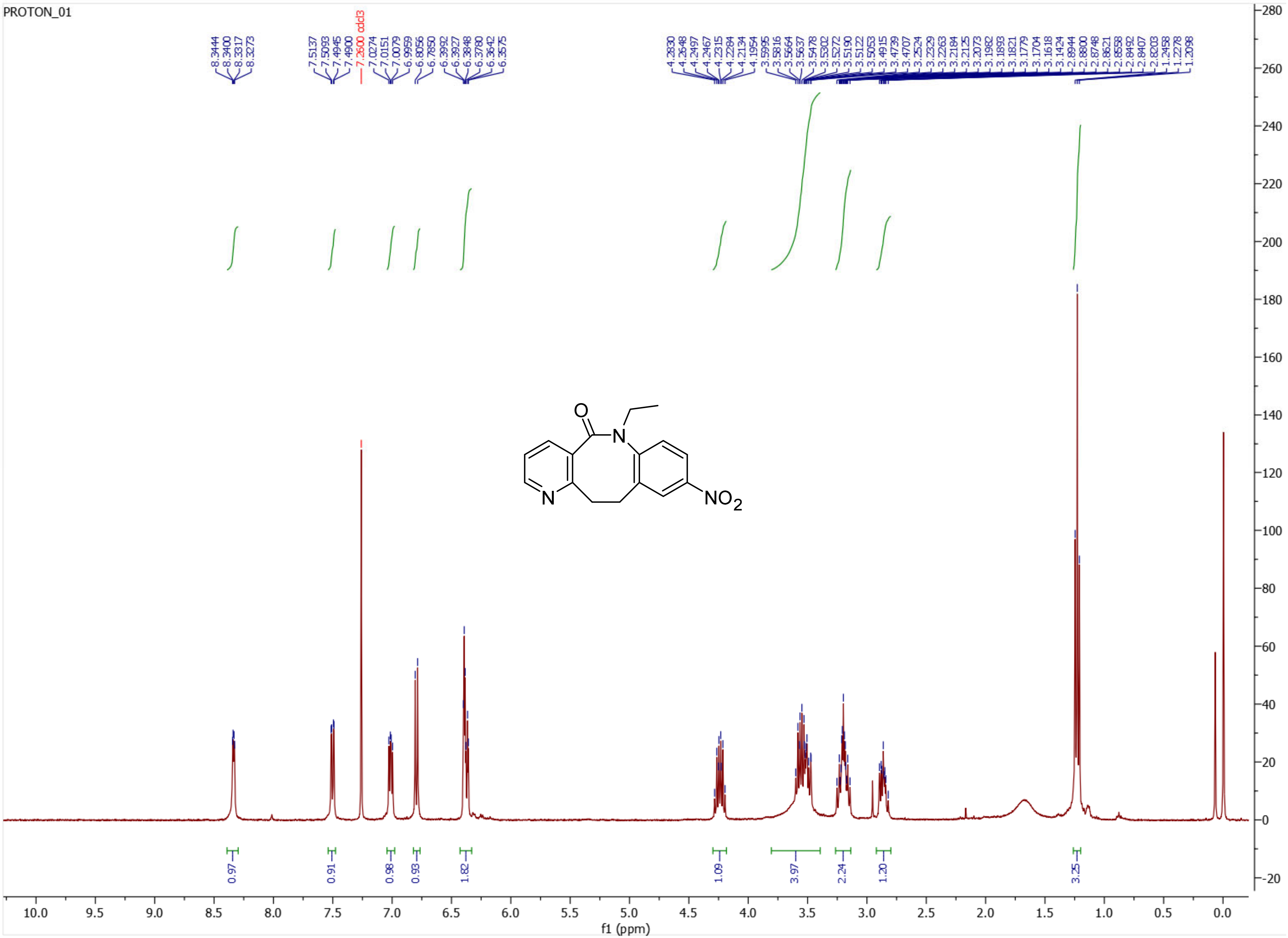

Figure S38. ^{1}H NMR spectrum ($CDCl_3$) of ethyl 9-amino-6-ethyl-11,12-dihydropyrido[3,2-*c*][1]benzazocin-5(6*H*)-one (**33**)

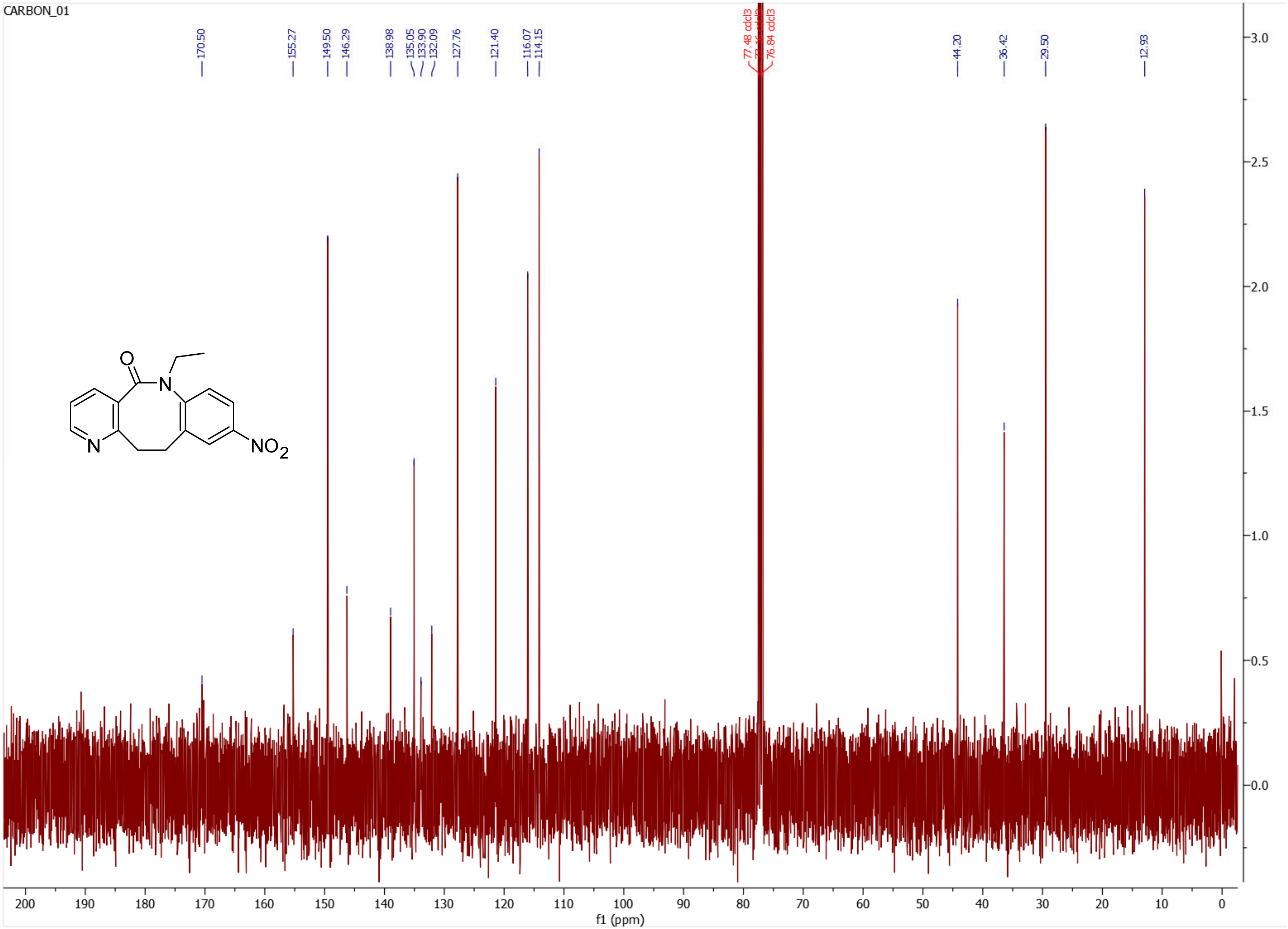

Figure S39. ^{13}C NMR spectrum ($CDCl_3$) of ethyl 9-amino-6-ethyl-11,12-dihydropyrido[3,2-*c*][1]benzazocin-5(6*H*)-one (**33**)

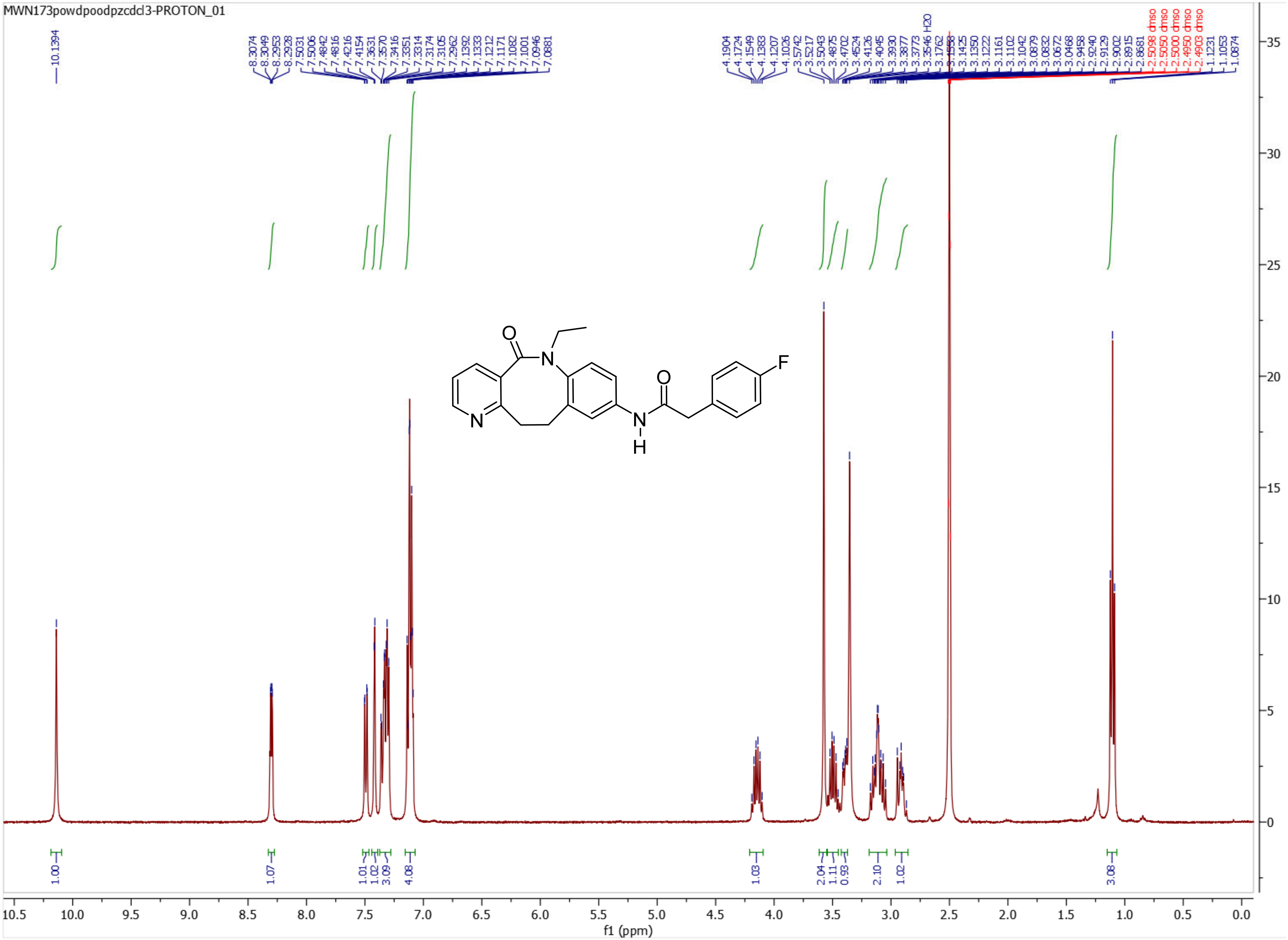

Figure S40. ^{1}H NMR spectrum (DMSO-d_6) of *N*-(6-ethyl-5-oxo-5,6,11,12-tetrahydrobenzo[*b*]pyrido[2,3-*f*]azocin-9-yl)-2-(4-fluorophenyl)acetamide (**10**)

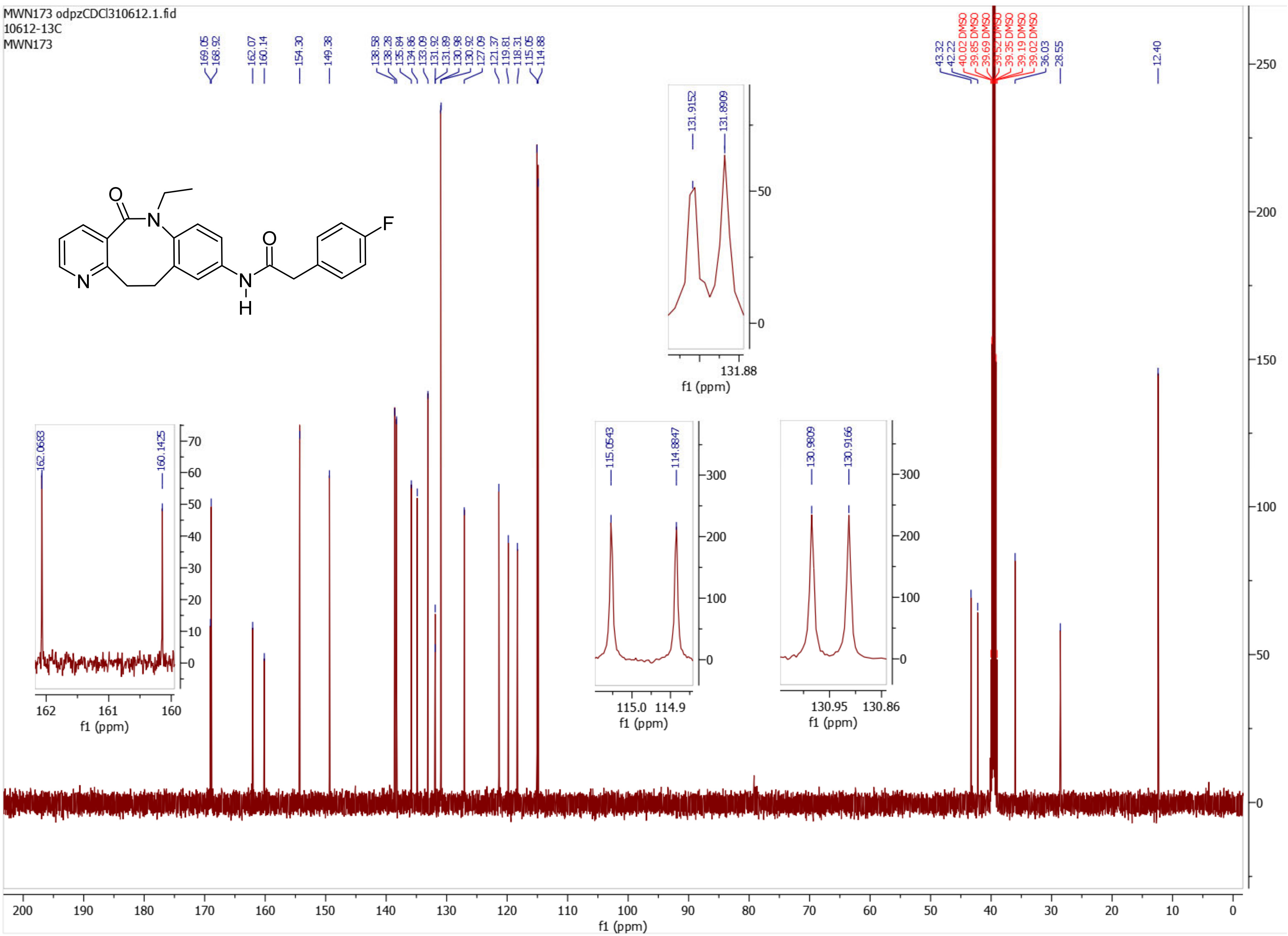

Figure S41. ^{13}C NMR spectrum (DMSO-d_6) of *N*-(6-ethyl-5-oxo-5,6,11,12-tetrahydrobenzo[*b*]pyrido[2,3-*f*]azocin-9-yl)-2-(4-fluorophenyl)acetamide (**10**)

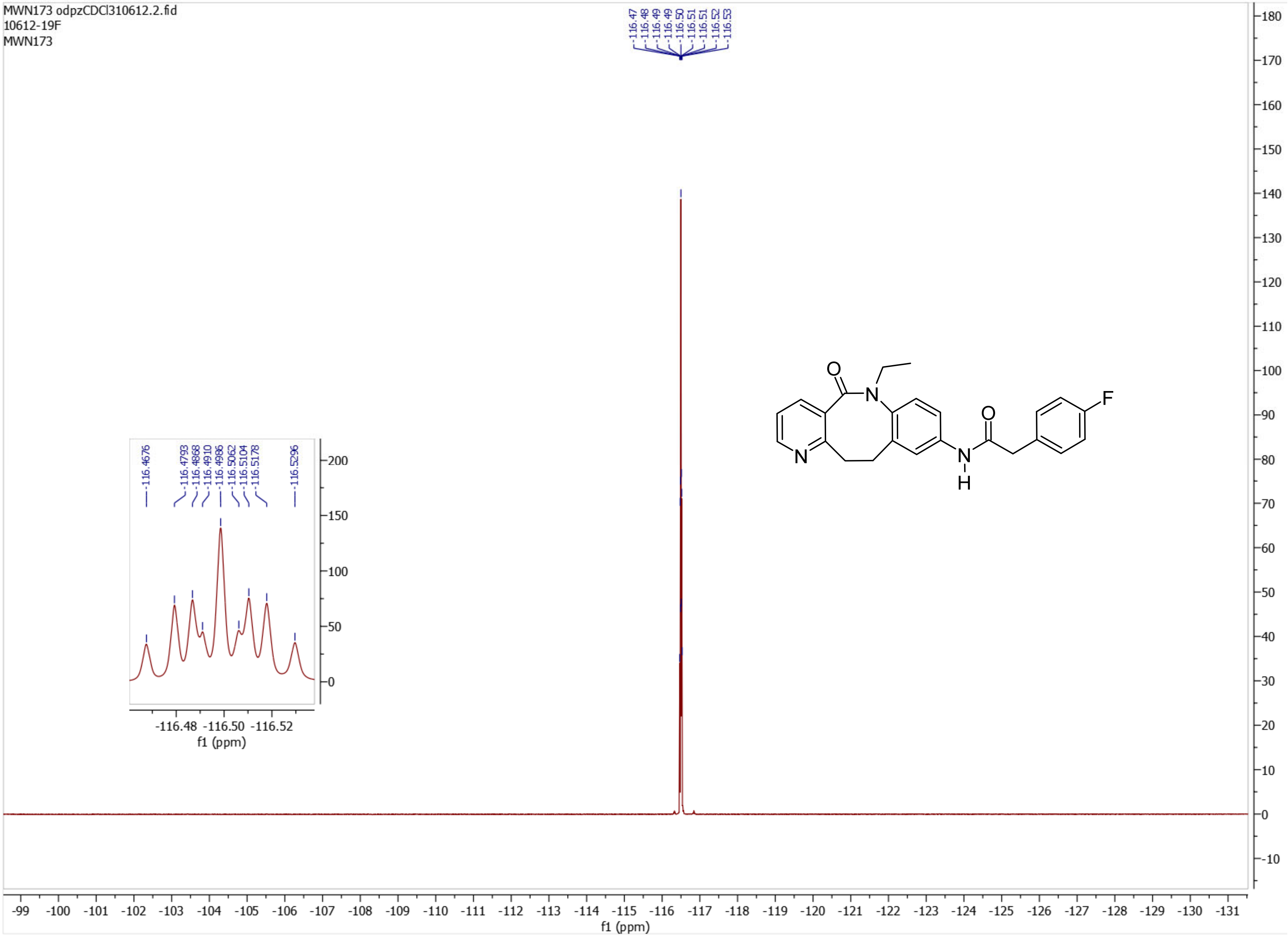

Figure S42. ^{19}F NMR spectrum in (DMSO-d_6) of *N*-(6-ethyl-5-oxo-5,6,11,12-tetrahydrobenzo[*b*]pyrido[2,3-*f*]azocin-9-yl)-2-(4-fluorophenyl)acetamide (**10**)

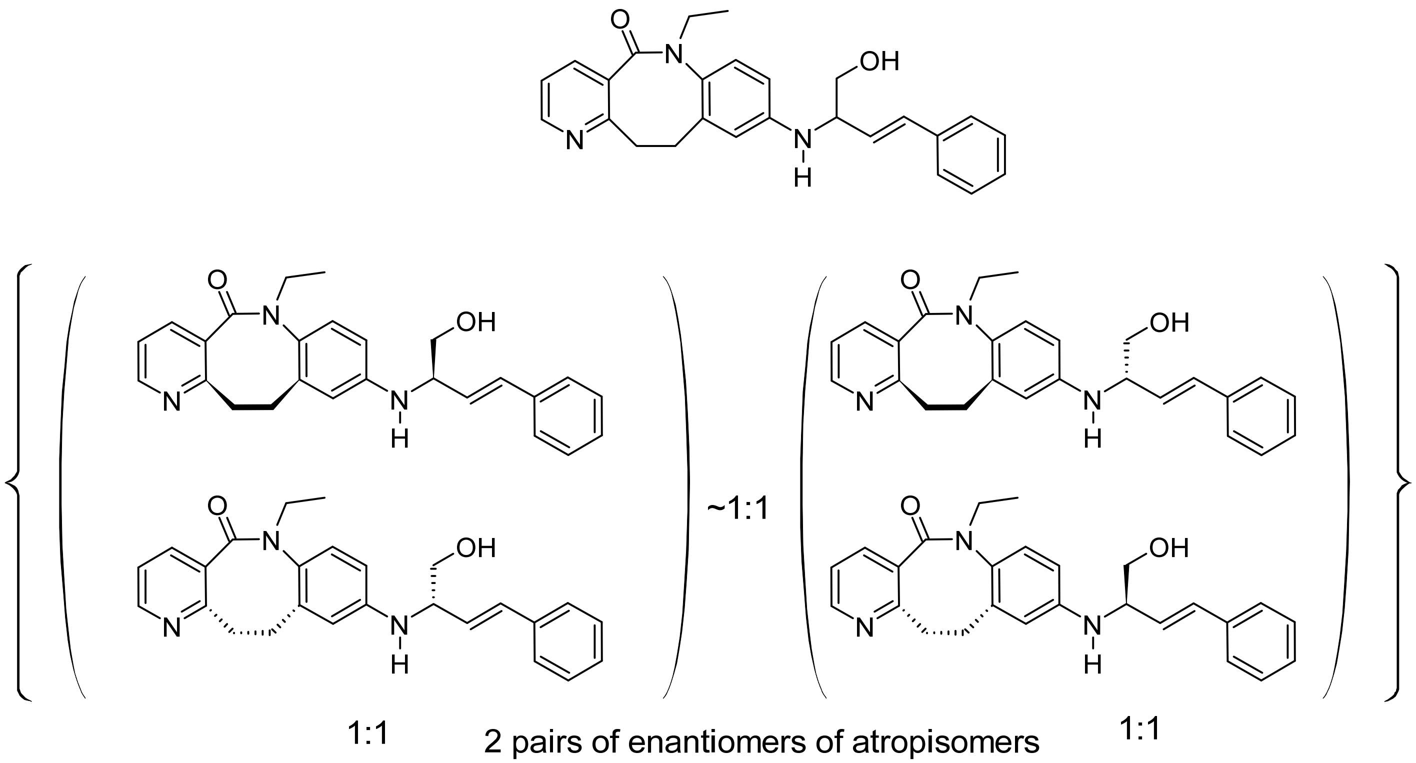

Figure S43. Structures of stereoisomers of (*E*)-6-ethyl-9-((1-hydroxy-4-phenylbut-3-en-2-yl)amino)-11,12-dihydrobenzo[*b*]pyrido[2,3-*f*]azocin-5(6*H*)-one (**12**) explaining doubled signals in ^{1}H and ^{13}C NMR

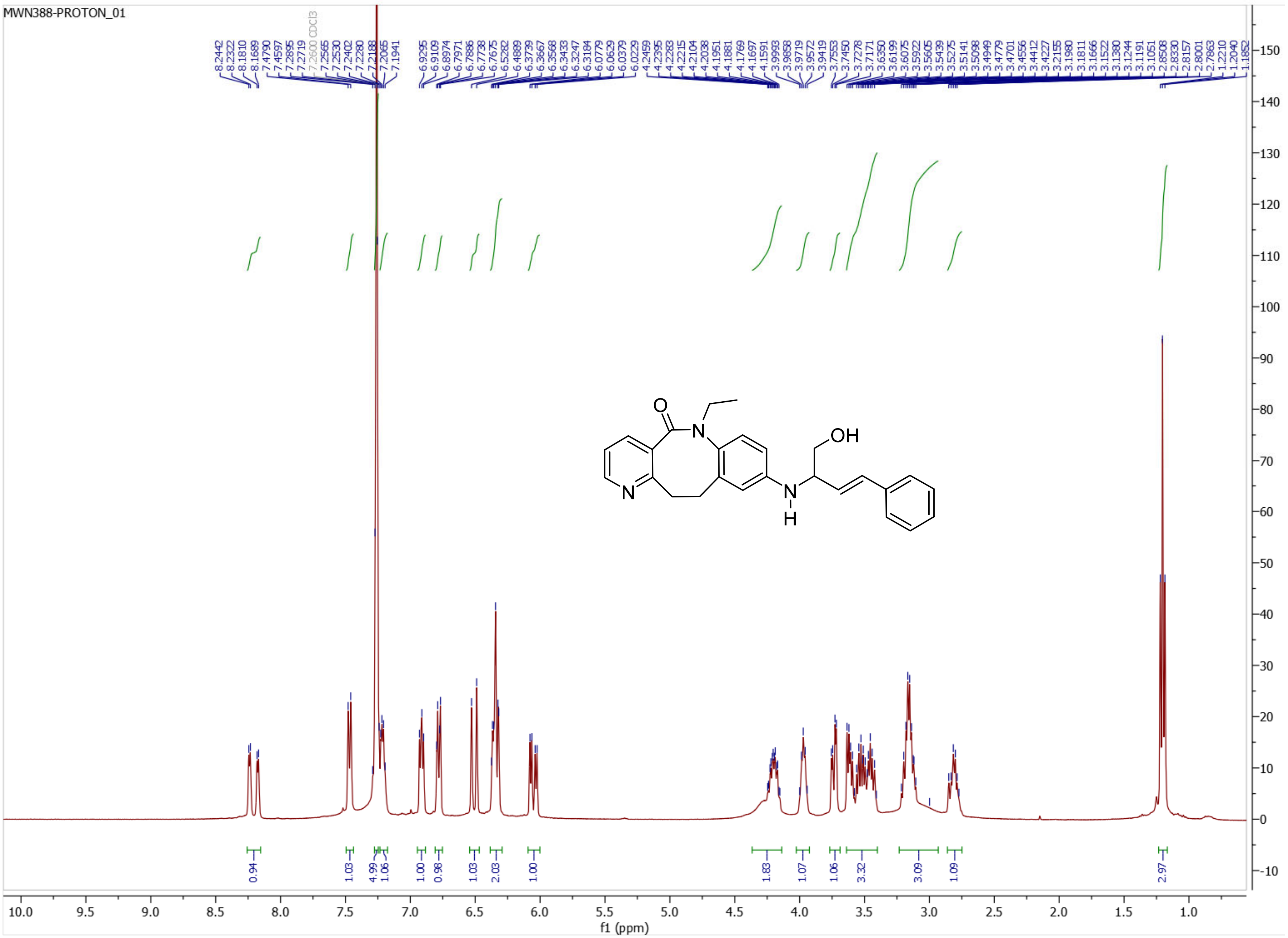

Figure S44. ^{1}H NMR spectrum ($CDCl_3$) of (*E*)-6-ethyl-9-((1-hydroxy-4-phenylbut-3-en-2-yl)amino)-11,12-dihydrobenzo[*b*]pyrido[2,3-*f*]azocin-5(6*H*)-one (**12**)

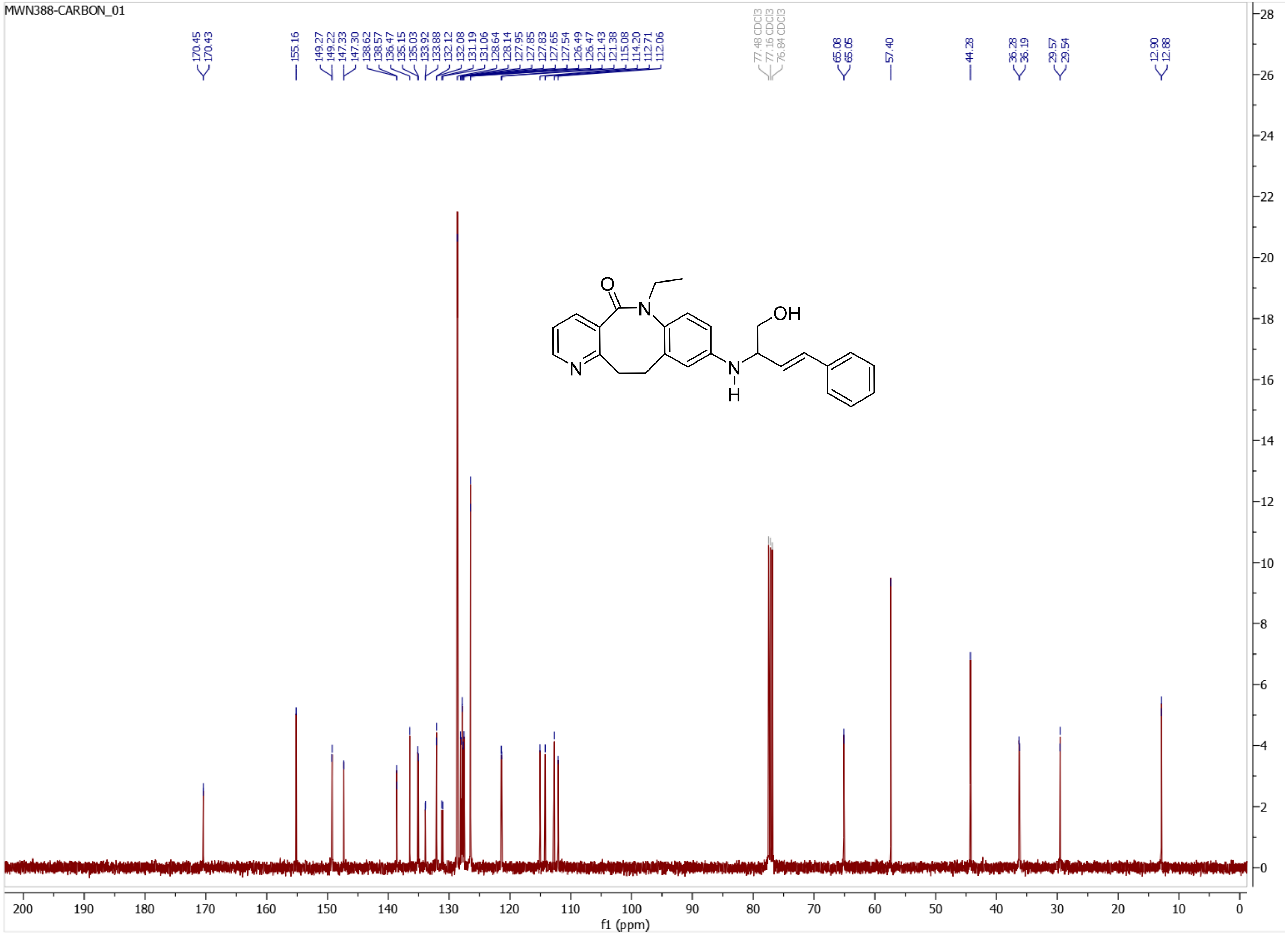

Figure S45. ^{13}C NMR spectrum ($CDCl_3$) of (*E*)-6-ethyl-9-((1-hydroxy-4-phenylbut-3-en-2-yl)amino)-11,12-dihydrobenzo[*b*]pyrido[2,3-*f*]azocin-5(6*H*)-one (**12**)
